# Beyond Latency: Chronic *Toxoplasma* Infection and Its Unveiled Behavioral and Clinical Manifestations—A 30-Year Research Perspective

**DOI:** 10.3390/biomedicines13071731

**Published:** 2025-07-15

**Authors:** Ashkan Latifi, Jaroslav Flegr

**Affiliations:** Department of Philosophy and History of Sciences, Faculty of Science, Charles University, Viničná 7, 128 00 Prague, Czech Republic; ashkan.latify@gmail.com

**Keywords:** parasite, manipulation hypothesis, evolution, mental health, RhD, Rhesus D antigen, testosterone, schizophrenia, OCD, autism, intelligence, subclinical toxoplasmosis

## Abstract

Over the past three turbulent decades, research has profoundly reshaped our understanding of chronic *Toxoplasma gondii* infection—traditionally regarded as harmless in immunocompetent individuals—unveiling its surprising impact on human health, performance, and behavior. This review emphasizes the effects of chronic *Toxoplasma* infection on physical and mental health, cognitive performance, and behavioral changes, highlighting key findings from studies investigating these domains, with a particular focus on both ultimate and proximate mechanisms underlying the observed effects. To this end, the primary focus will be on human studies; however, animal model studies will also be thoroughly considered when necessary and appropriate, to provide context and additional important information. Research demonstrates that chronic *Toxoplasma* infection may contribute to a broad spectrum of physical health issues. Ecological studies have revealed correlations between toxoplasmosis prevalence and increased morbidity and mortality from various conditions, including cardiovascular diseases, neurological disorders, and certain cancers. Large-scale cross-sectional studies have further shown that infected individuals report a higher incidence of numerous health complaints and diagnosed diseases, suggesting a significant impact on overall physical well-being. In addition to physical health, lifelong *Toxoplasma* infection (subclinical toxoplasmosis) has been implicated in cognitive impairments and behavioral changes. Studies have reported associations between infection and poorer performance in areas such as reaction time, processing speed, working memory, and executive function. Many of these behavioral changes likely relate to worsened health and a shift towards a “fast life history strategy.” These cognitive deficits can have significant implications for daily functioning and performance. Furthermore, the role of *Toxoplasma* infection in the development or exacerbation of mental health disorders has been extensively investigated. Meta-analyses, ecological studies, and large-scale observational studies have demonstrated associations between *Toxoplasma* infection and an increased risk of disorders such as schizophrenia and obsessive–compulsive disorder. While the precise mechanisms underlying these associations remain under investigation, research suggests that neuroinflammation and alterations in neurotransmitter systems are likely to play a role. Far from being harmless, subclinical toxoplasmosis is increasingly recognized as a hidden factor influencing human health, behavior, and cognitive performance—with implications that extend well beyond the individual to public health at large. Further research is warranted to elucidate the complex interplay between *Toxoplasma* infection, host physiology, and the development of various physical, cognitive, behavioral, and mental health conditions.

## 1. Introduction: *Toxoplasma gondii*

*Toxoplasma gondii* (Nicolle & Manceaux, 1908) is an obligate intracellular coccidian parasite capable of infecting a wide range of nucleated cells, including those of the musculoskeletal system, nervous system, and retina. A substantial proportion of the global population encounters this parasite, resulting in a probably life-long infection. Felids are the only known definitive hosts of *Toxoplasma*, where the parasite undergoes sexual reproduction following the ingestion of an infected intermediate host—a process known as trophic transmission. Within the feline intestinal epithelium, the parasite produces unsporulated oocysts that are excreted in the feces. Once exposed to air in the environment for several days, these oocysts undergo sporulation, during which they develop into infectious forms containing sporocysts, each housing sporozoites. Upon entering a new host, the parasite rapidly proliferates as asexual tachyzoites, which invade host cells and characterize the acute phase of toxoplasmosis.

This table provides a comprehensive overview of the five clinically distinct forms of *Toxoplasma* infection observed in humans, differentiating them based on their typical clinical course, duration, primary symptoms, and overall clinical relevance. It highlights the varying presentations from acute, self-limiting infections to severe, life-threatening conditions like congenital and cerebral toxoplasmosis, and introduces the concept of subclinical toxoplasmosis (formerly referred to as latent toxoplasmosis) to better describe the long-term, often subtle, symptomatic phase in immunocompetent individuals. This classification addresses the historical inconsistencies in nomenclature and emphasizes the active, though often unrecognized, impact of subclinical *Toxoplasma* infection.

Together with acute toxoplasmosis, there are five clinically distinct forms of the disease caused by *Toxoplasma* infection in humans, differing in their course and prognosis (Table 1). When the acute stage of toxoplasmosis develops in pregnant women, there is a significant risk of transmitting the infection to the fetus. This transmission can lead to miscarriage or result in congenital toxoplasmosis in the newborn. Congenital toxoplasmosis is the most clinically impactful form of the disease, with potential outcomes including encephalitis, chorioretinitis, hydrocephalus, microcephaly, and intracerebral calcification. Additionally, it may lead to less severe sequelae such as sensory and cognitive deficits in children. However, severe or even fatal outcomes, such as the severe inflammation of the heart leading to heart failure, brain inflammation, enlarged spleen, inflammation of the retina and choroid, and lung inflammation, can also occur in other high-risk groups, particularly immunocompromised individuals (e.g., AIDS patients or transplant recipients). The severity and specific outcomes of congenital toxoplasmosis are influenced by factors such as the stage of pregnancy during infection, the intensity of the infection, and the virulence of the *Toxoplasma* strain. Comprehensive overviews on this topic can be found in the works of Johnson and Johnson [1], Wesołowski et al. [2], and Tong et al. [3].

In postnatal infections of immunocompetent hosts, the immune response typically compels the parasite to form tissue cysts containing slowly dividing bradyzoites. Consequently, the acute stage of toxoplasmosis in immunocompetent individuals is generally transient. The presence of these tissue cysts, containing the dormant bradyzoite stage of *Toxoplasma*, characterizes the latent or subclinical form of the disease. This condition often persists for life. Only rarely, likely in subjects with specific genetic predispositions or unique immune system characteristics, does acute toxoplasmosis progress into a chronic form of the disease instead of its latent state. This chronic form is distinguished by a persistent or recurring range of diverse clinical symptoms. In immunocompromised individuals, such as transplant recipients, patients undergoing chemotherapy for oncological diseases, or AIDS patients, the infection can reactivate. This may lead to the development of life-threatening cerebral toxoplasmosis (toxoplasmic encephalitis), which requires immediate medical intervention. The progression of toxoplasmosis and its clinical manifestations have been extensively described in previous studies [4,5].

**Figure 1:**
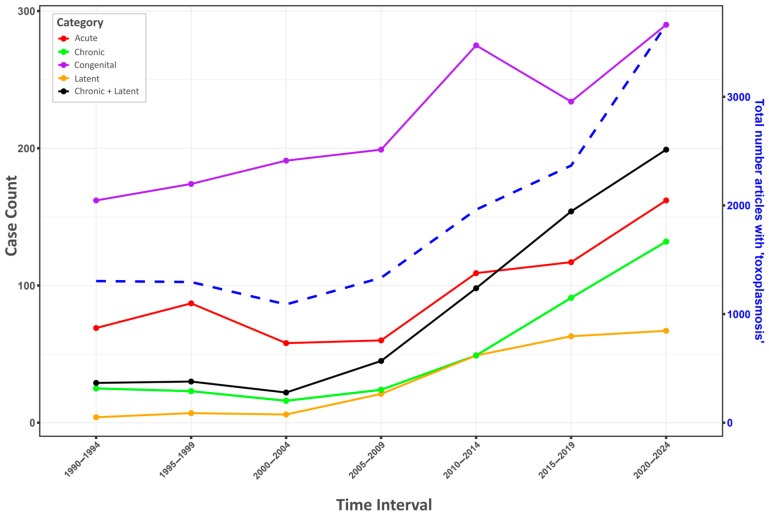
Trends in the usage of *Toxoplasma* terminology in PubMed (1990–2024). This figure illustrates the temporal trends in the number of articles containing specific *Toxoplasma* terminology in their titles, abstracts, and keywords within the PubMed database across 5-year intervals from 1990 to 2024. The primary *Y*-axis (“Case Count”) indicates the number of articles for categories such as acute, chronic, congenital, latent, and a combined “Chronic + Latent” group. The secondary *Y*-axis (“Total number articles with ‘toxoplasmosis’”) shows the overall publication output related to the parasite (blue dashed line). The graph highlights a substantial increase in the usage of terms related to long-term infection: the occurrence of articles containing the term ‘latent toxoplasmosis’ in their title, abstract, and keywords rose from 4 during the period 1990–1994 to 67 during the period 2020–2024, representing approximately an increase of 1575%. The number of articles containing the term ‘chronic toxoplasmosis’ climbed even higher, from 25 to 132 during the same period, an increase of approximately 428%. However, a visual inspection of the articles containing the term “chronic toxoplasmosis” indicated that a significant portion of them actually concerned latent (subclinical) toxoplasmosis, rather than the relatively rare chronic toxoplasmosis. It is noteworthy that during the same period, the total number of articles containing the term ‘toxoplasmosis’ in their title, abstract, and keywords increased from 1304 to 3663, which is only approximately 181%.

The mildest form of the disease from a clinical perspective is usually referred to in the literature as latent toxoplasmosis, or sometimes chronic toxoplasmosis (see Figure 1). Neither of these names is entirely suitable. Chronic toxoplasmosis typically designates a form of the disease characterized by overt, often severe, symptoms of acute toxoplasmosis that persist or recur for months or years. The term latent toxoplasmosis, on the other hand, implies that infected individuals show no clinical symptoms whatsoever. The most appropriate designation would probably be subclinical toxoplasmosis [6,7]; however, this term appeared only in five articles (in titles, abstracts, and keywords of articles) in the PubMed database between 1990 and 2025. In the current study, for simplicity, we will adhere to the more common, though factually incorrect, term latent toxoplasmosis, despite this review’s main message being to demonstrate that this most common form of the disease is far from latent and, in a substantial proportion of infected individuals, is likely not even subclinical.

**Figure 2:**
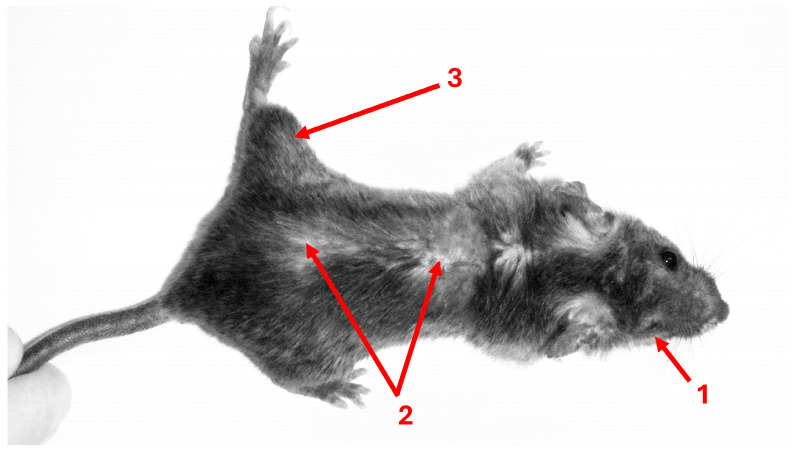
*Toxoplasma*-infected mouse 18 months after laboratory infection. Female F1 crosses, resulting from BALB/c females and C57BL males, were infected with an avirulent HIF strain of *Toxoplasma gondii* at 10 weeks of age. No differences in appearance or weight were observed between infected and control mice following the resolution of the relatively mild acute toxoplasmosis, which occurred 7 weeks post-infection. After 18 months, infected mice—but not the non-infected controls—exhibited multiple health problems, including eye loss (1), skin lesions (2), and limb paralysis (3). In addition, necropsy revealed markedly enlarged spleens in all infected individuals. (Photo: J.F.; image reproduced with permission from the publisher of reference [18]).

The ‘manipulation hypothesis’ has historically played a central role in latent toxoplasmosis research. It posits that the parasite may modify the host’s phenotype, typically its behavior, to enhance its own transmission. These modifications are proposed to represent the parasite’s evolutionary adaptations rather than merely the side-effects of the infection [8,9]. Numerous experimental studies support the idea that such behavioral changes can indeed facilitate the transmission of *Toxoplasma*. For instance, *Toxoplasma*-infected rats exhibit increased activity [10] and reduced neophobia [11], making them more easily trapped compared to uninfected individuals. Similarly, infected mice display hyperactivity in the open field [12,13], along with impairments in motor coordination and performance [14,15]. There is also evidence suggesting that *Toxoplasma* infection compromises cognitive functioning in mice. Infected animals show deficits in learning and memory, including a reduced responsiveness to novel stimuli such as unfamiliar objects [15]. Piekarski et al. [16] reported significantly worse learning performance in mice four weeks post-infection, and Witting [17] observed a similar deficit in learning capacity in both mice and rats, although it was more conspicuous in the former. A critical issue when studying the manifestations of toxoplasmosis in mice (compared to similar studies in rats or humans) is that mice are highly susceptible to toxoplasmosis. Even in resistant strains of mice or their even more resistant crossbreeds infected with *Toxoplasma* strains, the disease probably never fully resolves. Alternatively, new and severe symptoms of the disease, e.g., blindness, skin lesions, limb paralysis, and splenomegaly (see Figure 2) can appear many months after the apparent resolution of the initial illness [18]. Mice are therefore likely not a suitable model for studying latent toxoplasmosis, and it is probable that many behavioral manifestations described in this model are instead symptoms of chronic toxoplasmosis.

Studies on toxoplasmosis and behavior modification began to focus on human infection in the mid-1990s. Pioneering studies in this area of research, investigating *Toxoplasma*-infected and uninfected individuals using the Cattell’s 16PF (form A), found that toxoplasmosis affected personality in the infected individuals [19,20]. These studies also found significant positive associations between the duration of infection and the observed effects on personality.

An important area of research in the study of latent (subclinical) toxoplasmosis is its impact on the health of infected individuals, which is guided by two broad primary theories, one of which is based on the manipulation hypothesis. The first theory posits that the adverse effects of the disease stem from the host’s physiological response to the infection. This theory suggests that alterations in hormonal levels, particularly testosterone [21], as well as changes in neurotransmitter levels and activity—such as dopamine [22], serotonin [23], norepinephrine [24], and glutamate [25]—may contribute to various health outcomes. These include physical health problems [26,27] and mental health disorders such as schizophrenia [28], bipolar disorder [29], anxiety [30], ADHD [31], as well as cognitive impairment [32]. Additionally, pathological damage to tissues indicated by biomarkers [33] is also argued to be primarily a consequence of the host’s immune response to the infection, and implicated in its adverse effects on health.

The second theory posits that certain behavioral, personality, and cognitive performance changes may primarily arise from the manipulations exerted by *Toxoplasma* on its hosts. Specifically, it is suggested that neurotransmitters like dopamine and hormones such as testosterone are directly influenced by the parasite, which in turn affects healthoutcomes. For example, dopamine is particularly related to mental disorders such as schizophrenia and bipolar disorder [34,35]. Research also indicated that variations in testosterone levels can indeed influence the sexual behavior of hosts potentially increasing the likelihood of sexual transmission of the parasite [36,37,38]. Furthermore, studies have demonstrated that *Toxoplasma* has the ability to synthesize L-Dopa within the brain, a precursor necessary for dopamine synthesis [39]. Alterations in dopamine levels have been implicated in various mental disorders and cognitive functions. Nonetheless, it is important to recognize that these two theories may not be mutually exclusive; rather, they could simultaneously elucidate the underlying pathophysiology associated with the observations of this infection in affected individuals.

It should be emphasized that our review article will primarily focus on the latent (subclinical) toxoplasmosis-associated effects on health in humans. However, to provide a broader context and to allow for a more objective assessment of the nature of these effects, we will also address similar effects in animal hosts where relevant. Although from both clinical and economic perspectives, the impact of this form of toxoplasmosis on physical and mental health is of particular importance (Table S1, which summarizes the meta-analytical studies and reviews used in this review that examine *Toxoplasma* infection and its relationship with physical health, mental health, and cognition, is included in the ‘Supplementary Tables section’), research in humans has historically progressed in a different order—beginning with its influence on behavior and personality traits, then on cognitive performance, followed by mental health, and only more recently on physical health. The structure of this review will reflect this historical and conceptual progression.

## 2. Toxoplasmosis, Behavior and Personality

### 2.1. Toxoplasma and Behavior

#### 2.1.1. *Toxoplasma* and Behavior Change in Rodents and Mice

Studies on animal models have revealed that *Toxoplasma* can change the intermediate host’s behavior. Early studies on behavioral effects of *Toxoplasma* focused on toxoplasmosis in rodents. The findings were both surprising and suggestive at the same time. The parasite could modify the infected animal’s behavioral repertoire in interesting ways. For example, a study, using observational methods, investigating behavior in *Toxoplasma*-infected mice, found that the infection had selective effects on different behavioral categories in the infected animals. Both congenital toxoplasmosis and the subcutaneous injection of the infectious agent resulted in increased locomotor activity and decreased rearing and digging in infected rodents [10,12,13]. In addition, infected animals exhibited shorter bouts of behavior compared with controls. These studies suggested that these effects might be the result of infection-induced changes in the processing of the stimuli in the environment and attentional processes in infected mice [14,40]. Studies using the Y-maze design also showed that *Toxoplasma*-infected male mice were less responsive to novel stimuli. Specifically, these infected mice explored the previously inaccessible arm less than the controls once they had the opportunity to do so. Furthermore, they were significantly less active and produced fewer fecal boluses than controls [40,41]. These results suggested that impaired responsiveness to novel stimuli could make it more likely for the infected mice to be preyed on by predators [15,42]. In experiments, this impaired responsiveness manifests as a decrease in attraction to novel stimuli in neophilic species like mice and humans, and an increase in attraction to novel stimuli in neophobic species like rats.

Another study, which also investigated activity and preference for exposed areas in *Toxoplasma*-infected mice, examined their activity over a series of five trials, unlike earlier studies that had tested these behaviors in a single trial. The findings demonstrated that increased activity and decreased preference for novel areas were not merely transitory effects but actually intensified over the course of the trials. The researchers suggested that these behavioral changes could assist the parasite in meeting its life-cycle demands, particularly in the prey–predator relationship between the intermediate host (rodents) and the definitive host (domestic cats) [43]. Further research also showed that infection with this parasite could result in heightened activity in rats compared with the controls. One study specifically attributed this heightened activity to a *Toxoplasma*-induced manipulation, making the infected rats more likely to be spotted and hunted by cats [10]. In this regard, a recent study provided the long awaiting empirical evidence that *Toxoplasma*-infected mammals hunted by cats had indeed higher prevalence rates of *Toxoplasma* infection in the natural environment [44].

Later studies further demonstrated that *Toxoplasma* is capable of modifying the behavior of the intermediate host in subtler ways. For example, researchers investigating rats’ innate aversion to cat odor found that *Toxoplasma*-infected rats were significantly more likely than controls to frequent the corners of the pens containing fresh undiluted cat urine in comparison with the corners containing rat smell, neutral smell, or rabbit odor [45]. It has been shown that this effect (named fatal attraction) is highly specific and pertains only to the fear of natural hosts of *Toxoplasma*, not to the fear of other dangerous stimuli [46]. An important role in this phenomenon is played by the increased production of testosterone in infected animals [47], which modulates the activity of specific areas of the hypothalamus [21]. Simply put, certain stimuli that naturally induce fear in uninfected animals simultaneously evoke sexual arousal in infected ones [48].

#### 2.1.2. *Toxoplasma* and Behavior Change in Non-Human Primates

The manipulations by *Toxoplasma* are not limited to rodents, as studies have revealed that other species are also subject to these effects. Non-human primates are susceptible to *Toxoplasma* infection too. For example, studies showed that a relatively large proportion of non-human primates in captivity were infected with toxoplasmosis [49,50]. According to these studies, New-World non-human primates were specifically susceptible to the clinical form of the disease, whereas Old-World non-human primates were generally resistant and developed sub-clinical toxoplasmosis.

Leopards are reported to prey on apes [51,52]. In this regard, a study investigating the behavior of chimpanzees infected with *Toxoplasma* reported that these primates lost their innate aversion to leopard urine compared with uninfected controls. These researchers did not find such an effect in either infected or uninfected chimpanzees when they tested the urine of felines that chimpanzees were unlikely to encounter in nature (i.e., lions and tigers) [53]. It remains unknown why infected chimpanzees were not attracted to the urine of felines other than leopards. We can only speculate that the strain of the parasite may be relevant, presumably a strain evolutionarily tailored to the characteristics of chimpanzees, leopards (as their natural predator), and their coexistence in their habitat.

#### 2.1.3. *Toxoplasma* and Behavior Change in Humans

The effects of *Toxoplasma* infection on human real-life daily behavior have been rarely addressed by researchers. In this respect, a notable exception is the double-blind study by Lindová et al. [54], which investigated three composite behavioral variables—Self-Control and Clothes Tidiness, Relationships, and Mistrust—corresponding to ‘factors G—conscientiousness and Q3—self-control,’ ‘factor A—warmth,’ and ‘factor L,’ respectively, the personality factors affected by latent toxoplasmosis, see Section 2.2. The authors found sex-specific differences in behaviors between *Toxoplasma*-positive men and women, consistent with earlier findings based on personality questionnaires [19,20]. For Self-Control and Clothes Tidiness, there was a significant toxoplasmosis–sex interaction effect: infected men scored significantly lower than uninfected men, while an opposite trend was observed in infected women. Regarding Relationships, the toxoplasmosis–sex interaction effect was marginally non-significant (*p* = 0.053); uninfected men scored higher than infected men, but no difference was observed between infected and uninfected women. Considering Mistrust, a three-way interaction (toxoplasmosis–sex–place of residence) was observed. In the countryside, infected men scored higher than uninfected men (not significant though), but the pattern was the reverse in cities and significant. The trend was exactly the reverse for women and non-significant in both the city and countryside.

In addition, some articles also reported an association between latent toxoplasmosis and aggressiveness and impulsivity [55,56,57], see also Section 5.8. In particular, a study revealed that trait reactive aggression in women was significantly positively associated with *Toxoplasma* IgG seropositivity, although this effect was not present in the male group. In addition, impulsive sensation-seeking was significantly and positively associated with *Toxoplasma* seropositivity in younger men (20–59 years of age) [55].

The gravest real-life consequences of *Toxoplasma*-induced behavioral effects may occur in situations where rapid information processing and decision-making are critical, with traffic accidents being a prominent example. A study in the Czech Republic reported that infected subjects had 2.65 times higher a risk of accident than uninfected individuals; in addition, higher levels of anti-*Toxoplasma* antibody titers were associated with a higher risk of traffic accidents [58]. As the anti-*Toxoplasma* antibody titers lower as a function of the duration of infection over the lifetime, the authors suggested that this may possibly reflect a positive correlation between the intensity of infection and such consequences rather than a negative correlation between the duration of infection and these negative effects. Alternatively, the individuals with older infections could have evolved over the years the required strategies to compensate for their *Toxoplasma*-induced psychomotor functioning decline.

Another study undertaken in Turkey also found a significantly higher number of *Toxoplasma*-seropositive subjects in a group of participants with a record of traffic accidents while driving compared with a group of drivers without such a record [59]. A similar study in Russia also reported that the risk of road accident was 2.37 times higher in *Toxoplasma*-seropositive drivers than *Toxoplasma*-seronegative controls [60]. More recently, another study (a preprint), investigating the association between toxoplasmosis and the rate of traffic accidents in Iran, reported a significantly higher rate of accidents in the seropositive group compared with the seronegative group, which was also significantly higher in the subjects 35–69 years of age compared with younger ones [61]. Surprisingly, another recent Iranian study showed that the prevalence of toxoplasmosis among motorcyclists hospitalized due to traffic accidents was lower than in a control population of motorcyclists who were in the same hospital for reasons unrelated to traffic accidents [62]. The authors speculate that the consequences of traffic accidents for infected drivers may be more severe, possibly fatal, preventing them from providing informed consent to be included in the study.

Also on the ecological level, a study investigating the role of *Toxoplasma*-infection in the incident of traffic accidents across 87 countries, found that there was a positive and strong relationship between anti-*Toxoplasma* antibodies seroprevalence and the rate of traffic accidents resulting in disabilities; nevertheless, controlling for wealth, geographical latitude, health of population, length of roads, and number of vehicles resulted in the disappearance of this association [63]. Although the further inclusion of the frequency of RhD negativity—a protein found on red blood cells, individuals with it are Rh-positive; those without it are Rh-negative; in addition, it is widely known to be important in blood transfusions and pregnancy due to immune compatibility, and its interaction with the infection in the model restored the association; the ridge regression analysis of the data—used to address multicollinearity—resulted in the disappearance of this relationship once again. In examining the issue of multicollinearity, the authors concluded that a strong correlation between the prevalence of *Toxoplasma* infection and general population health in certain countries was responsible for the observed pattern of results. This correlation confounded the estimated effect *Toxoplasma*-seropositivity on traffic accident rates and, more broadly, pointed to the potentially detrimental impact of the infection on overall health.

A meta-analysis on nine eligible studies concluded that *Toxoplasma*-infection is a significant predictor and important risk factor for traffic accidents. Subgroup analysis according to age and sex in this meta-analysis showed a notable difference between the traffic accident group and the control group (no traffic accident) for both men and women. Specifically, for individuals under the age of 45, there was a clear distinction between these two groups. In contrast, for those aged 45 and older, no significant difference was observed between the groups [64]. Another recent meta-analysis conducted in accordance with PRISMA guidelines and based on data from Medline, PubMed, and PsychInfo, also reported a significant association between anti-*Toxoplasma* antibodies and traffic accident (odds ratio = 1.69; 95% CI 1.20–2.38) [65].

Accident rates at work have also drawn researchers’ interest due to the importance of cognitive measures such as information processing speed, reaction time, psychomotor accuracy, and concentration in the workplace. In this context, a study in Mexico found a significant positive association between *Toxoplasma* infection and the rate of workplace accidents [66]. Their analysis revealed a significantly higher seroprevalence of *Toxoplasma*-infection among patients with workplace accidents and low socioeconomic status than controls of the same socioeconomic status (odds ratio = 3.38; 95% CI: 0.84–16.06).

Beyond its impact on accident risk, *Toxoplasma* infection has also been studied for its potential influence on other aspects of human behavior and decision-making in everyday life. For instance, one study investigating the choice of academic majors found that *Toxoplasma*-infected individuals were more likely to major in business, particularly in management and entrepreneurship. The study reported that *Toxoplasma*-infected individuals were more likely to choose business-related fields, especially management and entrepreneurship. Additionally, the researchers found that infected professionals were more likely to have started their own businesses. They attributed this trend to a lower “fear of failure” in infected individuals, as suggested by global studies showing a link between infection prevalence and entrepreneurial activity at the national level [67].

Future investigations should particularly prioritize longitudinal cohort studies to establish temporal relationships between *Toxoplasma* infection and workplace accident risk. Additionally, contextualized experimental designs employing neurocognitive assessments, tailored to the individual’s occupational demands, could help quantify the cognitive impairments linked to seropositive status in the context of workplace. Targeted populations should include workers in high-risk occupational settings (e.g., construction, transportation, manufacturing, medical health staff, etc.), with special attention to those from low socioeconomic backgrounds, who may face compounded vulnerabilities. Finally, the integration of emerging technologies, such as wearable neurocognitive monitoring devices, mobile cognitive testing apps, or even machine learning models trained on biometric and serological data, could enhance the precision and scalability of future studies.

### 2.2. Toxoplasma and Personality

#### 2.2.1. *Toxoplasma* and Cattell’s 16PF

Early studies on personality showed that infection with latent toxoplasmosis in humans can result in personality profile shifts that are moderated by sex. Two studies using Cattell’s 16 Personality Factor Questionnaire (16 PF) reported opposite trends of personality shift in some scales in infected men and women. In these studies, infected men scored significantly lower in factor G (Rule-Consciousness: Expedient vs. Rule-bound) and higher in factor L (Vigilance: Trusting vs. Suspicious) and non-significantly lower in factors A (Warmth: Reserved vs. Warm) and Q3 (Perfectionism: Tolerates disorder vs. Perfectionistic) [19,20]. A lower score in factor G means lower rule conscientiousness and expediency; hence, higher deviation from norms and conventions. A high score in factor L suggests dogmatism, suspiciousness, envy, and low tolerance. A low score in factor A indicates a critical and detached propensity and reservedness, while a low score in Q3 means carelessness, laxness, and impulsivity. However, for infected women, the scores were significantly higher in factor A and lower in factor L and non-significantly higher in factors G and Q3 [19]. Studies also showed that male individuals with older infections had larger degrees of personality factor shift [20]; a later study also reported the same for infected women [68]. Another study, using permutation test with data reassignment, also showed that *Toxoplasma*-positive women had significantly higher scores in factors B (Reasoning: Concrete vs. Abstract thinking)—a high score indicates abstract thinking and a quick learning ability, while a low score reflects concrete thinking and slower cognitive processing, and O (Apprehension: Self-assured vs. Apprehensive)—a high score suggests self-doubt, worry, and insecurity, whereas a low score indicates confidence, self-assurance, and emotional stability, and significant changes in the character distribution of factors L and Q3 [69].

Furthermore, research suggests that RhD phenotype —a blood group system, commonly known as the Rh factor— is another important factor in *Toxoplasma*-induced modifications. A study, among its other findings, also showed that the RhD phenotype affected the personality profile of *Toxoplasma*-positive subjects [70]. In this study, the interaction between toxoplasmosis and RhD phenotype was significant in factors C (Emotional Stability: Reactive vs. Emotionally stable)—where a high score signifies emotional resilience, calmness, and steadiness under stress, whereas a low score implies emotional reactivity, moodiness, and difficulty coping; M (Abstractedness: Practical vs. Imaginative)— where a high score reflects imagination, daydreaming, and a tendency to get lost in thoughts, while a low score indicates practicality, focus, and grounded thinking; and Q4 (Tension: Relaxed vs. Tense)—where a high score indicates restlessness, impatience, and being tightly wound, while a low score reflects relaxation, patience, and an easygoing temperament (for all participants regardless of sex and for men), and B (for women) in Cattell’s 16 PF.

#### 2.2.2. *Toxoplasma* and NEO PI–R and TCI

Research on toxoplasmosis and personality is not limited to Cattell’s PF questionnaire studies. Using other instruments developed more recently than Cattell’s, a study found that male and female infected students had higher extraversion and lower conscientiousness scores, assessed by the Neuroticism–Extraversion–Openness Personality Inventory—Revised, compared with controls [71]. This study also reported that changes in conscientiousness were negatively associated with the duration of *Toxoplasma*-infection in males. Another study, using the same questionnaire, investigated the association between *Toxoplasma* infection and personality change in 502 Czech male soldiers and found that infected subjects scored significantly lower in Neuroticism, among other differences [72]. In this respect, an ecological study, investigating the effects of toxoplasmosis on aggregate personality at the population level, found that aggregate Neuroticism increased significantly with *Toxoplasma* prevalence [73]. Accordingly, this author argued that, among many other factors, this infection is likely responsible for higher trends of certain cultural characteristics such as uncertainty avoidance and masculinity at the societal level.

A study, using a modern psychobiological questionnaire, Cloninger’s Temperament and Character Inventory, found that *Toxoplasma*-infected subjects had significantly lower Novelty Seeking scores. Specifically, authors demonstrated lower scores in three out of the four subscales of this scale, namely Impulsiveness and Disorderliness (both significant) and Extravagance (approaching significance, *p* = 0.056) than the controls. In addition, the differences between *Toxoplasma*-infected and uninfected subjects were found to be negatively correlated with the estimated duration of infection [74]. However, earlier studies using Cattell’s 16PF had shown a positive correlation between personality shifts and the duration of *Toxoplasma* infection. Flegr and his colleagues attributed this discrepancy to the fundamentally different theoretical foundations of the two personality instruments. Cattell’s 16PF is based on trait theory and is designed to measure stable personality dimensions that develop gradually over the course of ontogeny and are relatively resistant to short-term physiological influences. In contrast, Cloninger’s Temperament and Character Inventory is grounded in neurobiological theory and includes traits—such as Novelty Seeking and Harm Avoidance—that are believed to reflect current levels of specific neurotransmitters, including dopamine and serotonin. According to the authors, *Toxoplasma* infection induces relatively rapid and lasting changes in neurotransmitter levels, which may cause persistent alterations in Cloninger’s temperament scales from the early stages of infection onward. Because these changes occur quickly and remain stable, there should be no correlation between personality scores and the actual duration of infection.

However, a spurious negative correlation may arise due to how infection duration is estimated. Individuals with a more intense infection—for example, due to a larger infectious inoculum—are likely to develop a higher cyst load and greater neurophysiological changes, including elevated dopamine levels, as well as stronger immune responses and higher concentrations of IgG anti-*Toxoplasma* antibodies. If antibody levels are then used as a proxy for infection duration, individuals with high antibody titers (i.e., more recent infections) will also show stronger personality changes, while those with low titers (interpreted as having longer infection duration) will show weaker changes. This statistical artifact may produce a negative correlation between personality change and estimated infection duration, even if the actual changes are stable and unrelated to time since infection.

#### 2.2.3. What Is Behind the Opposite Effects of *Toxoplasma* on the Personality of Men and Women?

**Figure 3:**
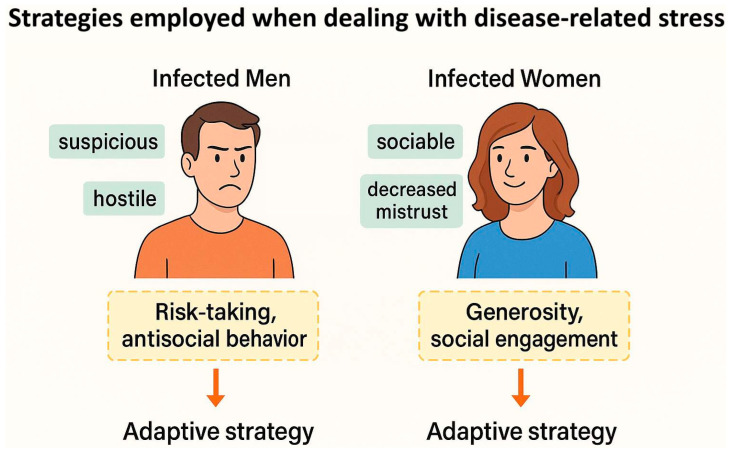
The Stress-Coping Hypothesis: a psychological explanation for sex-specific personality changes associated with latent toxoplasmosis.

*Toxoplasma* differently affects the personality shifts observed in *Toxoplasma*-positive men and women, as already discussed in the aforementioned studies. Researchers proposed three explanations (namely evolutionary, neurophysiological, and psychological explanations) for this observation [54]:

(1) Evolutionary explanation: according to the evolutionary perspective, drawing on the manipulation hypothesis, Lindová et al. [54] argued that *Toxoplasma* induces personality shift in the intermediate host in order to increase its transmission probability to the definitive host. In the ancestral environment in primitive human societies, being critical of others, aloof and detached, reserved, impulsive, lax, a violator of rules and social conventions, and untidily dressed would most likely result in the isolation of the males (behaving as such) from their group, rendering them more exposed and vulnerable to their natural predators such as lions, leopards, etc.; hence, increasing the transmission probability of the parasite to its final host. These researchers also proposed that an explanation for the personality shifts induced by *Toxoplasma* in females could be that it was to the parasite’s advantage to be congenitally transmitted to the infected females’ offspring, as there is evidence that activated latent toxoplasmosis in pregnant women can result in the transmission of *Toxoplasma* to the fetus, bringing about birth of children with asymptomatic toxoplasmosis. These researchers speculated that infected women could have been manipulated by the parasite to be more persistent, socially apt, conscientious, in control of themselves, tidily dressed, genial, and sympathetic in order to attract potential sexual partners more easily; hence, increasing the parasite’s transmission chance to the infected women’s offspring. However, this evolutionary hypothesis faces a fundamental problem: *Toxoplasma gondii* evolved primarily in nonhuman hosts—especially rodents—where such traits are unlikely to enhance reproductive success and parasite transmission. It is therefore difficult to see how natural selection could have favored manipulation mechanisms that produce these specific behavioral changes in human females. The hypothesis may help explain why traits that increase risk-taking or predation—commonly observed in infected males—are not similarly induced in females, who can transmit the parasite vertically to their offspring. However, it provides a less satisfactory explanation for why the infection appears to promote the development of seemingly opposite traits in females.

(2) Physiological explanation: although the underlying physiological mechanism in *Toxoplasma* personality shift remains unknown, the neurophysiological perspective puts emphasis on dopamine dysregulation in *Toxoplasma*-positive individuals. According to Lindová et al. [54], animal model studies point to the elevated levels of dopamine in infected mice’s brain. Human studies also suggest that modifications such as reduced novelty seeking in infected men may be caused by *Toxoplasma*-induced high dopaminergic baseline activity and postsynaptic downregulation. However, estrogen and progesterone are known to modulate the activity of dopamine in the nucleus accumbens and striatum and exert a neuroprotective effect on the dopaminergic system; hence, the different observed personality shifts in infected women. Accordingly, as these researchers maintain, it may be the net result of these physiological differences in *Toxoplasma*-infected men and women that may explain the less severe or even opposite personality shifts observed in infected women.

(3) Psychological explanation: according to Lindová et al., the third perspective proposes a psychological explanation which emphasizes the importance of the different strategies that men and women use to manage non-specific stressors such as toxoplasmosis (the Stress-Coping-Hypothesis) [54,75]. They discuss that men are known to employ problem-focused strategies while females implement emotion-focused ones. Accordingly, the personality shifts in infected men and women are expected to be in part based on their preferred coping strategies; as men withdraw from the society but women seek social integration and emotional expression when facing a stressor (see Figure 3). A study indirectly found empirical support for this psychological interpretation. Using the Trust Game and a modified version of the Dictator Game, as predicted by the psychological perspective, infected men were more likely to exhibit socially negative behavior regardless of the situation; however, infected women displayed socially negative behavior in situations that did not include an interpersonal aspect. In the Trust Game where there was the possibility of forming reciprocal relationships, infected women were actually more generous compared with uninfected subjects [75]. Recently, a cross-sectional study on 1768 subjects, directly testing the Stress-Coping Hypothesis, found that toxoplasmosis positively associated with levels of stress, so that the infected subjects experienced significantly more stress [26]. In addition, these researchers also found that toxoplasmosis indirectly resulted in higher levels of anxiety through its negative effects on physical health, and also directly via heightened levels of stress. In other words, stress proved to be an important mechanism involved in the pathology of latent toxoplasmosis in this study; hence, it is likely one of the important factors underlying the aforementioned differences between infected men and women.

No single perspective appears sufficient on its own to explain the behavioral modifications observed in *Toxoplasma*-infected individuals. Instead, it is more plausible that evolutionary, physiological, and psychological explanations must be integrated to comprehensively account for the full range of *Toxoplasma*-induced behavioral changes.

For a summary of the findings in this chapter, see Table 2.

## 3. *Toxoplasma* and Sexual Behavior: Key Research Directions

Research into the effects of *Toxoplasma* infection on sexual behavior encompasses three primary areas: (1) alterations in the sexual behavior of infected individuals, (2) changes in how uninfected individuals perceive and interact with infected partners, and (3) parasite-induced modifications of host behavior that exploit neurophysiological pathways associated with sexual arousal to increase predation risk.

*Toxoplasma* infection is found to be associated with sexual promiscuity [76]. One proximate explanation is that this may be linked to deteriorating physical and mental health, leading to a shift toward a fast life history strategy—prioritizing reproduction while health permits [77]. Alternatively, this might result from parasitic manipulation aimed at enhancing the sexual transmission of the parasite. *Toxoplasma* infection has been associated with elevated testosterone levels in male hosts, potentially influencing sexual behavior, see Section 7.2.1. This increase in testosterone may primarily serve as a parasitic strategy to modulate the host’s immune response, facilitating the parasite’s survival.

Parasitic manipulation may enhance the attractiveness of infected individuals to uninfected potential mates, facilitating sexual transmission [78]. Studies have shown that uninfected female rats are more attracted to males infected with *Toxoplasma*. This increased attractiveness could also be a side effect of elevated testosterone levels in infected males, which may enhance secondary sexual traits preferred by females [79].

*Toxoplasma* infection can alter host behavior to increase predation risk, thereby facilitating transmission to feline definitive hosts [45]. Infected rodents exhibit reduced aversion to cat odors, potentially increasing their likelihood of being preyed upon by cats. Evidence suggests that this behavioral shift may be mediated by the parasite’s effects on the amygdala, where stimuli that would normally elicit fear responses become associated with sexual arousal, possibly via dopaminergic and limbic pathways [21].

Within this context, a critical question for interpreting such empirical data concerns the potential role of sexual transmission in the life cycle of *Toxoplasma*.

### 3.1. Sexual Transmission of Toxoplasma

Though rarely classified as a sexually transmitted disease, toxoplasmosis has been shown to spread through sexual contact in some animal species, raising the question of whether *Toxoplasma* may influence sexual behavior in infected males—making them more sexually active or attractive—to increase transmission. Strictly speaking, however, whether sexual transmission actually occurs in humans may be of secondary importance. Because the parasite likely cannot distinguish among its intermediate hosts, the same manipulation strategies—such as elevating testosterone levels— may apply regardless of the host species. Thus, behavioral changes that evolved enhanced transmission via sexual routes in one species may appear in others, even if they do not contribute to the parasite’s spread in that context. Nevertheless, emerging evidence suggests that the sexual transmission of *Toxoplasma* in humans may indeed be possible, as will be discussed in the following section.

The presence of *Toxoplasma* in the semen and testes of various animal species and humans [80,81] suggests the possibility of sexual transmission. This hypothesis is further supported by epidemiological findings, which show that up to two-thirds of pregnant women with toxoplasmosis have no identifiable risk factors [82,83], indicating that additional, less-recognized routes of transmission, such as sexual contact, may exist. Supporting this, *Toxoplasma* seroprevalence has been found to correlate with the prevalence of sexually transmitted diseases across countries [84], hinting at potentially shared modes of transmission. Age-stratified patterns add further weight to this theory, as rising *Toxoplasma* infection rates in women aged 25–35, but not in men [85], suggest a female-specific risk possibly linked to sexual exposure. This is reinforced by studies reporting higher *Toxoplasma* seroprevalence in women with infected male partners [36]. Additionally, *Toxoplasma* infection has been associated with certain forms of schizophrenia [86], and the later onset of symptoms in infected women, compared to infected men and uninfected individuals, further points to a potential risk window in women’s late 20s, possibly reflecting transmission from male partners during stable sexual relationships [87]. The overall decline in *Toxoplasma* seroprevalence in developed countries may also reflect behavioral shifts toward safer sexual practices in the post-AIDS era [84]. Finally, elevated seroprevalence observed among female sex workers [88] lends additional plausibility to the sexual transmission hypothesis.

A related hypothesis suggests oral sex (specifically swallowing contaminated ejaculate) might transmit *Toxoplasma* [89]. An epidemiological study showed that men and women engaging in oral sex (fellatio) exhibit increased *Toxoplasma* seroprevalence compared to control groups [89]. Among adolescents aged 10 to 14 years, there is a notably higher seroprevalence of toxoplasmosis in females compared to males [85]. This age group is characterized by a greater incidence of fellatio relative to penetrative sexual practices. *Toxoplasma* has been detected in human ejaculates [81,90]. An experimental test in mice found no infection after oral administration of ejaculate from *Toxoplasma*-infected men [91], but this result may reflect species-specific differences in susceptibility or transmission pathways. Thus, while *Toxoplasma* appears in human semen, its potential for sexual transmission—particularly via oral routes—remains an open question and subject to further investigation. In this sense, to advance our understanding of the potential for oral transmission of *Toxoplasma* via semen, future research should prioritize realistic and biologically relevant approaches. Humanized mouse models and in vitro assays using human oral or intestinal epithelial cells could more accurately assess mucosal susceptibility, addressing the limitations of previous animal studies. In addition, epidemiological studies should focus on sexually active adolescents and young adults, collecting detailed behavioral data to distinguish sexual from environmental transmission. Partner-discordant studies, where only one partner is seropositive, could provide strong evidence of transmission pathways. Additionally, viability-specific molecular assays (e.g., RNA-based or viability PCR) should be used to confirm the presence of infectious parasites in semen, not just DNA fragments. These approaches would clarify the plausibility of sexual transmission and help guide targeted public health interventions.

### 3.2. The Sexual Behavior of Toxoplasma-Infected Individuals

The influence of latent toxoplasmosis on sexual behavior has been surprisingly studied primarily in humans. Research indicates that many toxoplasmosis-associated behavioral changes may increase the probability of the sexual transmission of the parasite. Alvarado-Esquivel reported increased sexual promiscuity and higher sexual activity in infected men, while, in line with expectations, no such effect was observed in women [76]. The underlying mechanisms remain unclear. One possibility is that infected individuals adopt a fast life-history strategy due to impaired health, prioritizing reproduction while their health permits [77,92]. Another possibility is that this phenomenon represents an adaptive strategy by the parasite to enhance its sexual transmission probability, either in humans or in its natural intermediate hosts. Alternatively, the behavioral changes could be merely side-effects of elevated testosterone levels, which heighten libido. Elevated testosterone levels are well known for their immunosuppressive and immunomodulatory properties [93]. It is therefore plausible that *Toxoplasma* initially evolved to exploit host testosterone to suppress immune responses and improve its own persistence within the host. The accompanying increase in sexual motivation and promiscuous behavior—both linked to higher testosterone—may have arisen secondarily as by-products of this hormonal manipulation. Nevertheless, these behavioral shifts could have been co-opted by the parasite to further enhance its transmission, including through sexual routes where possible. In addition to testosterone, neurobiological changes—including dopamine elevation [74] and modulation of vasopressin and serotonin [21,94]—may heighten sexual urges in *Toxoplasma*-infected individuals. Finally, causality might be reversed—as individuals engaging in riskier sexual behaviors could be simply more likely to become infected [95].

**Figure 4:**
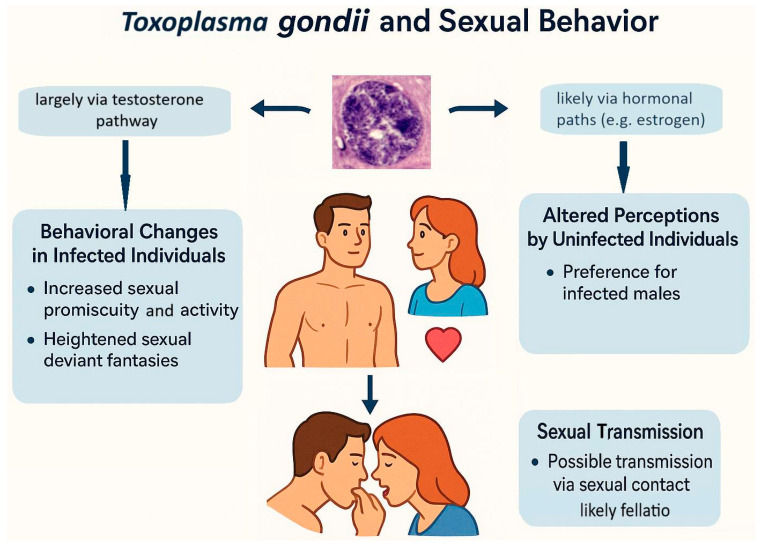
Hypothesized mechanisms linking *Toxoplasma gondii* infection to human sexual behavior and sexual transmission.

*Toxoplasma* seropositivity is also associated with qualitative changes in sexual behavior and altered sexual preferences (see Figure 4). *Toxoplasma* infection has been linked to intensified sexual fantasies involving violence, zoophilia, masochism, and related preferences [95,96]. Despite these increased fantasies, infected men and women generally report lower engagement in such behaviors, possibly reflecting their poorer physical health and reduced energy levels [27,97]. Altered sexual preferences in infected individuals could result from neurobiological modifications primarily intended to increase predation risk—specifically, the transformation of normally fear-inducing stimuli into sexually arousing and therefore rewarding ones [21].

### 3.3. The Sexual Behavior of Uninfected Individuals Towards Toxoplasma-Infected Individuals

Female mate choice commonly involves avoiding infected males, thereby reducing the risk of infection and protecting offspring from potential vulnerabilities. One might therefore predict that uninfected females would reject *Toxoplasma*-infected males. However, there is evidence that *Toxoplasma* can subvert such aversion, potentially manipulating host behavior in ways that facilitate its own transmission.

Female rats detect infection-related odors via specialized neural pathways [98,99]. Typically, high parasite load reduces male attractiveness [100,101]. However, *Toxoplasma* infection can reverse this pattern: infected male rats may become more appealing to females. For example, a study [79] showed that infected males enjoyed greater mating success than uninfected controls, suggesting parasite-induced manipulation favoring infected males’ reproduction and the same phenomenon was observed also in mice [102].

In humans, visual cues play a more significant role than olfactory signals in sexual behavior. Notably, research suggests that *Toxoplasma* may affect men’s physical and behavioral traits associated with attractiveness. According to Flegr et al. [103], infected male students were taller and had lower 2D:4D ratios, indicative of higher levels of testosterone. Hodková et al. [104] found that female observers rated infected men as more dominant and masculine, suggesting that elevated testosterone levels occurring after birth, potentially as a consequence of *Toxoplasma* infection, may play a role. Another study reported reduced facial fluctuating asymmetry in infected men, making them appear healthier [105]. Biochemical data confirm testosterone upregulation in infected men and downregulation in infected women [106]. This influence could occur either directly through the manipulation of these traits or indirectly via physiological changes related to the infection. For example, it remains unclear whether higher testosterone directly facilitates parasite transmission by enhancing attractiveness, or whether it primarily aids *Toxoplasma* in establishing and persisting within the host organism through its immunosuppressive effects [107]. While the previously mentioned studies support the former hypothesis, it is important to recognize that the immunosuppressive effects of toxoplasmosis are extensively documented. Of course, it is plausible that both mechanisms are concurrently at play.

For a summary of the findings in this chapter please, see Table 3.

## 4. *Toxoplasma* and Cognition

Toxoplasmosis has been found to affect cognitive functions in *Toxoplasma*-infected subjects. These effects are suggested to be the result of manipulations that the parasite makes in the infected subject’s CNS to facilitate its transmission from the intermediate host to the definitive host. The *Toxoplasma*-induced dysregulation of dopamine, as a likely important candidate, was first suggested to underlie some of these manipulations by Flegr and his colleagues [74]. Other likely mechanisms such as chronic immune response in the brain, oxidative and inflammatory damage in the brain, neurotransmitter imbalance (e.g., glutamate and GABA), dysregulation of ApoE (the main cholesterol carrier involved in axonal development), and modification of amyloid processing, leading to Aβ immunoreactivity, hyperphosphorylated tau protein, and loss of NMDA receptors have also been suggested to be involved [108]. However, a recent study casts some doubt on the hypothesis that cognitive changes observed in infected individuals result primarily from parasite-driven behavioral manipulation—at least in the case of specific functions such as reaction speed and memory. The study examined the associations between latent toxoplasmosis and cognitive abilities and compared them with those linked to human cytomegalovirus (CMV), another widespread neurotropic pathogen [109]. The results showed that *Toxoplasma* infection was associated with reduced IQ in men, while CMV infection was mainly linked to impairments in memory and reaction speed in women. Since both pathogens can affect the brain but differ markedly in their transmission routes—*Toxoplasma* relies on predation to reach its definitive host, whereas CMV is transmitted primarily through close physical contact—these parallel cognitive effects suggest that at least some of the impairments are likely the result of general pathological changes in the brain rather than specific behavioral manipulations evolved to enhance transmission. In other words, similar cognitive outcomes in infections with very different evolutionary pressures on host behavior argue against a strictly adaptive origin for such effects.

### 4.1. Toxoplasma, Reaction Time, and Processing Speed

Reaction time tests are important tools implemented in psychology and cognitive neuroscience studies and provide invaluable information on information processing speed, temporality and sequence of mental processes, psychomotor functioning, attention, etc. Accordingly, they have been used in a number of studies exploring the association between *Toxoplasma* infection and cognitive functions. One double-blind study in particular found that *Toxoplasma*-infected subjects had significantly longer reaction times than healthy controls in the second and third minutes, but not in the first minute, of a simple reaction time test [110]. This suggests that, as the test progressed, the infected subjects exhibited slower processing speeds. In addition, this study found a positive correlation between the duration of infection and the respondents’ reaction time latency, pointing to the slow and cumulative psychopathogenicity of latent toxoplasmosis. Furthermore, the study reported a progressive decline in psychomotor endurance over the course of the task, with a more pronounced trend among *Toxoplasma*-infected individuals, suggesting that they fatigued more quickly than uninfected participants.

Another study investigating startle reactions and reaction times also found that *Toxoplasma*-infected subjects had longer reaction times. Additionally, the prepulse effect—the influence of a pre-stimulus presented before the main stimulus, which can be either facilitative or inhibitory depending on the study design—was stronger in infected men and correlated with the duration of infection; the longer the infection, the stronger the prepulse effect [111]. The authors suggested that the stronger facilitative effect of the prepulse observed in infected individuals might result from its ability to redirect their attention to the main stimulus during the third minute of the reaction time test—precisely the phase in which their concentration tended to decline most markedly. A systematic review and meta-analysis analyzing 13 studies involving 13,289 participants for a possible association between *Toxoplasma*-infection and cognitive impairment in processing speed (measured by tests such as the Trail Making Test Part A, the Serial Reaction Time Test, or go/no-go reaction time tests) also found a statistically significant negative association between toxoplasmosis and processing speed [112].

It should be emphasized that this association, like many phenomena in biosciences, is more complex, with other important factors likely involved. For example, the RhD phenotype has been observed to moderate the relationship between toxoplasmosis and the risk of traffic accidents. As a study showed in particular, RhD negative subjects with high titers of anti-*Toxoplasma* antibodies were the most adversely affected by the infection apropos the risk of traffic accident, where the RhD positive phenotype actually seemed to play a protective role against the traffic accident risk incurred by latent toxoplasmosis [113]. Two other studies also found that RhD positive *Toxoplasma*-infected men [114] and *Toxoplasma*-infected women [115] had significantly lower sensitivity to *Toxoplasma*-induced reaction time prolongation compared with RhD negative *Toxoplasma*-infected counterparts. Furthermore, both studies reported a negative association between salivary testosterone levels (particularly in men) and reaction time duration. The potential moderating roles of RhD phenotype and genotype and host sex in shaping these effects are discussed in greater detail in Section 8.

### 4.2. Toxoplasma Infection and Cognitive Outcomes

**Figure 5:**
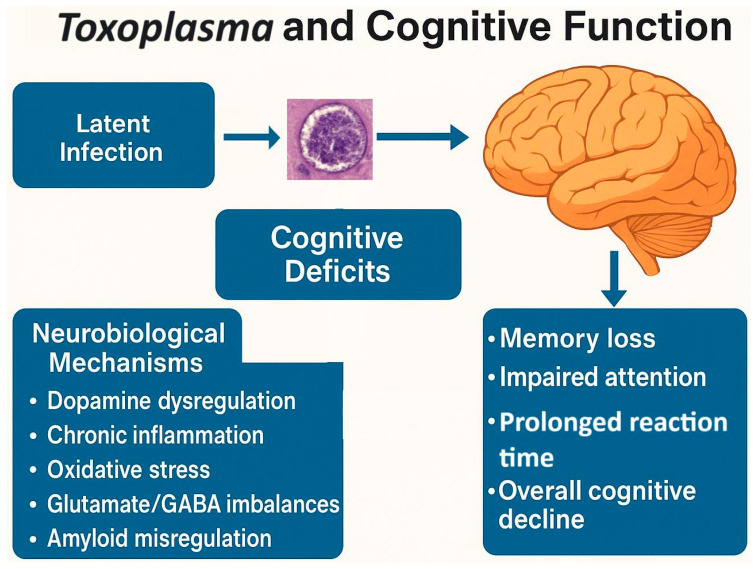
Neurobiological mechanisms linking latent *Toxoplasma gondii* infection to cognitive impairment.

*Toxoplasma*’s ability to affect cognitive functions in infected animals and humans makes toxoplasmosis a valuable model for studying cognitive disorders (see Figure 5). For example, an animal model study demonstrated that infection with this parasite can lead to chronic neuroinflammation through cytokine networks, resulting in impaired learning, memory deficits, and Alzheimer’s disease-like symptoms in infected mice. In addition, infected mice with amyloid-beta 1–42 peptide injected into their hippocampus, as an animal model for Alzheimer’s disease (AD), had more severe learning and memory impairment in comparison with AD model uninfected mice [116]. Another study using non-genetically manipulated mice also found that *Toxoplasma* infection induced beta-amyloid immune reactivity and hyperphosphorylated tau, the two major neurophysiological hallmarks of Alzheimer’s disease (AD). The infected mice exhibited significantly higher neuronal death, loss of N-methyl-D-aspartate receptor (NMDAR) expression, and loss of olfactory sensory neurons. These changes, in turn, led to impaired spatial learning and memory, a failure to recognize social novelty, and reduced olfactory sensitivity in males but not females [117]. Nevertheless, some studies curiously provided evidence that *Toxoplasma*-infection may play a protective role against AD in mice by decreasing the formation of amyloid plaques in the brain [118]. However, it should also be noted that these changes in mice likely reflect a persistent post-acute neuroinflammatory state, as true latency, as seen in infected humans, is rarely observed in the animal model studies. In addition, any protective effects seen may be due to immunomodulation or other factors rather than direct protection from the parasite itself.

Findings in human studies are also controversial in this area of research to a certain degree. Some studies found a significant difference between the anti-*Toxoplasma* IgG antibody titers in AD patients and their sex- and age-matched healthy controls [119]. A recent review also argued that *Toxoplasma*-induced inflammatory reactions and cytokines may somehow reduce amyloid plaque aggregation; however, it remains unknown whether or not these reactions result in long-term cognitive improvement or neurodegeneration [108]. However, some studies on the relations between *Toxoplasma* infection and AD in a sample of AD patients and their age- and sex-matched controls did not find a significant association between AD and infection with *Toxoplasma* [120]. Perry and colleagues [120] offered explanations for the discrepancies between their findings and those of other studies. They cited factors such as their control subjects’ higher socioeconomic status and educational level (presumably protective against Alzheimer’s disease), the higher average age of their participants (which could weaken the association between AD and toxoplasmosis due to higher mortality in the elderly), and the specific strain of the parasite. Importantly, as a study shows, the parasite’s strain seems to be of the essence in this association [121]. Galeh and colleagues, among their other findings, showed that while infection with the PRU strain reduced cognitive impairment in the AD mouse model, infection with the RH strain exacerbated the cognitive functions in these mice. It can also be argued that the effects of *Toxoplasma* infection on cognition are not necessarily confined to Alzheimer’s disease models, as the underlying neuropathophysiological mechanisms of AD may differ from those responsible for the impairment of the same or other cognitive functions reported in numerous other studies. The following section reviews several of these findings.

Studies on older adults show that *Toxoplasma* infection can negatively affect cognition. For example, *Toxoplasma*-infected seniors (65 years old or older) demonstrated a performance reduction in working memory, worse immediate recall and delayed recognition in verbal memory, and deteriorated recall from working memory compared with healthy controls [32]. This study reported no significant difference between the two groups in executive functions. However, another study on adults (40 to 70 years of age) reported poorer executive functioning in infected subjects. Specifically, these subjects had worse reasoning (assessing fluid intelligence through tasks that needed reasoning and logic), worse matrix pattern completion (assessing the ability to specify a missing element in a visual pattern), and worse performance on the Trail Making Test (a numeric task measuring the time required to sequentially complete a path composed of numbers scattered on the page or monitor). This study did not find a significant association between *Toxoplasma*-infection and memory impairment. However, as the authors acknowledged, the dataset they used included few memory tests and hardly any measures of other cognitive functions, such as reaction times and language abilities [122]. In this respect, a recent study on older adults found significant associations between *Toxoplasma* seropositivity and lower scores in immediate memory, delayed memory, the Animal Fluency Test, the Digit Symbol Substitution Test, and global cognition [123].

In younger individuals, a study involving school-aged subjects aged 12 to 16 found that *Toxoplasma* seropositivity was significantly associated with lower reading skills and reduced memory capacity. Interestingly, serum vitamin E levels appeared to moderate this relationship: infected children with lower vitamin E concentrations exhibited more pronounced memory deficits [124]. The moderating role of vitamins is understudied in this line of inquiry; nevertheless, as this study suggested, the association may be due to the role of vitamin E in protecting cells from oxidation and also cytokine production. Studies propose *Toxoplasma*-induced oxidative stress as a likely mechanism in part involved in the pathophysiology of toxoplasmosis-related cognitive impairment. For example, research shows that the oxidative stress associated with *Toxoplasma* infection may play a crucial and distinct role in the mechanisms underlying neurodegeneration and neuropathology associated with *Toxoplasma* infection [125] and its related cognitive impairment [108]. A review article suggested that the adverse effects of *Toxoplasma* infection on cognition may be physiologically rooted in two interrelated mechanisms likely across lifespan; namely the upregulation of dopamine release (the direct effect) and neurodegeneration and impaired dopaminergic activity due to *Toxoplasma*-induced chronic inflammation (the indirect effect) [126]. An animal study specifically demonstrated that *Toxoplasma*-induced cognitive impairment, assessed by novel location (NL), novel object recognition (NOR), Y-maze spatial memory, and nest building tests, was significantly ameliorated after the administration of dimethyl itaconate, an anti-inflammatory drug [127]. Other studies also showed that the administration of haloperidol, which blocks post-synaptic dopamine (D2) receptors, is capable of inhibiting *Toxoplasma* growth in vitro [128].

Patients with schizophrenia or those with bipolar disorder are particularly known to suffer from a variety of cognitive impairments induced by these mental disorders. Accordingly, studies have investigated the role of *Toxoplasma* infection in the cognitive impairment observed in these psychiatric patients. For example, a study of euthymic bipolar patients and controls provided indirect evidence that long-term exposure to inflammation—likely triggered by the *Toxoplasma*-induced synthesis of pro-inflammatory cytokines such as IL-6—may adversely affect cognitive function. Specifically, the Cognitive Deterioration Index was positively correlated with IL-6 mRNA expression levels, but only among *Toxoplasma*-infected individuals. In addition, bipolar patients were significantly more likely to be *Toxoplasma* seropositive than controls in this study [129]. Another study, also investigating the relationship between *Toxoplasma* infection and cognitive impairment in nonpsychiatric controls, bipolar subjects, and patients with schizophrenia, reported a significant association between the anti-*Toxoplasma* IgM (but not IgG) levels and cognitive impairment in the nonpsychiatric controls in RBANS total score, immediate memory, language, and visuospatial/constructional ability. However, the associations between anti-*Toxoplasma* IgM level and both delayed memory and attention impairment were not significant in this group. In contrast, among bipolar patients, higher anti-*Toxoplasma* IgM levels were significantly associated with reduced RBANS total scores, as well as impairments in delayed memory and visuospatial/constructional abilities—but not with deficits in immediate memory, language, or attention. Notably, cognitive functions were more severely affected in the nonpsychiatric control group. This study found no significant association between anti-*Toxoplasma* IgM levels and any cognitive functions in the group of patients with schizophrenia [130]. Nonetheless, while this study investigated anti-*Toxoplasma* IgM, indicating recent infection, the cognitive effects observed may differ in nature and severity from those attributed to chronic or latent toxoplasmosis.

However, other studies reported an association between anti-*Toxoplasma* IgG levels and cognitive decline in patients with schizophrenia, possibly pointing to the role of the duration of infection. For example, a study researching into the effects of digital cognitive training in patients with schizophrenia, found that *Toxoplasma*-seropositive patients had significantly worse verbal memory, social cognition, and global cognition compared with seronegative patients with schizophrenia [131]. In addition, investigating the patients with schizophrenia who had not dropped out of the digital cognitive training program, these researchers reported that the seropositive patients with schizophrenia showed significantly lower z-scores in attention and also trends towards significance for worse verbal memory and learning, working memory, and global cognition compared with seronegative patients with schizophrenia. Another study also reported significantly greater impairments in verbal memory, psychomotor speed, semantic verbal fluency, and literal verbal fluency in *Toxoplasma*-seropositive patients with schizophrenia compared to *Toxoplasma*-seronegative patients with schizophrenia [132]. Nonetheless, it should be noted that this study, unlike Dickerson and colleagues’, did not include subjects with anti-*Toxoplasma* IgM levels indicative of acute infection in its sample. In addition, Dickerson and colleagues [130] acknowledged that the absence of an association between IgM levels and cognitive impairment in patients with schizophrenia—and the weaker association observed in the bipolar group compared to nonpsychiatric controls—could, at least in part, be explained by the anti-*Toxoplasma* effects of antipsychotic medications used in the treatment of both schizophrenia and bipolar disorder.

A large number of studies have reported that dopamine dysregulation, an important factor in the neurophysiology of schizophrenia [34,133] and bipolar disorder [35], likely plays a key role in the pathophysiology of toxoplasmosis (e.g., [74,134], for a detailed discussion, see [24]. As dopamine is an important neurotransmitter involved in a variety of cognitive functions (motor planning, working memory, cognitive flexibility, abstract reasoning, temporal analysis/sequencing, and generativity) [135]—its upregulation or the possible compensatory downregulation of its receptors is likely partly responsible for the cognitive impairments observed in patients with schizophrenia and bipolar disorder, as well as for the additional adverse effects of *Toxoplasma*-infection on these patients’ cognitive functions.

In sum, de Haan et al.’s review and meta-analysis [112], which included 13 studies employing tests such as the Wechsler Adult Intelligence Scale or the Wechsler Intelligence Scale for Children Digit Span, reported a statistically significant association between *Toxoplasma* infection and impaired working memory (SMD = 0.16; 95% CI 0.06–0.26, *p* = 0.002). In addition, this meta-analysis also found a significant association between *Toxoplasma* infection and short-term verbal memory assessed by tests such as the Auditory Verbal Learning Test, the California Verbal Learning Test, or the Verbal Learning and Memory Test (SMD = 0.18; 95% CI 0.09–0.27, *p* = 0.001). This study also revealed a significant association between *Toxoplasma* infection and executive functions measured by the Trail Making Test Part B, verbal fluency tests, or clock drawing tests (SMD = 0.15, 95% CI 0.01–0.28, *p* = 0.03).

Although changes in neurotransmitters due to infection are one of the significant mechanisms involved in these cognitive disorders (see Section 7.1 in the present review article), recent research also indicates that gut microbiota may play a role in this. A study found that toxoplasmosis in mice caused synaptic damage, neuroinflammation, and gut microbiota dysbiosis, leading to colon inflammation and impaired barrier integrity. Notably, eliminating gut microbiota with antibiotics reduced the adverse effects of the parasitic infection on the cognitive function in mice, while fecal microbiota transplantation transferred these impairments to healthy mice. The infection also decreased butyrate-producing bacteria and serum butyrate levels, but dietary butyrate supplementation improved cognitive function. Similar butyrate reductions were observed in humans with high anti-*Toxoplasma* IgG levels, highlighting gut microbiota’s role in *Toxoplasma*-induced cognitive impairment [136]. This points to promising new directions for research into the complex relationship between latent toxoplasmosis and cognitive processes.

### 4.3. Toxoplasma and Intelligence

Several studies have reported an association between *Toxoplasma* infection and intelligence. For instance, a study using Cattell’s factor B from the 16PF test identified a trend of decreasing scores in *Toxoplasma*-infected men over time—the longer the period since diagnosis of acute toxoplasmosis, the lower their scores [20]. A subsequent study using the same questionnaire reported a positive association between *Toxoplasma* infection and intelligence in women [69]. Another study, which utilized the OTIS test of verbal intelligence, found that *Toxoplasma*-infected male military conscripts had lower verbal intelligence scores than their uninfected counterparts [74]. However, a more recent study employing the Cattell’s 16 PF test reported a positive association between *Toxoplasma* seropositivity and intelligence in a large sample of 796 women and 166 men [137]. The most recent study, conducted on a population of 352 women and 205 men using the complex IQ test I-S-T 2000 R, showed that infected subjects, especially men, had significantly lower general intelligence than their non-infected peers [109]. The reduced intelligence in infected individuals was primarily observed in fluid, numeric, and figural intelligence, while verbal intelligence remained unaffected. In this study, intelligence was found to negatively correlate with the level of anti-*Toxoplasma* IgG antibodies, suggesting that either the negative effects diminish over time since infection or that they are stronger in individuals who have experienced a more severe or repeated infection, resulting in higher antibody levels.

RhD phenotype, commonly known as the Rh factor, has also proved to significantly influence the association between toxoplasmosis and intelligence. A study, among its other findings, reported that RhD-positive *Toxoplasma*-infected subjects demonstrated lower verbal and nonverbal intelligence (as measured by the OTIS test and the Wiener Matrizen-Test, respectively) compared to uninfected controls. However, the pattern was reversed for RhD-negative *Toxoplasma*-infected subjects who exhibited higher verbal and nonverbal intelligence scores compared to uninfected controls [72]. Whatever the reason for these differences, the RhD phenotype once again appears to be an important underlying physiological factor in the psychopathophysiology of *Toxoplasma* infection, as can also be seen in Section 8.2.

In summary, the overall picture of the association between *Toxoplasma* infection and intelligence remains less clear compared to other domains of cognition, and definitely in need of further research. This may be due to the complex nature of intelligence itself and the inherent difficulties present in its measurement. For example, performance on intelligence tests is influenced not only by intellectual capacity but also by various psychological traits—such as competitiveness or willingness to cooperate with the researcher—which may themselves be affected by latent *Toxoplasma* infection. Future research would benefit from experimental designs that control for these confounding psychological variables, perhaps by incorporating behavioral assessments or using longitudinal approaches to monitor changes over time. Targeting specific populations, such as individuals with long-standing latent toxoplasmosis, or cohorts from diverse cultural and socioeconomic backgrounds, could help clarify the relationship. Because the cognitive effects of *Toxoplasma* infection may intensify gradually, focusing on individuals with long-term latent infections may increase the likelihood of detecting measurable impacts on intelligence. At the same time, including participants from diverse cultural and socioeconomic contexts can help identify moderating factors, such as education level, environmental stressors, or healthcare access, that might interact with infection status. This approach can also improve the generalizability of findings and shed light on whether the infection’s cognitive consequences are consistent across different populations or shaped by contextual influences.

For a summary of the findings in this chapter, see Table 4.

## 5. *Toxoplasma* Infection and Mental Health

**Figure 6:**
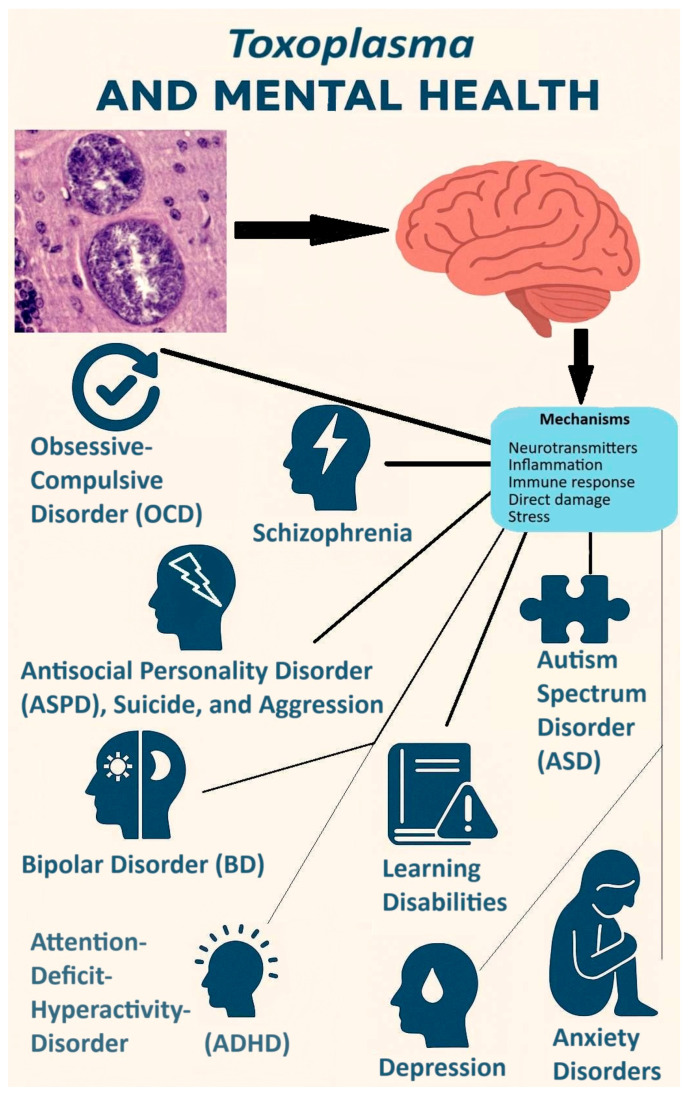
Neuropsychiatric outputs of chronic *Toxoplasma* infection. The width of the lines reflects the strength of the associations.

One of the key areas where the pathology of *Toxoplasma* infection is extensively studied is in the field of mental disorders. As a result of the discoveries made by researchers on the physiological mechanisms employed by *Toxoplasma* in affecting its intermediate hosts, and the overlap of these mechanisms with those involved in the etiology of some of the mental disorders, it has become of immense importance to researchers to study the relationship between the infection and mental disorder in infected humans [138] (see Figure 6). However, we acknowledge that the causes of mental disorders are complex and multifactorial, meaning that no single biological mechanism—and even less so a single neurotransmitter or hormone—can fully explain the onset of a mental disorder.

### 5.1. Schizophrenia

The increased seroprevalence of toxoplasmosis in patients with schizophrenia, as well as the observation that patients with acute toxoplasmosis may exhibit symptoms resembling paranoid schizophrenia, including hallucinations and delusions [139], has been reported in numerous studies since the 1950s. However, it was not until this century that a systematic and long-term investigation into this relationship was undertaken by E.F. Torrey’s team [140].

A meta-analysis of 50 studies examining the relationship between toxoplasmosis and mental disorders, including a total of 71,441 healthy controls and 12,009 *Toxoplasma*-infected subjects, reported an overall significant odds ratio of 1.81 (95% CI 1.51–2.16, *p* = 0.00001) between toxoplasmosis and schizophrenia [141]. Earlier meta-analytical studies, based on older research, suggested an even stronger association between toxoplasmosis and schizophrenia, with overall ORs of 2.73 (95% CI: 2.10–3.60) [28] and 2.71 (95% CI 1.93–3.80) [86].

It has been suggested that increasing concerns regarding patients’ rights might account for this decrease [142]. Contemporary scientific studies can only include patients who voluntarily sign informed consent, which likely means that patients with less severe forms of schizophrenia are more often included. Yet it is precisely *Toxoplasma*-infected patients who tend to suffer from more severe forms of schizophrenia. For example, infected patients have been shown to score higher on both positive and negative symptom scales [87,143] and are more likely to exhibit a continuous rather than episodic form of the disorder [144]. It is therefore not surprising that the proportion of *Toxoplasma*-infected individuals—and hence the odds ratios reported in recent studies—are lower than in earlier decades. Earlier decades saw less emphasis on patients’ rights, leading to studies that included a broader range of hospitalized patients, possibly including those with more severe symptoms or conditions, which could be associated with higher *Toxoplasma* infection rates. In contrast, recent studies, with their increased focus on patients’ rights, might have stricter inclusion criteria, potentially excluding patients who are more severely affected or who have specific conditions, thereby possibly lowering the observed proportion of *Toxoplasma*-infected individuals.

When 174 anonymized clinical samples from schizophrenia patients, rather than participants of a scientific study, were examined, the *Toxoplasma* seroprevalence was 53.2% in males and 29.8% in females [142]. In a sample of 1865 (519 infected) individuals from the Czech population, *Toxoplasma* seroprevalence ranges from 20 to 30% in men and from 24 to 45% in women, depending on age and settlement size [145]. Based on these data, the most likely odds ratio for latent toxoplasmosis in male schizophrenia patients is approximately 3.41, and in female schizophrenia patients it is approximately 0.81. It is also important to note that *Toxoplasma*-infected men are more suspicious and less cooperative, while *Toxoplasma*-infected women are less suspicious and more cooperative than their uninfected counterparts [19,54]. This suggests that a smaller percentage of infected male patients and a larger percentage of infected female patients might be willing to participate in scientific studies. Further evidence supporting a possible link between toxoplasmosis and schizophrenia comes from studies showing that several antipsychotic drugs (e.g., fluphenazine, haloperidol) and the mood stabilizer valproic acid are powerful and specific inhibitors of *Toxoplasma* reproduction [128,146].

Currently, it is widely believed that the association between toxoplasmosis and schizophrenia is mediated by the neurotransmitter dopamine, whose levels are elevated in certain brain regions in individuals affected by either condition. The role of dopamine in this association was first suggested by Flegr et al. [74]. They analyzed the personality profiles of 857 military conscripts using Cloninger’s Temperament and Character Inventory, finding that infected individuals had lower Novelty Seeking scores. Given that dopamine levels are negatively correlated with Novelty Seeking and positively correlated with the risk of positive symptoms in schizophrenia, they hypothesized that the connection between toxoplasmosis and schizophrenia, as well as some behavioral modifications observed in *Toxoplasma*-infected subjects, could be attributed to increased dopamine levels in areas of the brain where local inflammation occurs due to the infection. Later studies, however, showed that the *Toxoplasma* genome contains two genes for tyrosine hydroxylase, a key enzyme in dopamine synthesis [39], and that the tissue cysts of the parasite, along with their surrounding areas, contain large amounts of dopamine [42,147].

### 5.2. Autism

*Toxoplasma* infection has been increasingly implicated in the etiology of autism spectrum disorder (ASD), with several studies suggesting a possible association between *Toxoplasma* infection and the development or exacerbation of ASD symptoms. For example, a large internet cross-sectional study performed on 2377 women (19.7% *Toxoplasma*-seropositive, 0.2% with autism) and 1445 men (11.6% *Toxoplasma*-seropositive, 0.7% with autism) found an odds ratio of 4.78 overall (significant), 1.23 in women (not significant), and 7.83 in men (significant) [148]. Similarly, a study, investigating the seroprevalence of toxoplasmosis in children with ASD, reported a significantly higher presence of anti-*Toxoplasma* IgG antibodies—indicative of past infection—among ASD children compared to healthy controls. Although no significant difference was found in IgM (recent infection), a greater prevalence of *Toxoplasma* infection was observed among ASD children with a family history of the disorder and those from lower socioeconomic backgrounds [149]. Supporting these findings, a meta-analysis confirmed *Toxoplasma* infection as a notable risk factor for autism [150]. Further theoretical integration has been offered by Pavăl (2021), who proposed that various ASD etiologies, including infectious agents like *Toxoplasma*, may converge on a dysfunctional midbrain dopaminergic system. This model emphasizes the mesocorticolimbic and nigrostriatal pathways as central to the behavioral manifestations of ASD [151].

Additional insights into the prenatal aspect of this association come from a large nested case-control study from Finland. This study found that high maternal anti-*Toxoplasma* IgM antibodies were associated with a decreased risk of ASD in offspring, whereas low IgG antibody levels were linked to an increased risk. The authors posited that it may not be the infection itself, but rather the maternal immune response to *Toxoplasma*, that influences the risk of ASD development [152]. In alignment with this perspective, a review proposed a mechanistic model in which the reactivation of latent *Toxoplasma* infection—induced by environmental triggers such as stress, infections, or medication—leads to neuroinflammation, oxidative stress, and enzymatic dysregulation. These processes, in turn, may impair normal neurodevelopment and increase the likelihood of ASD [153]. Nonetheless, caution is warranted. A more recent review, drawing on meta-analytic evidence, suggested that *Toxoplasma* infection might be a risk factor for the development or worsening of autism symptoms, but the reported odds ratio (1.53) did not reach statistical significance (*p* = 0.079), thus leaving room for further clarification [154].

The current body of evidence suggests a complex and multifactorial relationship between *Toxoplasma* infection and ASD. While several studies indicate an association, particularly through markers of past infection and maternal immune responses, others propose possible mechanistic pathways involving neuroinflammation and dopaminergic dysfunction. However, discrepancies in findings—especially regarding the timing of infection, host response variability, and statistical significance—highlight the need for further research. Future investigations would benefit from well-powered longitudinal cohort studies that track maternal infection status and immune markers before and during pregnancy, as well as in early postnatal development. Targeted populations could include high-risk cohorts such as mothers with known *Toxoplasma* exposure or children with early ASD risk markers. Additionally, integrating emerging technologies such as single-cell transcriptomics, neuroimaging, and microbiome profiling could help elucidate cellular and systemic mechanisms linking *Toxoplasma* infection to neurodevelopmental outcomes. Despite growing support for a contributory role of *Toxoplasma*, causality remains unproven, and it is likely that toxoplasmosis acts as one of many interacting environmental factors influencing ASD development. Animal models incorporating humanized immune systems or genetically susceptible backgrounds may offer more insights into causality in this area of research.

### 5.3. Attention Deficit–Hyperactivity Disorder

Researchers in the fields of parasitology and mental health have also investigated the possible association between *Toxoplasma* infection and ADHD in both adults and children. One study exploring this relationship examined 107 children diagnosed with ADHD and 107 ADHD-free children (aged 6–18). The study found no significant difference in anti-*Toxoplasma* IgG antibody titers between the case and control groups. However, a significant positive association was observed between anti-*Toxoplasma* IgG antibody levels and the severity of ADHD [31]. Another study, which examined the association in 45 patients with ADHD and 45 controls, also did not find a significant difference in *Toxoplasma* seropositivity between the two groups. However, it did detect a significant positive association between a maternal history of toxoplasmosis and the subsequent development of ADHD in offspring [155].

In another study, researchers used the ELISA technique to detect anti-*Toxoplasma* antibodies in 117 children and adolescents diagnosed with ADHD, and assessed symptom severity using the Clinical Global Impression–Severity (CGI-S) scale and the Parent ADHD Rating Scale. The results were compared with those of 83 ADHD-free controls. The study found no significant differences in seroprevalence between the two groups and reported no association between *Toxoplasma* infection and ADHD symptoms [156]. To address these conflicting findings, a systematic review and meta-analysis examined the association between *Toxoplasma* infection and ADHD by analyzing data from five cross-sectional and two case-control studies. The authors searched seven major databases (PubMed, ScienceDirect, Scopus, ProQuest, Web of Science, EMBASE, and Google Scholar) without language restrictions and used a random-effects model for the meta-analytic synthesis, which provides more conservative estimates by accounting for between-study variability [157]. The results indicated a non-significant association between anti-*Toxoplasma* antibody titers and an increased risk of ADHD (odds ratio = 2.02; 95% CI 0.97–4.20). Accordingly, toxoplasmosis does not appear to be a significant risk factor for the development of ADHD. Nevertheless, as the authors of the systematic review and meta-analysis noted, further studies are necessary due to the small number of studies on this association (seven studies total, including 3325 participants) and the publication bias observed in their meta-analysis.

Following the publication of this meta-analysis, a large cross-sectional study involving 3698 women (18.7% seropositive, 2.4% ADHD) and 2669 men (9.9% seropositive, 3.0% ADHD) from the general Internet population was conducted [148]. The number of participants in this study was approximately twice that of all the studies included in the meta-analysis combined. The results showed that *Toxoplasma*-infected subjects had a higher prevalence of ADHD than *Toxoplasma*-free individuals (odds ratio = 2.50, 95% CI: 1.58–3.86). The association was much stronger in men (odds ratio = 3.5) than in women (odds ratio = 2.0), but it was significant for both sexes.

In summary, while earlier studies yielded mixed results and meta-analytic findings did not confirm a significant association between *Toxoplasma* infection and ADHD, more recent and better-powered data suggest that such a link may indeed exist. In particular, as noted above, a large cross-sectional study revealed a significantly increased prevalence of ADHD in *Toxoplasma*-infected individuals, with especially strong effects observed in men. These findings underscore the need for prospective, longitudinal cohort studies that track *Toxoplasma* exposure during pregnancy and early childhood, with the follow-up assessments of neurodevelopmental outcomes. Stratifying analyses by sex is essential, given the stronger associations observed in males. Future research should investigate specific biological mechanisms, including chronic low-grade neuroinflammation, alterations in dopaminergic neurotransmission, and sex-dependent immune responses. In animal models, controlled *Toxoplasma* infection studies combined with behavioral testing and neurochemical assays could provide causal and mechanistic insight. Human studies incorporating genetic susceptibility markers, particularly in children with a family history of ADHD, could further clarify *Toxoplasma*’s role as a potential environmental risk factor in ADHD pathogenesis.

### 5.4. Bipolar Disorder

*Toxoplasma* infection has been implicated as a possible risk factor for bipolar disorder (BD). A study exploring this potential association between *Toxoplasma* infection and BD in 110 BD patients and 106 healthy controls found that the *Toxoplasma*-seropositive group had a 2.7-fold odds of having BD compared with the uninfected group (odds ratio = 2.17; 95% CI 1.09–4.36, *p* = 0.028). In addition, levels of IL-6 were significantly higher in the BD patients than the non-patient controls. However, no notable association between the IL-6 transcript levels and *Toxoplasma* status was observed in this study [158]. Another cross-sectional study also reported that 40% of BD patients were also *Toxoplasma*-seropositive compared with the 12% of the uninfected controls. In addition, two biomarkers of oxidative stress; namely 3-nitrotyrosine and 8-hydroxy-2′ deoxyguanosine (796.7 ± 106.28 and 20.31 ± 8.38, respectively) were significantly higher among *Toxoplasma*-positive BD patients compared with *Toxoplasma*-negative BD patients (675.97 ± 144.19 and 7.44 ± 2.86, respectively) and healthy controls (464.02 ± 134.6 and 4.17 ± 1.43, respectively) [29]. Authors suggested that the onset of BD can be partly attributed to *Toxoplasma*-induced oxidative stress in the CNS. Another study examined the effects of 12 neuroleptic compounds on *Toxoplasma* growth in vitro, identifying haloperidol and valproic acid as the most effective inhibitors [128].

A systematic review (13 studies included) and meta-analysis (8 out of the 13 studies included), investigating the association between *Toxoplasma* infection and BD, pointed out that BD patients had a 1.26 (95% CI 1.06–1.47)-fold odds of being also infected with toxoplasmosis when compared with controls [159]. Employing a systematic search of PubMed, PsycINFO, EMBASE and the Cochrane library up to January 10th, 2021, a more recent review and meta-analysis of 23 independent studies with a combined sample of 4021 BD patients and 8669 controls, indicated that BD patients had a larger odd of *Toxoplasma*-seropositivity than controls, both in the random-effects model (odds ratio = 1.69; 95% CI 1.21–2.36) and the fixed-effects model (odds ratio = 1.34; 95% CI 1.19–1.51) [160].

Taken together, while many case-control and biomarker studies report elevated *Toxoplasma* seroprevalence in BD patients, some studies do not replicate these associations. For example, no significant difference in the incidence of BD between *Toxoplasma*-infected and *Toxoplasma*-free individuals was found in a sample of 2377 women (19.7% *Toxoplasma*-seropositive, 3.2% with bipolar disorder) and 1445 men (11.6% *Toxoplasma*-seropositive, 2.8% with bipolar disorder) [148]. In this sample, BD incidence was even higher in uninfected men than in infected men (3.1% vs. 1.2%). The study found an odds ratio of 1.09 overall, 1.43 in women (not significant), and 0.38 in men (not significant). These inconsistencies may partly reflect diagnostic ambiguity: psychotic symptoms in women are more often labeled as affective disorders, including bipolar disorder, rather than schizophrenia—a tendency observed in epidemiological data and clinical cohorts [161,162,163]. This pattern is likely influenced by both sex-specific symptom profiles and concerns about the greater stigma associated with schizophrenia diagnoses [164]. As a result, misclassification may obscure true associations with schizophrenia while artificially inflating those with bipolar disorder. Future studies should carefully address potential diagnostic bias and include stratification by sex. Longitudinal cohort studies with large, demographically diverse samples and rigorous diagnostic reassessment over time are particularly needed. Incorporating standardized psychiatric interviews, alongside serological testing for *Toxoplasma*, could improve reliability. Additionally, use of neuroimaging biomarkers or immunological profiling (e.g., cytokine levels, microglial activation via PET scan) may help clarify mechanisms linking *Toxoplasma* infection to psychiatric outcomes. Finally, genomic or transcriptomic approaches could identify individual susceptibility profiles that moderate infection-related psychiatric risk, providing insight into gene–environment interactions in the context of *Toxoplasma* infection.

### 5.5. Anxiety

Few studies have investigated the role of *Toxoplasma* infection in anxiety and findings are somewhat controversial in this area of research. For example, a study found a positive association between anti-*Toxoplasma* IgG levels and the severity of anxiety in a sample of pregnant women [165]. Another study [166] reported that *Toxoplasma*-seropositive subjects had more than 2 times higher odds of generalized anxiety disorder (GAD) (odds ratio = 2.25; 95% CI 1.11–4.53). Nonetheless, this study reported no significant association between toxoplasmosis and Post Traumatic Stress Disorder (PTSD). Furthermore, another study discovered an association between toxoplasmosis infection and self-directed violence and suicidality [65,167]. An online survey of 3800 participants investigated the associations between toxoplasmosis and 24 mental health disorders, as well as the general indicators of impaired mental health [148]. The results revealed that anxiety was the symptom most strongly associated with toxoplasmosis, exceeding the associations observed for depression and obsessive–compulsive symptoms, and that *Toxoplasma* infection was associated with anxiety disorder. Specifically, the analysis of 2377 women (19.7% *Toxoplasma*-seropositive, 15.7% with general anxiety disorder) and 1445 men (11.6% seropositive, 9.3% with general anxiety disorder) found a significant odds ratio of 1.48 overall and 1.48 in women, while the odds ratio of 1.44 in men did not reach statistical significance. However, another study performed on 1846 participants reported no association between *Toxoplasma* seroprevalence and generalized anxiety disorder (odds ratio = 0.737, 95% CI = 0.218–2.490), and panic disorder (odds ratio = 0.683, 95% CI = 0.206–2.270) [168]. Another study did not either find any relationship between anxiety disorders and toxoplasmosis infection [169]. Nevertheless, a recent study on 8814 individuals, 14.65% of which tested positive for anti-*Toxoplasma* P22 antibody, indicated that *Toxoplasma* seropositivity was associated with an increased risk of developing anxiety disorders (hazard ratios = 1.38; 95% CI: 1.04–1.83) [30].

To summarize, *Toxoplasma* infection is most likely associated with increased anxiety, whether as a transient state or a stable personality trait. However, its association with clinically diagnosed anxiety disorders remains unclear and controversial. This ambiguity may stem from differences in study design, population characteristics, diagnostic criteria, and measurement methods across existing research.

### 5.6. Depression

Studies suggest that the dysregulation of norepinephrine and serotonin is partly implicated in the etiology of depression; with inflammation proposed as one of the mechanisms contributing to altered neurotransmitter levels, extensive reviews on this topic are provided by Dionisie et al. [170]. It is of importance that the pathophysiology of *Toxoplasma* infection is also implicated to be partly caused by disturbances in the synthesis and levels of these two neurotransmitters and inflammation—discussed later in this article (Section 7.1). Accordingly, researchers have tried to investigate the possible associations between *Toxoplasma* infection and depression.

Some studies reported a relationship between *Toxoplasma* infection and depression in the infected individuals. For example, an early case study described a patient suffering from depression who exhibited a limited reaction to antidepressant medications. Serological testing later revealed *Toxoplasma* infection, and a marked improvement in the patient’s response to treatment was observed only after the parasitic infection was appropriately addressed [171]. Some more recent studies have also found an association between *Toxoplasma* infection and depression [172]. In this respect, an online study on the association between *Toxoplasma* infection and mental disorder, employing the 71-item abbreviated form of MMPI, revealed that the length of time since infection could predict the male participants’ depression scores when analyzed using a robust regression model. Nevertheless, their scores were not in the range indicative of psychopathology [173].

More importantly, a meta-analysis study did not find any significant association between *Toxoplasma* infection and major depression (MD), and its results revealed a non-significant overall odds ratio of 1.21 (95% CI 0.86–1.70, *p* = 0.28) [141]. A large internet cross-sectional study performed on 3698 women (18.7% *Toxoplasma*-seropositive, 10.4% MD+) and 2669 men (9.9% *Toxoplasma*-seropositive, 6.4% MD+) showed no association between toxoplasmosis and diagnosed major depression (odds ratio = 1.13) neither in men (odds ratio = 1.02) nor women (odds ratio = 1.14) [148]. Notably, this study was published after the aforementioned meta-analysis and was therefore not included in it. However, the same study reported a significant correlation between *Toxoplasma* seropositivity and the self-reported intensity of depressive symptoms, suggesting that while *Toxoplasma* infection may not increase the incidence of major depression, it could influence mood-related experiences or subclinical affective states.

In conclusion, although the possibility of a link between *Toxoplasma* infection and depression has been explored in several studies, the overall evidence does not strongly support a causal relationship. While isolated findings—including case reports and smaller observational studies—have suggested an association, larger cross-sectional studies and meta-analyses have not confirmed a significant link with clinically diagnosed major depression. The observed inconsistencies may reflect methodological limitations, confounding factors, or a failure to clearly distinguish between transient depressive symptoms and major depressive disorder. Taken together, current research suggests that *Toxoplasma* infection is unlikely to be a major contributing factor to clinically defined depression.

### 5.7. Obsessive–Compulsive Disorder

Obsessive–compulsive disorder (OCD) is another mental disorder for which the association of which with *Toxoplasma* infection has been explored. For example, a study investigating *Toxoplasma*-seropositivity in OCD patients found that the rate of infection with *Toxoplasma* was significantly higher in OCD patients (47.62%) compared to healthy volunteers (19%) [174]. Another research, exploring the association between *Toxoplasma* infection and OCD in a large sample of participants (n = 7471) using the standard Obsessive–Compulsive Inventory-Revised (OCI-R), among other findings, reported that the infected subjects had about a 2.5 times higher odds of OCD. In addition, the infected subjects, even those without OCD, scored higher on the OCI-R in this study [175]. The previously mentioned cross-sectional online study [148] conducted on 2377 women (19.7% *Toxoplasma*-seropositive, 4.0% with OCD) and 1445 men (11.6% *Toxoplasma*-seropositive, 5.8% with OCD) found a higher prevalence of OCD in *Toxoplasma*-infected individuals compared to uninfected ones (an odds ratio of 1.86 (significant) overall, 1.80 (significant) in women, and 1.94 (significant) in men). In addition to clinical diagnoses, infected individuals of both sexes also reported significantly more problems with obsessions on self-report scales (*p* < 0.0005).

A case-control study [176] looking into the association between *Toxoplasma* infection and OCD in 90 OCD patients and 90 controls, assessing the IgG and IgM anti-*Toxoplasma* antibodies and presence of *Toxoplasma* in the blood of the participants using ELISA and PCR methods, respectively, also indicated that the levels of IgG anti-*Toxoplasma* antibodies were significantly associated with a higher risk of OCD in *Toxoplasma*-infected subjects (odds ratio = 3.71; 95% CI 1.88–7.30, *p* = 0.001). In addition, in patients with OCD, these antibodies levels were strongly linked with a higher risk of resistance to treatment (odds ratio = 3.81; 95% CI 1.42–10.17, *p* = 0.008).

In addition, A meta-analysis of the relationship between *Toxoplasma* infection and OCD, investigating 12 published articles (9 case-control and 3 cross-sectional studies) with no restriction of language and an overall sample size of 9873, using the inverse variance method and the random effect model, also revealed a significant common odds ratio of 1.96 (95% CI 1.32–2.90) [177]. In total, 389 subjects had OCD (25.96% *Toxoplasma*-infected) and 9484 were OCD-free (17.12% *Toxoplasma*-infected). Finally, an ecological study of 88 countries also revealed an association between *Toxoplasma* infection and OCD, in total (B = 0.836, *p* = 0.001), for European countries (B = 0.681, *p* = 0.018), and for non-European countries (B = 0.925, *p* = 0.001) [27].

In conclusion, the association between *Toxoplasma* infection and OCD is supported by the consistent findings across case-control, cross-sectional, ecological studies, and a meta-analysis. The relationship has been confirmed in both sexes, using clinical diagnoses and self-report data. Together, these results strongly suggest a genuine link between *Toxoplasma* infection and OCD.

### 5.8. Antisocial Personality Disorder

Antisocial personality disorder (ASPD) is defined by the Diagnostic and Statistical Manual of Mental Disorders, Fifth Edition, Text Revision (DSM-5-TR) [178] as a pervasive pattern of disregard for, and violation of, the rights of others occurring since the age of 15 years. This pattern is indicated by at least three of the following: failure to conform to social norms with respect to lawful behaviors, deceitfulness, impulsivity, irritability and aggressiveness, reckless disregard for safety, consistent irresponsibility, and lack of remorse. The individual must be at least 18 years old, with evidence of Conduct Disorder with onset before age 15 [178]. There are data indicating that latent *Toxoplasma* infection might influence the traits related to aggression, impulse control, and rule-breaking, thereby having a potential link to antisocial or aggressive behavioral outcomes. However, direct studies focusing specifically on ASPD diagnosis are scarce. As far as we know, only one large-scale cross-sectional study to date has explicitly examined *Toxoplasma* seropositivity in clinically diagnosed ASPD individuals as a distinct group [148]. This already mentioned Internet study performed on 2377 women (19.7% *Toxoplasma*-seropositive, 3.2% with antisocial personality disorder) and 1445 men (11.6% *Toxoplasma*-seropositive, 3.9% with antisocial personality disorder) found an odds ratio of 1.63 overall (significant), 1.39 in women (non-significant), and 2.26 in men (significant). In this study, the participants self-reported their diagnosed psychiatric disorders, and it is likely that some patients, specifically those with antisocial personality disorder, were reluctant to report this diagnosis.

Beyond diagnosed disorders, latent *Toxoplasma* infection has been linked to personality traits associated with antisocial behavior. A recent study of 150 healthy university students in Turkey assessed participants for impulsive and antisocial behavioral outcomes as well as the Big Five personality traits [179]. The seroprevalence in this young adult sample was 30.7%, reflecting the local infection rate. *Toxoplasma*-positive students were significantly more likely to report a history of criminal behavior (having at least one criminal conviction) compared to seronegative students. They also had higher rates of non-suicidal self-injury and suicide attempts, pointing to greater impulsive dyscontrol in the infected group. In terms of personality profiles, infected individuals showed lower Agreeableness and Conscientiousness and higher Neuroticism on the Big Five inventory, with no differences in Extraversion or Openness. Lower agreeableness and conscientiousness are noteworthy because they indicate a more hostile, less cooperative disposition and poorer impulse control/discipline—traits highly germane to ASPD (which is characterized by antagonism and disinhibition in trait models). The fact that these personality differences were highly correlated with anti-*Toxoplasma* IgG levels supports the idea that the infection might subtly shift personality in a direction that could foster antisocial tendencies (at least in men; see sex differences below).

These findings align with earlier observations by Flegr and colleagues. In a series of studies from the Czech Republic (using Cattell’s 16 Personality Factor questionnaire on >1800 subjects), *Toxoplasma*-infected men consistently scored lower on superego strength (a factor reflecting rule consciousness and moral fiber) and higher on vigilance (suspiciousness), compared to uninfected men [180]. Infected males were more likely to disregard rules and social norms, being described as more expedient and less conscientious. In contrast, infected women in those studies showed an opposite trend (if anything, higher conscientiousness than uninfected women). This intriguing sex-specific effect—wherein *Toxoplasma*-infected men exhibit a more antisocial personality profile (less rule-abiding, more distrustful) while *Toxoplasma*-infected women do not—has been documented in multiple independent samples, as can also be seen in Section 8.1. It suggests that any *Toxoplasma* infection-related risk for antisocial behavior may be modulated by sex.

In a recent cross-sectional study performed on 7762 members of the internet population, the correlation between toxoplasmosis and Big Five traits as well as Dark Triad traits was examined [137]. Again, a negative correlation with Conscientiousness emerged. Surprisingly, *Toxoplasma* infection correlated negatively with Dark Triad traits, with significant negative correlations observed for Machiavellianism in both sexes and Narcissism in women.

Aggression and violent behavior have repeatedly been shown to be positively associated with *Toxoplasma* infection. For instance, the study by Coccaro et al. [56] investigated *Toxoplasma*-seropositivity in individuals with Intermittent Explosive Disorder (IED)—a DSM-5 disorder of recurrent, impulsive aggression. This case-control study of 358 adults employed clear diagnostic categories and validated psychometric instruments to quantify aggression and impulsivity. The results showed a significant association between latent *Toxoplasma* infection and aggressive behavior. Specifically, *Toxoplasma* seropositivity rates were 21.8% in the IED group, and 9.1% in healthy controls. Infected individuals had higher scores on the Buss–Perry Aggression Questionnaire and other anger/impulsivity scales than uninfected individuals (infection was associated with elevated trait aggression, *p* = 0.022, and impulsivity, *p* = 0.05). However, when aggression and impulsivity scores were entered together in analyses, only the aggression link remained significant (seropositive subjects had markedly higher aggression scores, *p* = 0.011). Importantly, this association was not explained by other comorbid disorders or demographic factors; the authors controlled for the presence of other personality disorders and mood/anxiety diagnoses, and the *Toxoplasma*–aggression link persisted.

An ecological study based on WHO surveillance data monitoring the incidence of diseases and health outcomes examined the correlation between *Toxoplasma*-seroprevalence and both mortality and Disability-Adjusted Life Years (DALYs) due to violence in 88 countries [27]. The results indicated positive correlations with both mortality (τ = 0.218, *p* = 0.003) and DALYs (τ = 0.244, *p* = 0.0008). These correlations were significant only in the 55 non-European countries; in the 29 European countries, the correlations were non-significant and negative, suggesting that in Europe, a distinct contextual or epidemiological factor may be responsible for the observed levels of violence, potentially obscuring any effects of *Toxoplasma* infection.

However, not all studies have found a significant association between *Toxoplasma* infection and violence. Rocha-Salais et al. [181] conducted a cross-sectional survey of N = 128 prison inmates to test whether *Toxoplasma* infection was associated with violent criminal behavior. Violence was assessed via three independent methods: a structured risk assessment (HCR-20), criminal records (type of crime committed), and a psychometric aggression inventory (Buss–Perry Aggression Questionnaire). The results indicated no significant association between the infection and inmates’ history of violence or aggression levels. However, the study had limited statistical power due to a relatively small sample size and a low *Toxoplasma* IgG seroprevalence (on the order of only a few percent).

To summarize, direct evidence connecting latent *Toxoplasma* infection to antisocial personality disorder as a diagnosed condition is limited, and no definitive causal relationship has been established. However, several studies in related domains suggest meaningful associations. As a common, modifiable exposure, *Toxoplasma* infection could represent a novel risk factor in the etiology of certain antisocial behaviors. Longitudinal and mechanistic studies (in both general and forensic populations) are needed to determine whether this parasite truly contributes to the development of antisocial personality disorder or related behavioral syndromes.

### 5.9. Learning Disabilities

As detailed in Section 4, *Toxoplasma* infected individuals exhibit impaired cognitive performance in multiple domains. However, only two cross-sectional studies have so far investigated the association between *Toxoplasma* infection and diagnosed learning disabilities (LDs). Both studies relied exclusively on self-reported data regarding both serologically confirmed *Toxoplasma* infection and professionally diagnosed learning disabilities, yet both included negative control by examining a wide panel of mental disorders, of which only a subset correlated with *Toxoplasma* infection.

An earlier study examined a panel of 20 psychiatric disorders in a sample of 1332 individuals, of whom 415 were previously diagnosed as *Toxoplasma*-seropositive, and 43 had been diagnosed with a learning disability [175]. The results showed a 2.57-fold higher likelihood of LDs among *Toxoplasma*-seropositive individuals than among their seronegative counterparts. This study provides statistical evidence of an association between *Toxoplasma* infection and learning disabilities, though it is observational and not causal. A more recent Internet-based study conducted on 2377 women (19.7% *Toxoplasma*-seropositive, 4.4% with learning disabilities) and 1445 men (11.6% *Toxoplasma*-seropositive, 5.12% with learning disabilities), controlling for confounders such as age, sex, and urbanization level, reported an odds ratio of 1.59 overall (significant), 1.29 in women (non-significant), and 2.29 in men (significant) [148]. This association remained significant even after correcting for multiple comparisons using the Benjamini–Hochberg procedure. The authors suggested that dopaminergic and glutamatergic dysregulation, possibly triggered by the infection, might contribute to neurodevelopmental and cognitive impairments, hinting that *Toxoplasma*’s interference with brain signaling and immune response pathways may play a contributing role.

In summary, the current section and Section 4 reviewed the evidence linking *Toxoplasma* infection to diagnosed learning disabilities. To our knowledge (as of April 2025), only two empirical studies have directly examined this association, and both indicate that such a link may indeed exist. Nevertheless, numerous studies have shown that *Toxoplasma* infection is frequently associated with mild cognitive inefficiencies. Given the high global prevalence of the infection, even modest cognitive impairments at the population level may be of public health importance. New cross-sectional and, if possible, longitudinal studies (e.g., cohort studies tracking seroconversion and cognitive change) are needed to clarify causality and to uncover the potential mechanisms through which this common parasite might contribute to learning or memory difficulties.

### 5.10. Substance Use Disorder

*Toxoplasma gondii* infection has been proposed to affect addiction because the parasite encodes tyrosine-hydroxylase-like enzymes that can boost host dopamine signaling, a pathway targeted by most commonly abused drugs [39,74]. Epidemiological evidence, however, is inconsistent.

A large NHANES analysis of U.S. adults reported reduced odds of ever using tobacco (odds ratio = 0.87, *p* = 0.044), marijuana (odds ratio = 0.60, *p* = 0.001), cocaine (odds ratio = 0.72, *p* = 0.006), heroin (odds ratio = 0.60, *p* = 0.005), or methamphetamine (odds ratio = 0.54, *p* = 0.001) in seropositive individuals [182]. Conversely, smaller case-control studies have found higher seroprevalence among drug users. For example, an Egyptian study performed on 440 subjects, examined by enzyme multiplied immunoassay for commonly abused substances in Egypt (tramadol, cannabis, opiates, barbiturates, and benzodiazepines), showed that toxoplasmosis seroprevalence was 1.98 times higher in 350 abusers than in 94 controls (*p* < 0.0001) [183]. For cigarette smoking, an Iranian study observed the opposite pattern—seropositivity was less common in 216 active smokers (OR ≈ 0.35, *p* < 0.001) than in 324 matched non-smokers [184].

A large cross-sectional study conducted on data from 2619 non-clinical subjects found a non-significant positive association between *Toxoplasma* seropositivity and drug addictive disorder in men (odds ratio = 3.29, *p* = 0.01) and a negative trend for women (odds ratio = 0.51, *p* = 0.32) [185]. A similar cross-sectional study performed on 1256 individuals showed a significant negative association between seropositivity and alcohol use disorder—all 12 cases with the diagnosis were among 963 *Toxoplasma*-seronegative individuals, with none among 293 *Toxoplasma*-seropositive individuals (*p* = 0.003) [185]. A limitation of both studies was their reliance on self-reported data, suggesting that the results might reflect a willingness to report this sensitive disorder rather than its true incidence in the population.

An ecological study found a positive correlation between the *Toxoplasma* seroprevalence and alcohol use disorder-related burden, explaining 13% of the between-country variability (*p* = 0.07) in European countries but a negative correlation explaining 5% variability (*p* = 0.1) in non-European countries [27].

A meta-analytic study published in 2015 identified four case-control studies enabling the comparison of drug abuse incidence (mostly heroin addiction) in *Toxoplasma*-seropositive and seronegative individuals. The overall odds ratio in this case was 1.91 (*p* < 0.0001) [185].

Taken together, current results do not support a simple causal link between *Toxoplasma* infection and addiction; instead, the association appears to vary with host demographics, neuropsychiatric comorbidity, and study design. Mechanistic pathways—dopaminergic modulation, neuroinflammation, and heightened risk-taking—remain biologically plausible but require rigorous longitudinal and experimental studies to clarify their relevance.

### 5.11. Neurotropic Zoonoses: Synergistic and Antagonistic Effects of Toxoplasma, Bartonella, and Borrelia Infections on Mental Health

Understanding the associations between *Toxoplasma* infection and psychiatric disorders can also be enhanced by insights from research on cat-scratch disease, an infection caused by the bacterium *Bartonella henselae*. The bacteria are present in flea feces and are transmitted from cats to humans through scratches contaminated with infected flea excrement. Research has shown that *B. henselae* is linked to various psychiatric disorders, manifesting as both neurological and neurocognitive symptoms [186].

A recent study has highlighted a correlation between participant-reported *Bartonella*-associated cutaneous lesions (BACL) and psychiatric symptoms such as anxiety, depression, and schizotypy. In this study, participants conducted self-inspections of their skin and compared their observed lesions to a reference set of images depicting different types of infectious lesions, including those caused by *B. henselae* [187]. Another recent study revealed that a significantly greater proportion of adults diagnosed with psychosis exhibited the presence of *Bartonella* spp. DNA in their bloodstream compared to adult controls without psychosis. However, this study did not identify any significant differences in seroreactivity to *Bartonella* spp. using immunofluorescence assays (IFAs) between adults with psychosis and those unaffected by the disorder [188]. Conversely, other research has indicated no specific association between *B. henselae* and cognitive impairment [189] or psychiatric conditions such as schizophrenia and schizoaffective disorder [190].

The findings presented are somewhat contentious, indicating that the interactions between various zoonotic diseases that can be transmitted from cats to humans, and additional influencing factors may be at work. In this context, a case-control study involving 250 subjects matched for *Toxoplasma* infection status and age found that *Bartonella* seropositivity was positively associated with Beck Depression Inventory scores exclusively in *Toxoplasma*-seronegative men, while it exhibited a negative correlation with health outcomes in *Toxoplasma*-seronegative women [191]. Furthermore, *Bartonella* seropositivity appeared to confer protective effects against the increased neuroticism associated with *Toxoplasma* seropositivity in men and *Toxoplasma* seropositivity seemed to offer protective benefits against health issues linked to *Bartonella* seropositivity in women. The comparative analysis of the associations between mental and physical health problems and *Bartonella* seropositivity, alongside reported cat-related injuries, suggested that distinct factors may be at play. The authors argue that it is plausible that infections caused by different pathogens transmitted through cat-related injuries—other than *B. henselae*—may be responsible for the observed correlations between such injuries and symptoms of depressiveness as well as diagnosis of major depression. A cross-sectional study performed on 5500 subjects showed a strong association between sustained cat scratching injury and being diagnosed with major depression as well as a high Beck Depression Inventory score [192]. Cat scratching is often associated with cat biting injury, and this association may help explain the results of an earlier case-control study performed on 1.3 million patients of the University of Michigan Health System. This study reported that 47% of women and 24% of men who were treated in hospital due to a cat bite were diagnosed with depression (mostly ICD 311, ICD 296.30, ICD 296.20), while the overall rate of diagnosed depression in the same hospital population was only 9% [193].

Analogous findings were observed in the case of infection with the tick-borne spirochete *Borrelia burgdorferi* [138]. In a sample of 3252 women (23.0% previously diagnosed with borreliosis) and 3865 men (19.8% previously diagnosed with borreliosis), only three out of 22 examined conditions showed significant correlations with seropositivity: major depression (odds ratio = 1.65, 95% CI: 1.14–2.38, *p* = 0.008), posttraumatic stress disorder (odds ratio = 1.64, 95% CI: 1.01–2.65, *p* = 0.045), and other mental disorders (odds ratio = 1.66, 95% CI: 1.08–2.56, *p* = 0.021). By comparison, *Toxoplasma* seropositivity was associated with a larger number of psychiatric disorders—seven significant associations, and two additional disorders with strong trends toward significance. Most effects of borreliosis were more pronounced—or exclusively present—in individuals who were also seropositive for *Toxoplasma* infection.

In conclusion, the observed interactions between toxoplasmosis, *Bartonella* or *Borrelia* infection, and mental health are complex and often modulated by sex and co-infection status. The findings suggest that neither infection should be considered in isolation when assessing the psychiatric outcomes associated with cat-related exposure. While *Bartonella* and *Borrelia* seropositivity alone has shown inconsistent associations with mental disorders, its interaction with *Toxoplasma* serostatus reveals potentially antagonistic or modifying effects on depression and neuroticism. These data highlight the need for more subtle, multivariable approaches that account for co-infections and sex-specific responses in the study of zoonotic infections and mental health.

For a summary of the findings in this chapter, see Table 5.

## 6. *Toxoplasma* and Physical Health

**Figure 7:**
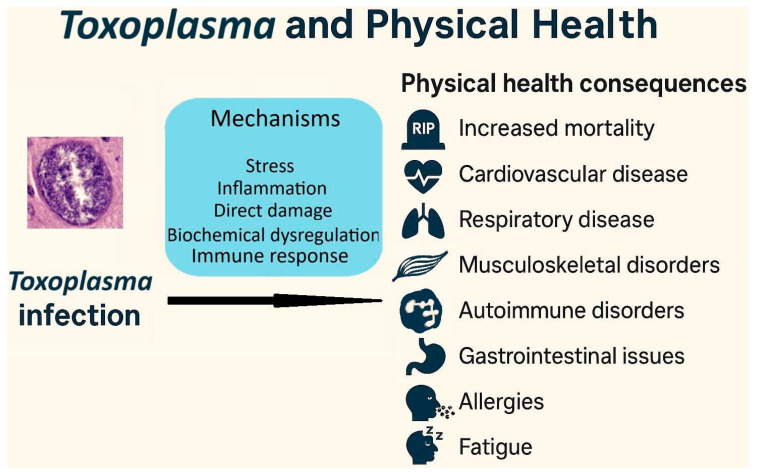
Chronic *Toxoplasma* infection effects on physical health.

While *Toxoplasma* has often been studied in the context of behavioral and neurocognitive changes, its potential impact on physical health may be of even greater importance—particularly from a clinical and public health perspective (see Figure 7). Given the high global prevalence of this lifelong infection, any evidence suggesting that *Toxoplasma* contributes to deterioration in physical or general mental health would have significant implications for medical practice. Indeed, one of the most commonly proposed explanations for the observed differences in behavior- or cognition-related variables between infected and uninfected individuals is that these differences result from subtle, nonspecific health deterioration caused by the infection. Although this side-effect hypothesis may be less conceptually provocative than the manipulation hypothesis, it raises critical questions about the broader somatic consequences of latent toxoplasmosis.

### 6.1. Health Burden of Toxoplasma Infection

As previously mentioned, toxoplasmosis was once considered to be of little or no medical concern in immunocompetent individuals; however, studies over the past decade have suggested otherwise. While the effects of acute toxoplasmosis on physical health have been relatively well studied (for example, see [194]), the health effects of chronic *Toxoplasma* infection have been systematically investigated far less frequently.

A comprehensive ecological study not only provided an extensive review of previously published studies on the various effects of *Toxoplasma* infection on physical health but primarily focused on examining correlations between *Toxoplasma* infection seroprevalence and disease burden using WHO mortality and morbidity data for 129 diseases [27]. The study collected and analyzed seroprevalence data from 88 countries published between 1995 and 2008, focusing on women of childbearing age—a relatively homogeneous demographic group for which the most comprehensive and comparable international data are available. Statistical models included temperature, humidity, and GDP per capita as covariates. The findings revealed that *Toxoplasma* seroprevalence was correlated with morbidity, measured as Disability-Adjusted Life Years (DALYs), in 23 of 128 diseases and disease categories on the WHO list (18 positively and 5 negatively), with an additional 12 diseases showing similar trends (*p* < 0.1, all positive). The *Toxoplasma* seroprevalence was correlated with mortality in 12 diseases (11 positively and 1 negatively).

*Toxoplasma* seroprevalence was positively correlated with morbidity for conditions such as chlamydia, gonorrhea, pertussis, diphtheria, non-communicable diseases, prematurity and low birth weight, prostate cancer, other neoplasms, endocrine disorders, epilepsy, OCD, glaucoma, cardiovascular diseases, asthma, periodontal disease, benign prostatic hypertrophy, and congenital anomalies. Conversely, it was negatively associated with trichuriasis, hookworm disease, liver cancer, posttraumatic stress disorder, and Alzheimer’s and other dementias—likely because individuals severely affected by *Toxoplasma* infection-related health issues may die earlier in life and thus never reach the age when neurodegenerative diseases such as Alzheimer’s typically manifest. Some associations were particularly strong—for instance, differences in seroprevalence explained 23% of the variation in total disease burden and 31% of the variation in cardiovascular disease mortality across 28 European countries.

Significant and widespread effects of *Toxoplasma* infection on physical health were confirmed by a large cross-sectional study involving 333 *Toxoplasma*-infected and 1486 *Toxoplasma*-free subjects, who completed an extensive online questionnaire covering a wide range of physical and mental health-related topics [97]. The results showed that *Toxoplasma*-infected individuals scored significantly worse on 28 out of 29 health-related variables. For example, these individuals reported using more antibiotics in the past year, being hospitalized more frequently in the past five years, visiting more medical specialists in the past two years, currently taking more prescription medications, currently taking more non-prescription medications and health supplements, feeling drowsy more often, feeling more fatigued, and experiencing headaches more frequently. *Toxoplasma* infection was most strongly associated with musculoskeletal disorders (τ = 0.107, *p* = 0.0005), followed by neurological (τ = 0.088, *p* = 0.0005), immune (τ = 0.085, *p* = 0.0005), metabolic (τ = 0.079, *p* = 0.0005), respiratory (τ = 0.068, *p* = 0.0001), allergic (τ = 0.053, *p* = 0.004), digestive system (τ = 0.052, *p* = 0.004), and mental health disorders (τ = 0.050, *p* = 0.008).

Participants were also asked to indicate from a list of 134 diseases which conditions they had been diagnosed with in the past. For 77 of these diseases, the incidence was significantly higher in *Toxoplasma*-infected individuals compared to *Toxoplasma*-free individuals. Strong associations were observed, for example, with bronchitis (odds ratio = 1.44, *p* = 0.0005) (but not with pharyngitis), acquired immunodeficiencies except AIDS (odds ratio = 3.73, *p* = 0.0005), both diarrhea (odds ratio = 1.19, *p* = 0.014) and constipation (odds ratio = 1.32, *p* = 0.005), swollen lymph nodes (odds ratio = 3.76, *p* = 0.0005), mononucleosis (odds ratio = 1.63, *p* = 0.0005), rheumatoid arthritis (odds ratio = 2.95, *p* = 0.012), weight loss (odds ratio = 2.42, *p* = 0.0005), food allergies (odds ratio = 1.35, *p* = 0.006), respiratory allergies (odds ratio = 1.24, *p* = 0.006), autoimmunity (odds ratio = 1.76, *p* = 0.001), immunodeficiency (odds ratio = 2.05, *p* = 0.0005), coeliac disease (odds ratio = 3.43, *p* = 0.0005), gall bladder attack (odds ratio = 2.12, *p* = 0.0005), recurrent abortion (odds ratio = 2.07, *p* = 0.031), hypothyroidism (odds ratio = 2.04, *p* = 0.0005), tics (odds ratio = 2.64, *p* = 0.0005), fasciculation (odds ratio = 1.59, *p* = 0.002), learning disabilities (odds ratio = 2.48, *p* = 0.0005), autism (odds ratio = 1.82, *p* = 0.008), osteoporosis (odds ratio = 2.27, *p* = 0.010), scoliosis (odds ratio = 1.75, *p* = 0.0005), epilepsy (odds ratio = 3.37, *p* = 0.001), and asthma (odds ratio = 1.71, *p* = 0.0005). Although the associations are observed in seropositive adults, and thus are unlikely to reflect congenital infection, the potential contribution of subclinical congenital or reactivation events cannot be entirely excluded in individual cases. However, in the vast majority of immunocompetent individuals and those not infected congenitally, this possibility remains highly unlikely.

The third study was conducted on 39 *Toxoplasma*-infected and 40 *Toxoplasma*-free female students [195]. The primary goal of this case-control study was to refute the possibility that participants report deteriorated health only after learning that they are infected with *Toxoplasma*. The participants completed two identical questionnaires (an anamnestic questionnaire evaluating physical health status, and a standard N-70 questionnaire assessing anxiety, depression, obsession, hysteria, hypochondria, psychosomatic symptoms of vegetative lability, and psychasthenia) before and three years after being informed about their toxoplasmosis status. The results showed that *Toxoplasma*-infected subjects had more frequent and serious physical health problems than uninfected subjects not only after but also before they were informed of their *Toxoplasma* infection. Infected students spent more time in the hospital over the past five years and estimated their life expectancy to be lower than that of uninfected subjects.

A secondary finding of this case-control study revealed that, in addition to its modifying effect on the impact of toxoplasmosis on cognitive performance, the RhD phenotype also modifies its effect on health status. It was found that the poorer health condition of infected individuals was primarily seen in RhD-negative subjects. These individuals had significantly more frequent neurological problems, depression, other psychological issues, fatigue, hospitalizations, and shorter estimated life expectancy. Among the RhD-positive individuals infected with *Toxoplasma*—who were twice as numerous—only the frequency of hospitalizations and life expectancy estimates were significantly worse compared to uninfected subjects. In some variables (such as the frequency of chronic pain and other psychiatric issues), infected women even exhibited better health than uninfected individuals.

The last two studies directly tested the side-effect hypothesis, aiming to determine whether changes in personality are merely side effects of deteriorating physical or mental health in infected individuals. In the first study, 7762 members of an Internet population completed an approximately one-hour survey consisting of a panel of personality questionnaires and performance tests [137]. The results showed that individuals infected with *Toxoplasma* scored lower in conscientiousness, openness to new experiences, pathogen disgust, injury disgust, Machiavellianism, and tribalism, while scoring higher in intelligence. They also scored lower in physical and mental health. However, follow-up multivariate tests (path analysis and partial Kendall correlation controlled for either physical or mental health) clearly showed that neither physical nor mental health mediated the observed differences in personality between *Toxoplasma*-infected and *Toxoplasma*-free subjects.

The second study, which focused on the relationship between *Toxoplasma* infection, physical health, stress, and anxiety, yielded opposite results [26]. This cross-sectional study was conducted on a sample of 552 *Toxoplasma*-free and 146 *Toxoplasma*-infected subjects, who completed a comprehensive health questionnaire and a panel of performance tests. The results again showed that *Toxoplasma*-infected individuals had poorer physical and mental health and significantly higher anxiety levels, as measured by the State-Trait Anxiety Inventory X-2. However, the subsequent path analysis revealed that *Toxoplasma* influences anxiety only indirectly—primarily by affecting physical health, which in turn influences stress levels (measured by the Perceived Stress Scale), and this stress then affects anxiety. This study therefore supported the side-effect hypothesis, indicating that the increased anxiety observed in *Toxoplasma*-infected individuals is not the result of specific manipulation by the parasite but rather a side effect of the deteriorating health caused by long-term infection.

In summary, some changes in personality and behavior observed in *Toxoplasma*-infected individuals may simply result from deteriorating physical health, which can affect mood, cognition, and stress levels in ways that contribute to behavioral alterations. However, other changes appear to occur independently of general health decline and may instead reflect more direct effects of the parasite on the nervous system or neurochemical pathways. These effects could, though not necessarily, as discussed in detail by Flegr [196], represent adaptive forms of parasitic manipulation aimed at enhancing the transmission to definitive hosts, or alternatively, they may be incidental by-products of infection with no evolutionary function.

### 6.2. Toxoplasma Infection and Reproductive Outcomes

#### 6.2.1. Effects of *Toxoplasma* Infection on Pregnancy

*Toxoplasma* infection, though often considered clinically silent in immunocompetent individuals, may exert subtle effects on the physiology of pregnancy. Accumulating evidence suggests that chronic infection with *Toxoplasma* can influence maternal parameters even in the early stages of gestation. One study, for instance, reported that *Toxoplasma*-infected women had a significantly lower body weight in the 16th week of pregnancy compared to uninfected women [197]. In addition, the study employed a permutation test to statistically account for false negatives—cases in which anti-*Toxoplasma* antibody levels were so low that individuals were classified as seronegative not due to the absence of infection but because of a long-standing infection. Using this approach, the researchers found a negative correlation between weight and the duration of infection in infected women. This finding suggests that the negative impacts of the infection on body weight were not simply the effects of acute and transient infection but reflect the gradual and cumulative influence of *Toxoplasma* infection. This study also reported that *Toxoplasma*-infected women had a longer duration of gravidity, estimated from the date of last menstruation, possibly either because of fetus growth retardation in *Toxoplasma*-infected women or a shift of fertilization into the late phases of the menstrual cycle. In this regard, another study also showed that multiparous *Toxoplasma*-infected pregnant women had on average a 1.3 days longer pregnancy and developmentally younger fetuses compared with uninfected pregnant women at pregnancy week 16 [198].

RhD phenotype also seems to play a role in the association between *Toxoplasma* infection and pregnancy health issues in infected women. In one study performed on 785 *Toxoplasma*-negative and 194 *Toxoplasma*-positive pregnant women, RhD-negative, 27 *Toxoplasma*-infected pregnant women gained approximately 1600 g more weight by the 16th week of pregnancy compared to other groups [199]. Specifically, the average weight gain at week 16 was 4.12 kg in RhD-negative, *Toxoplasma*-infected women, compared to 2.44 kg in RhD-negative, *Toxoplasma*-free women, 2.65 kg in RhD-positive, *Toxoplasma*-free women, and 2.35 kg in RhD-positive, *Toxoplasma*-infected women. Notably, this weight difference remained approximately constant throughout the remainder of pregnancy.

Another cross-sectional study, among other findings, found that *Toxoplasma*-infected pregnant women had a higher prevalence of gestational diabetes mellitus in the 24–28th gestational weeks of gravidity compared with uninfected women, and significantly higher blood glucose levels according to the oral glucose tolerance test [200]. In addition, the glucose level was positively associated with the anti-*Toxoplasma* antibodies levels, indicating that the glucose level decreases as the duration of infection increases. The authors suggested that the observed increased levels of glucose might be important in the development of the metabolic syndrome and type 2 diabetes and possibly also in offspring sex ratio shifts in *Toxoplasma*-infected women, see Section 6.2.2 and Section 7.3.3.

*Toxoplasma*-positive and -negative pregnant women also differ in thyroid hormone levels. In a cross-sectional study of 1248 pregnant women in the 9th–12th gestational week, serum thyroid-stimulating hormone, thyroperoxidase antibodies, and free thyroxine were assessed using chemiluminescence, and *Toxoplasma* status was determined via the complement fixation test (CFT) and enzyme-linked immunosorbent assay (ELISA) [201]. Latent toxoplasmosis was detected in 22.5% of women, while autoimmune thyroid disease was identified in 14.7%. *Toxoplasma*-positive women had significantly higher levels of anti-thyroperoxidase antibodies (*p* = 0.004) and slightly lower levels of thyroid-stimulating hormone (*p* = 0.049) than uninfected women. Additionally, authors found a positive correlation of free thyroxine and concentration of anti-*Toxoplasma* IgG antibodies measured with ELISA (*p* = 0.033) or CFT (*p* = 0.009). Although the *Toxoplasma*-associated hormonal changes were mild and likely clinically insignificant, they could provide insights into the complex pathogenesis of autoimmune thyroid diseases.

#### 6.2.2. Effect of *Toxoplasma* Infection of a Mother on Postnatal Development of Children

Some findings suggest that toxoplasmosis may affect not only the course of pregnancy and fetal development but also the postnatal development of the child [202]. In this study, 1000 mothers who had been tested for *Toxoplasma* infection during pregnancy were contacted by email about two years after delivery and asked to complete a questionnaire assessing their children’s development during the first 18 months of life. A total of 331 women responded, including 53 who were *Toxoplasma*-positive.

Birth weights and lengths did not differ significantly between the infants of infected and uninfected mothers in this study. However, the infants of *Toxoplasma*-positive mothers exhibited significantly greater weight gain during regular check-ups until 18 months of age compared to those of *Toxoplasma*-negative mothers (GLM for repeated measures: F = 4.86, *p* = 0.028). The effect of the child’s sex was also significant (F = 23.80, *p* < 0.001), but the interaction between *Toxoplasma* infection and sex was not (F = 0.14, *p* = 0.707). Neither *Toxoplasma* infection (F = 2.26, *p* = 0.134) nor the *Toxoplasma* infection–sex interaction (F = 0.02, *p* = 0.880) had any effect on infant height.

During the first 18 months, slower postnatal motor development was reported in infants of *Toxoplasma*-positive mothers (GLM for repeated measures with age of motor milestones as the dependent variable: F = 4.238, *p* = 0.041). In post hoc tests, infants of infected mothers began to control their heads significantly later, started to turn from supine to prone later, and showed a nonsignificant delay in the onset of crawling.

A major limitation of this study was the inability to determine whether the observed effects genuinely reflected differences in children’s postnatal development or rather differences in how infected and uninfected mothers assessed their children’s development. Additionally, even if the observed effects were real, it remains unclear whether they resulted from physiological differences in the children or from differential maternal behavior toward the offspring.

#### 6.2.3. Effects of *Toxoplasma* Infection on Male-to-Female Birth Ratios

*Toxoplasma* infection has been also shown to be associated with the sex ratio of the offspring in pregnant women. A study, using the indirect immunofluorescence test (IIFT), demonstrated that the probability for a male child’s birth significantly grew as anti-*Toxoplasma* antibodies titers increased in pregnant women [203]. It is found that levels of anti-*Toxoplasma* antibodies moderate this relationship so that women with higher concentrations (apparently attributed to recent infection) have higher secondary sex ratios, and women with lower concentrations (presumably corresponding to old infections) have lower secondary sex ratios [204]. In this respect, a review article suggested that maternal hormonal changes (specifically estrogen) in infected females may explain this variation in the sex ratios of infected mothers’ offspring [205]—but also see [206]. Researchers suggest that, in line with the Trivers–Willard hypothesis, increased health burdens and chronic stress may signal unfavorable maternal conditions, thereby reducing the proportion of male births [204]. Similar findings were observed in laboratory mice, where those infected shortly before conception exhibited a significantly increased secondary sex ratio, whereas those infected six months prior to conception showed a markedly decreased ratio [207]. However, it must be noted that, in contrast to humans, in standard laboratory mice, chronic *Toxoplasma* infection is factually characterized as a post-acute phase rather than true latency, often involving ongoing immune activity and neuroinflammation.

Finally, an ecological study examining the correlation between *Toxoplasma* seroprevalence in women of childbearing age and the secondary sex ratio in different countries identified *Toxoplasma* seroprevalence as the third most significant predictor among 15 studied factors—ranking after son preference and the number of children per woman, but ahead of maternal age, polygyny intensity, wealth, latitude, humidity, sanitation rate, parasite stress, nutritional stress, contraceptive use, health status, cat ownership, and meat consumption [208]. The study found that in populations with high *Toxoplasma* seroprevalence—where infection typically occurs in early life and persists in latent form—women tend to give birth to fewer sons than daughters. This aligns with prior evidence suggesting that chronic *Toxoplasma* infection may bias offspring sex ratio toward females, whereas acute infections during reproductive age may transiently increase the likelihood of male births.

A possible explanation for the observed initial increase in the secondary sex ratio in both women and female mice shortly after infection is parasite-driven manipulation, potentially aimed at immunosuppression. In contrast, the most plausible explanation for the later decline in the secondary sex ratio is the gradual deterioration of health in infected women [204]. As predicted by the Trivers-Willard hypothesis and supported by empirical data, women and female animals in poorer health tend to give birth to more daughters than sons [209]. Thus, the reduced secondary sex ratio in long-term infected women can be considered indirect evidence of progressive health deterioration in individuals infected with *Toxoplasma*.

#### 6.2.4. *Toxoplasma* Infection and Human Fertility

##### Male Fertility

A growing body of both direct and indirect evidence suggests that *Toxoplasma* infection may negatively affect male fertility. *Toxoplasma* has been identified in the male reproductive organs of both animals and humans [210,211,212,213], where its presence is often linked to inflammation, hypogonadism, and compromised sperm quality and quantity [214]. Several studies have reported a higher *Toxoplasma* seroprevalence in subfertile men. For instance, a Chinese study found significantly higher *Toxoplasma* seroprevalence among men with fertility problems compared to controls, along with a greater incidence of anti-sperm antibodies in infected individuals [215]. Similarly, a preregistered study of 669 men attending a fertility clinic in Prague reported a higher rate of fertility problems and reduced sperm parameters in *Toxoplasma*-positive men, effects that were significant only among smokers, suggesting a potential interaction between smoking and infection [216]. Additional population-based research has shown that infected men fathered fewer children than uninfected controls, an effect not observed in women [216]. However, another large-scale study found a significant association between *Toxoplasma* infection and self-reported fertility problems in women, but not in men, despite a comparable effect size in both sexes [97].

It is known that *Toxoplasma*-infected men (as well as laboratory-infected male rats) exhibit elevated testosterone levels [79,106]. While increased testosterone levels could positively influence sexual activity and thus, particularly in non-human animals, fertility, this elevation is likely only temporary, as thoroughly discussed in Section 7.2.1. Moreover, in some species, such as mice, this increase may not occur at all. Furthermore, the negative non-specific effects of the infection on physical health (Section 6.1) and its specific effects on sperm quality likely outweigh any potential positive impact of elevated testosterone levels.

A study conducted on 2044 individuals (517 infected) revealed that infected men had significantly fewer children of their own than uninfected men. In the same study, infected men also reported higher sexual activity but slightly lower sexual desire than uninfected men [216]. However, likely due to the lower number of infected men in the sample (77), even a relatively strong effect of infection on sexual activity (Tau = 0.051) was not significant. In women from the same study, a negative effect of infection on sexual desire was demonstrated, though the negative effects of infection on sexual activity and on the number of their own children were not significant.

##### Female Fertility

Several cross-sectional studies have suggested a potential link between latent *Toxoplasma gondii* infection and reduced female fertility. A 2015 study analyzing data from four distinct populations reported that *Toxoplasma*-infected women tended to be older and more likely to use assisted reproductive technologies (ARTs) than uninfected women [217]. In two hospital-based cohorts (studies A and B), fertility outcomes were assessed via clinical records, while in two other groups (studies C and D), women self-reported fertility issues. Across all samples, infected women, particularly primiparas, were significantly older at conception and more frequently required ART. Additionally, in the self-report studies, *Toxoplasma*-positive women reported longer times to conceive and more severe fertility difficulties.

The relationship between *Toxoplasma* infection and miscarriage has also been explored. Pavlinová et al. [218] found a significantly higher seropositivity rate in women with recurrent miscarriages compared to the controls (42.1% vs. 25.1%). Similar trends were observed in a large cross-sectional study showing an increased risk of recurrent miscarriage among infected women (odds ratio = 2.07; *p* = 0.031) [97]. Further evidence comes from studies in Iran reporting the presence of *Toxoplasma* DNA in the fetoplacental tissues of women with spontaneous abortions [219,220]. However, not all studies have supported this association [221,222].

A comprehensive meta-analysis of 41 studies (n = 5693 cases; 8667 controls) provided stronger evidence, showing that *Toxoplasma* infection was associated with nearly twice the risk of spontaneous abortion (odds ratio = 1.84; 95% CI: 1.41–2.40). The risk was even higher for recent infections, indicated by IgM seropositivity (odds ratio = 3.72; 95% CI: 2.21–6.26) [223]. Despite heterogeneity across studies, no publication bias was detected.

Collectively, existing data suggest that latent toxoplasmosis may substantially affect reproductive outcomes, including fertility and pregnancy maintenance, highlighting the broader reproductive health risks associated with this prevalent infection. Given the variability in study findings, further well-designed research is needed to clarify the mechanisms underlying these associations. Future investigations should prioritize longitudinal studies that track *Toxoplasma* seroconversion and antibody titers before conception and throughout pregnancy, along with hormonal levels (e.g., estrogen, progesterone) and key reproductive outcomes such as ovulation, implantation, and pregnancy maintenance. These designs would help clarify the timing and causal relevance of infection. In addition, targeted research on high-risk groups, such as women with unexplained infertility, repeated pregnancy loss, or failed in vitro fertilization despite normal embryo quality, could help identify whether chronic *Toxoplasma* infection contributes directly to reproductive dysfunction in otherwise unaccounted-for cases.

For a summary of the findings of this chapter, see Table 6.

## 7. Biology of the Mechanisms Underlying the Effects of *Toxoplasma* Infection

**Figure 8:**
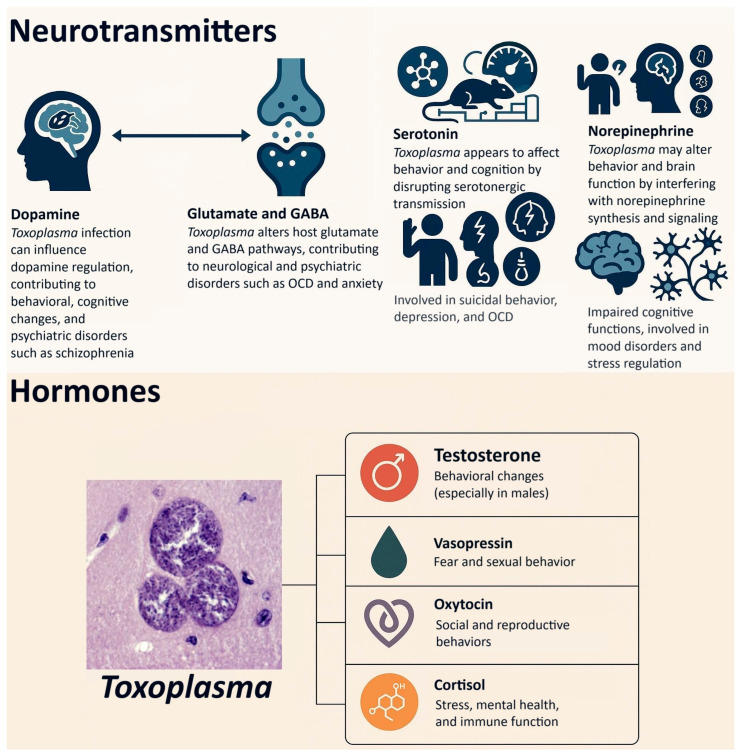
Molecular mechanisms of behavioral effects of chronic *Toxoplasma* infection.

For a few decades now, scientists have been studying the impact of *Toxoplasma* infection mainly on the host’s behavior, cognition, and health, with a recent focus on understanding the biological processes involved. In the following sections, the primary candidates responsible for these effects will be discussed. It is important to note that this list is not exhaustive (see Figure 8), and the mechanisms included are not explored in complete detail. Moreover, we acknowledge that these mechanisms interact synergistically, and their classification as separate entities is largely artificial. Last but not least, animal models may reflect a prolonged acute response rather than true latency. As such, caution is warranted when extrapolating these results to the latent, likely life-time cyst-forming infection typical in humans.

### 7.1. Neurotransmitters

This section addresses the potential role of neurotransmitters in *Toxoplasma*-induced alterations in behavior, cognition, and mental health. The organization of the following subsections reflects a progression from core dopaminergic mechanisms, which are well-established in the context of *Toxoplasma*-related behavioral modulation, to neurotransmitters involved in excitation–inhibition balance (glutamate and GABA), followed by those with modulatory and mood-related effects (serotonin), and finally to norepinephrine, a more recently implicated pathway with immunological relevance. This order facilitates a coherent understanding of how different neurotransmitter systems may contribute to the complex neuropsychological outcomes of infection.

#### 7.1.1. Dopamine

Dopamine is an important monoamine neurotransmitter in the brain, with a variety of receptors (D1, D2, D3, D4, and D5), which play an eminent role in many brain functions such as reward behavior, sleep, learning, mood regulation, attention, etc.

Animal model studies suggest that *Toxoplasma*-induced behavioral alterations may be in part modulated by its effects on the regulation of dopamine in the brain. The findings of an early study revealed that *Toxoplasma* infection led to a decrease in locomotion in the open field test for both male and female mice. However, infected females exhibited an increased level of exploration during the holeboard test. When subjected to a selective dopamine uptake inhibitor, GBR 12909, the infected males showed suppressed exploration in the holeboard test, in contrast to control males who experienced an increase in exploratory behavior [41].

Molecular studies also revealed that *Toxoplasma* was directly responsible for an increase in dopamine production in the brain, likely through the expression of two genes encoding tyrosine hydroxylase enzymes, which converts tyrosine into L-DOPA—a precursor of dopamine—in host neurons [22,39]. In addition to tissue and cell culture studies demonstrating that *Toxoplasma* increases dopamine metabolism [147], a human study found, among other results, that *Toxoplasma*-infected individuals exhibited reduced sensitivity to reward in tasks designed to assess reward responsiveness compared to healthy controls. The authors of this study attributed this phenomenon to the elevated levels of dopamine induced by the *Toxoplasma* infection in those affected by the parasite [224]. Indirect behavioral evidence obtained through personality questionnaires such as the Temperament and Character Inventory [74,225] has similarly provided empirical support for the hypothesis that the *Toxoplasma*-induced upregulation of dopamine may play a role in behavioral alterations observed in infected individuals. Specifically, infected subjects were found to score lower in novelty seeking—a personality trait closely linked to dopaminergic activity.

The effects of toxoplasmosis on cognition may also, in part, stem from *Toxoplasma*-induced alterations in dopamine regulation. In this respect, an animal study showed that young adult males infected with *Toxoplasma* exhibited significant impairment in visual attentional selection under conditions of low perceptual load [226]. This finding aligns with evidence from other animal model studies indicating that attentional processes are, at least in part, modulated by dopaminergic signaling [227,228]. In humans, dopamine has been shown to play an important role in cognitive performance and intelligence [229]. In line with this, a study found that *Toxoplasma*-infected men exhibited reduced intelligence scores—traits that may be partially influenced by parasite-induced alterations in dopaminergic signaling [72,74]. The same effects have been also reported for worsened reaction time and information processing speed [109,110,114] in *Toxoplasma*-infected subjects. Reaction time can be in part affected by changes in the levels of dopamine possibly through acting on the level of arousal [230]. These findings point to a likely connection between *Toxoplasma* infection and the resulting altered regulation of dopamine and its consequent effects on cognition.

Dopamine is also important in the etiology of mental disorders. In particular, the dysregulation of dopamine levels plays a central role in the pathophysiology of several major psychiatric disorders, including schizophrenia [231], bipolar disorder [232], and OCD [233], all of which have significant clinical relevance. As discussed earlier in Section 5, individuals infected with *Toxoplasma* have been shown to exhibit higher rates of dopamine-related disorders, including schizophrenia [234], autism spectrum disorder [150], possibly attention-deficit/hyperactivity disorder [157,235], and potentially bipolar disorder (comprehensive information can be found in [236]), as well as OCD (for a detailed discussion, see [177]). These patterns support the hypothesis that some of the adverse mental health effects observed in *Toxoplasma*-infected individuals may be at least partially mediated by the parasite’s impact on dopamine regulation in the brain.

#### 7.1.2. Glutamate and GABA

Glutamate is an excitatory neurotransmitter in the CNS, which is of importance in cognition, including memory formation and memory retrieval, motor coordination, sensory information, and emotions. It is mostly accumulated in synaptic vesicles and is taken up through astrocytic processes upon being released by the nerve terminals [237]. Regarding cognition, a study found cognitive impairment in the domain of recognition memory in rats exposed to repeated stress, which resulted in the suppression of glutamate receptor expression [238]. In addition, glutamate plays important roles in a number of other cognitive functions such as working memory [239], attention [240], and information processing [241]. Studies have also discovered relations between mental disorders and dysregulated levels of glutamate. For example, increased levels of glutamate were reported in patients with bipolar disorder and major depression [242]. The excess of glutamate is also observed to be mainly involved in the etiology of OCD [243], schizophrenia [244], autism [245], and anxiety [246]—all of which have been reported to occur with greater frequency in *Toxoplasma*-infected individuals (see Section 5).

Studies show that *Toxoplasma* alters the levels of glutamate in the CNS. For example, a study using microdialysis indicated that extracellular glutamate levels in the murine frontal cortex significantly rise during infection. In addition, it was discovered that the disruption in astrocytes’ control of glutamate leads to various deficits seen in chronic infection [25]. As previously discussed in Section 4 and Section 5, toxoplasmosis is linked to various cognitive impairments, including longer reaction times, slower information processing speeds, and deficits in executive functioning and memory. Additionally, it is associated with mental disorders such as schizophrenia, BD, and OCD. The similarities between the cognitive impairments and mental disorders associated with *Toxoplasma* infection and those resulting from dysregulation of glutamate levels suggest that some of the negative effects of *Toxoplasma* infection on mental health and cognition may occur through the glutamate pathway in the CNS.

Glutamate is the biochemical precursor for gamma-aminobutyric acid (GABA), the major inhibitory neurotransmitter in the central nervous system. Both neurotransmitters appear to be involved in host–parasite interactions at multiple levels. For instance, GABA may serve as a carbon source to support parasite metabolism and facilitate dissemination by enhancing the motility of dendritic cells—normally immune sentinels, but co-opted by *Toxoplasma* as vectors of dissemination [247].

Recent evidence highlights how immune-driven alterations in glutamine and glutamate metabolism may influence the parasite’s developmental transitions. A study by Bando et al. [248] found that the modulation of intercellular glutamine levels by the immune system can induce the stage conversion of *Toxoplasma* within glutamatergic neurons, promoting the formation of *Toxoplasma* cysts. This shift is thought to be part of the host’s mechanism for controlling the infection, albeit one that contributes to the establishment of chronic *Toxoplasma* infection.

Disruptions in glutamatergic and GABAergic cells and neurotransmission are also implicated in neurological and psychiatric disorders such as epilepsy and schizophrenia—both of which have been epidemiologically linked to *Toxoplasma* infection in humans [27,97]. In murine models, Brooks [247] demonstrated that such dysregulation can provoke seizures in infected animals, further reinforcing the neurophysiological relevance of these findings.

One proposed mechanism at the molecular level involves the parasite’s influence on glutamate decarboxylase 67 (GAD67), an enzyme responsible for GABA synthesis from glutamate. By potentially altering GAD67 expression or activity, *Toxoplasma* could shift the excitatory–inhibitory balance of neuronal networks, which may in turn affect host behavior. These disruptions in GABA production and signaling are thought to contribute not only to increased neural excitability but also to altered emotional and behavioral regulation [24,249].

Dysfunction in N-methyl-D-aspartate receptors (NMDARs)—which are glutamate-gated ion channels—has also been proposed as a mechanistic bridge between *Toxoplasma* infection and schizophrenia [250]. Since NMDARs are critical to synaptic signaling, plasticity, and neurodevelopment, their disruption may help explain some of the psychiatric manifestations observed in a subset of chronically infected individuals [24,249].

These biochemical alterations also extend to behavior. A recent study by Acquarone et al. [251] found that *Toxoplasma*-infected animals exhibited reduced startle reflex amplitudes—an indicator of altered sensory and emotional processing—accompanied by decreased glutamate and D-serine levels in both the prefrontal cortex and hippocampus. These regions are essential for executive function, emotional regulation, and memory, suggesting that glutamatergic signaling deficits may contribute to the broader neurobehavioral phenotype observed in *Toxoplasma*-infected subjects.

In summary, there is increasing evidence that at least some of the negative effects of *Toxoplasma* on the health and cognition of its host are mediated through the glutamate and GABA pathways. These disruptions may underlie a range of neuropathological outcomes, from seizure susceptibility to psychiatric problems, and help explain the observed links between *Toxoplasma* infection and neurobehavioral disorders.

#### 7.1.3. Serotonin

The importance of serotonin in a large number of bodily functions ranging from motor activity and sleep-wake cycle to cognitive functions and emotions has been discovered for some time now [252,253,254]. Tryptophan, a biochemical precursor of serotonin [255], plays an important role in the efficient growth of *Toxoplasma* in the host cells. For example, research shows that the administration of recombinant gamma interferon causes the degradation of tryptophan; hence, restricting the growth of *Toxoplasma* in human fibroblasts [256]. This cytokine plays an important role in cell mediated immunity and orchestrates the immune system’s response to pathogens and malfunctioning cells through its immunomodulatory capabilities [257]. It particularly induces Indoleamine 2,3-dioxygenase (IDO), a key enzyme that inhibits many intracellular parasites, including *Toxoplasma*, by depleting tryptophan [258]. However, this subsequently reduces the levels of serotonin.

For example, a study of 25 female Wistar rats, half of which were injected with 5 × 105 tachyzoites of *Toxoplasma* (virulent RH strain) intra-peritoneally, showed that four months after the injection, the infected rats had significantly lower levels of serotonin in the brain compared with the control group. Interestingly, the levels of serotonin did not significantly differ in the blood of the case and control groups [259]. In contrast, other research reported higher levels of serotonin in the plasma of *Toxoplasma*-infected sheep compared to uninfected controls [23]. It is possible that the effects of the infection on serotonin levels vary depending on the species of the host, whether the measurements are taken from plasma or brain tissue, or other unknown factors.

Studies also show that psychiatric disorders such as bipolar disorder, depression, ADHD, schizophrenia, autism, anxiety disorders, and addiction are in part caused by the dysregulation of the tryptophan pathways (hence, serotonin dysregulation) [260,261]. Serotonin dysregulation has been implicated in several psychiatric conditions, including suicidal behavior [262], depression [263], impulsive aggression [264], and OCD [265]. Studies have also revealed associations between toxoplasmosis and suicidal behavior [266] and impulsivity and aggression [267]; however, as already mentioned in Section 5, the case of depression remains controversial as many studies show opposite findings. These controversies may be in part due to the role of sex, RhD genotype, or other factors which may not have been given due attention in this line of research. In this regard, the toxoplasmosis–serotonin mechanism may be partly involved in the etiology of OCD, suicidal behavior, impulsive and aggressive behavior, and possibly depression.

An emerging explanation for the influence of *Toxoplasma* on host behavior highlights the significance of astrocyte activation during the immune response. When *Toxoplasma* infection triggers neuroinflammation, astrocytes become partially activated and metabolize tryptophan into kynurenic acid (KYNA). This diversion not only reduces serotonin availability but also leads to an accumulation of KYNA, a neuroactive metabolite known to antagonize key neurotransmitter receptors such as the N-methyl-D-aspartate receptor (NMDAR) and the alpha-7 nicotinic acetylcholine receptor (α7-nAChR). By blocking these receptors, KYNA may impair cognitive performance and contribute to neuropsychiatric dysfunction [24,249].

Taken together, these findings suggest that *Toxoplasma* exerts a multifaceted influence on host neurochemistry by engaging astrocyte-mediated pathways and modulating serotonergic transmission. Through this interference, the parasite may compromise cognitive function and affect emotional regulation.

#### 7.1.4. Norepinephrine

A more recent hypothesis suggests that *Toxoplasma* may exert behavioral manipulation through its impact on the norepinephrine pathway. Specifically, the parasite appears to downregulate the expression of the gene encoding dopamine β-hydroxylase (DBH), the enzyme responsible for converting dopamine into norepinephrine. Alsaady et al. [268] show that this suppression leads to a marked reduction in norepinephrine levels in both human and rat neuronal cultures. This downregulation of DBH is increasingly recognized as a likely contributor to the behavioral changes observed in infected hosts.

The consequences of norepinephrine depletion extend beyond behavior to include cognitive and neuroimmune dysfunction. Norepinephrine plays a crucial role in regulating neuroinflammatory responses by influencing microglial cells [269] and the glial and peripheral immune cells that are recruited into the brain, as shown by Laing et al. [270]. Laing et al. suggest that diminished norepinephrine levels impair this regulatory signaling, leading to heightened proinflammatory activity within the central nervous system in rodents with latent toxoplasmosis. This inflammatory cascade may disrupt synaptic function and contribute to the cognitive impairments often associated with chronic infection.

Furthermore, norepinephrine dysregulation has been implicated in a range of neuropsychiatric conditions. In particular, the dysfunction of the locus coeruleus–norepinephrine pathway has been likely associated with aberrant attentional and reward processing [271], as well as to depression and mood disorders [272,273], autism spectrum disorder [274], impaired stress regulation [275], and schizophrenia [276]. In this respect, a recent review by Cairney and McConkey [277] emphasizes the potential role of *Toxoplasma*-induced norepinephrine suppression in mediating neuropsychiatric disorders and cognitive impairments, pointing to norepinephrine dysregulation as one of the likely mechanisms.

Together, these findings support an emerging model in which *Toxoplasma* not only manipulates neurotransmission to alter host behavior but also exerts broader effects on brain function by interfering with norepinephrine synthesis and signaling. This pathway likely contributes to the complex neurochemical web through which *Toxoplasma* may induce long-term alterations in cognition, mental health, and immune regulation.

### 7.2. Hormones

The following section explores the hormonal pathways affected by *Toxoplasma* infection. In this context, testosterone is presented first due to its central and extensively studied role in *Toxoplasma*-induced behavioral changes, especially in males. This is followed by vasopressin, which is functionally and mechanistically linked to testosterone in fear–sexual behavior modulation. Oxytocin is then addressed, emphasizing its role in socioemotional and reproductive behavior, particularly in females. Finally, cortisol is discussed to provide insight into systemic stress responses and their interplay with mental health and immune function in the context of *Toxoplasma* infection.

#### 7.2.1. Testosterone

Testosterone, a steroid hormone primarily known for its role in primary and secondary sexual development in males, exerts a wide range of physiological and psychological effects that extend beyond reproduction. In the context of *Toxoplasma* infection, testosterone has been identified as a key modulator of host behavior, potentially manipulated by the parasite to enhance its transmission [79,106].

Experimental findings from rodent models provide compelling evidence for this mechanism. *Toxoplasma* is believed to induce epigenetic changes in the medial amygdala of infected male rats, leading to elevated testicular testosterone production. These changes affect the extra-hypothalamic vasopressinergic system, which plays a central role in regulating sexual and social behaviors [21]. According to Hari Dass and Vyas, *Toxoplasma* exploits the host’s phenotypic plasticity by demethylating specific gene promoters, activating latent behavioral pathways related to sexual arousal and fear processing. Infected male rats, as a result, exhibit reduced aversion to predator odors—such as cat urine—which would normally trigger strong avoidance responses [21].

Further experimental validation comes from studies in which exogenous testosterone was administered directly to the medial amygdala of castrated rats. The treatment successfully reproduced the behavioral effects observed in *Toxoplasma*-infected animals—namely reduced predator fear—while castration eliminated these behavioral and molecular responses altogether [278]. These findings suggest that it is not necessarily the presence of the parasite cysts in the brain, but the downstream hormonal alterations, particularly in gonadal steroids, that mediate behavioral change. Nevertheless, the authors acknowledge two important caveats: first, *Toxoplasma* infection also affects neurotransmitter processing in the brain; second, infected female rats—despite having significantly lower endogenous testosterone—also display similar behavioral shifts, implying additional sex-independent mechanisms.

In humans, testosterone serves a broad range of functions beyond its reproductive role. It regulates libido, sexual arousal, and erectile function [279], influences bone metabolism by modulating osteoclast activity, supports the maintenance of muscle mass, affects mood and cognitive performance, and modulates immune responses [280]. Its connection to mental health is complex. As summarized in a recent review, testosterone may exert both protective and risk-enhancing effects depending on context [281]. For instance, based on this review, elevated levels have been linked to reduced positive symptoms in first-episode psychosis and delayed puberty in individuals with early-onset schizophrenia, but also to increased interpersonal difficulties in borderline personality disorder and higher rates of alcohol misuse in adolescents. Moreover, elevated prenatal testosterone has been implicated in amplifying symptoms of autism spectrum disorder, while lower levels are associated with increased susceptibility to depression and anxiety, particularly in men.

The potential link between *Toxoplasma* infection and testosterone modulation was first suggested in a study on the 2D:4D digit ratio—the ratio of the second to fourth fingers—a known biomarker of prenatal testosterone exposure [106]. This study, conducted on 269 university students (49 of whom were infected with *Toxoplasma*), reported significantly lower 2D:4D ratios in both male and female infected individuals, indicating elevated prenatal testosterone exposure. To corroborate this, the researchers measured actual circulating hormone levels and found that infected men had significantly elevated testosterone levels, while infected women exhibited decreased levels compared to their uninfected counterparts. These observations were later substantiated by multiple studies. One showed increased total and free testosterone in *Toxoplasma*-infected men [282], another observed elevated levels in infected pregnant women across all three trimesters [283], and a third reported higher serum testosterone in infected individuals compared to healthy controls in both men and women [284].

However, the interpretation of these findings is still debated. Flegr and Kuba proposed that elevated testosterone may represent a transient response occurring in the post-acute phase of infection, which subsequently diminishes during chronic toxoplasmosis [95]. Supporting this hypothesis, a recent meta-analysis showed that the pooled mean testosterone concentrations in infected men and women were moderately elevated—by 0.73 and 0.55 units, respectively—yet it also revealed substantial variability across studies, indicating a more context-dependent relationship [38].

Moreover, some findings appear contradictory. While testosterone is generally associated with anxiolytic and antidepressant effects—mediated in part via aromatization into estradiol in the dentate gyrus and binding to androgen receptors in brain regions such as the basolateral amygdala and the suprachiasmatic nucleus [285,286,287]—increased anxiety and depressive symptoms have nonetheless been reported in chronically infected individuals, see Section 5.5 and Section 5.6. This apparent paradox may be explained by the temporal model advanced by Flegr and Kuba [95], which posits that testosterone elevations are short-lived and decline over time, potentially diminishing their protective emotional effects during chronic infection.

In sum, testosterone represents a key hormonal interface through which *Toxoplasma* may exert its behavioral influence on the host. While its manipulation appears to facilitate adaptive behavioral changes in animal models—particularly involving sexual arousal and predator aversion—the implications in humans remain complex. The effects are likely shaped by a combination of hormonal timing, infection stage, sex, and interaction with other neurobiological systems.

#### 7.2.2. Vasopressin

Vasopressin (VP), also known as arginine vasopressin (AVP), is a neuropeptide that serves dual roles as a hormone and neurotransmitter. While its classical function lies in maintaining fluid balance via antidiuretic effects mediated by the posterior pituitary [288], vasopressin also plays a key role in central nervous system regulation of social behaviors, emotional responses, aggression, and parental care [289,290]. It is increasingly recognized as a major integrator of genetic and environmental signals influencing complex behavioral phenotypes, including those implicated in neuropsychiatric disorders.

In the context of *Toxoplasma* infection, vasopressin has drawn attention for its interaction with testosterone in modulating fear and social behaviors. In male rodents, *Toxoplasma*-induced behavioral changes—particularly the loss of innate aversion to predator odors—have been mechanistically linked to epigenetic modifications in the vasopressin system. Specifically, infection leads to the hypomethylation of the AVP promoter region in the posterodorsal medial amygdala, which enhances the expression of vasopressin and increases the activation of vasopressinergic neurons when the animal is exposed to cat scent [21]. This altered neuroendocrine signaling results in a blunted fear response, which has been interpreted as a parasite-facilitated adaptation aimed at enhancing transmission to the definitive feline host.

These behavioral effects appear to be testosterone-dependent. The interaction between vasopressin and testosterone likely involves overlapping neural circuits that regulate sexual motivation and defensive behavior. According to one influential hypothesis [21], *Toxoplasma* infection leads to a “leakiness” between the circuits for fear and reproduction, causing stimuli that would normally evoke avoidance—such as predator odor—to instead elicit attraction or investigatory behavior. Vasopressin appears to mediate this circuit leak by modulating the salience and valence of olfactory cues through its action in the medial amygdala and other brain areas associated with sociosexual behavior. In this regard, a study notably demonstrated that infected male rats that were castrated did not show the same loss of predator aversion, nor did they exhibit the associated epigenetic changes in vasopressin expression. Conversely, exogenous testosterone administration was sufficient to re-induce the loss of fear and the molecular signature of vasopressin system activation [278]. These findings point to a synergistic relationship between gonadal steroids and neuropeptides in mediating parasite-induced behavior, with testosterone acting as a permissive factor that enables vasopressin-driven changes to emerge.

Although the bulk of current research on *Toxoplasma*-vasopressin interactions has been conducted in male rodents, these findings raise important questions about whether similar mechanisms may operate in humans. Vasopressin is known to influence human behaviors related to social recognition, stress reactivity, and emotional bonding. Polymorphisms in the vasopressin receptor gene (AVPR1A) have been linked to variation in empathy, pair-bonding behavior, and susceptibility to mood disorders [289]. The dysregulation of vasopressin signaling has also been associated with psychiatric conditions such as anxiety and depression [291]—behaviors known to be affected in *Toxoplasma*-infected individuals.

Moreover, vasopressin’s role as a mediator of the stress response, in conjunction with its interaction with the hypothalamic–pituitary–adrenal (HPA) axis, positions it as a potential target of parasite-driven manipulation aimed at altering host stress reactivity. Excessive vasopressin activity has been associated with hyper-anxiety and depressive-like behaviors, while its deficiency may contribute to hypo-anxiety and disorders such as diabetes insipidus [289]. Whether *Toxoplasma* differentially affects these states across sexes, infection stages, or personality traits remains to be investigated.

In conclusion, vasopressin is emerging as a critical node in the neurohormonal network influenced by *Toxoplasma*. Its interaction with testosterone, coupled with its epigenetic regulation in key brain regions, supports its role as a mediator of infection-induced behavioral change—at least in males. While human research in this area is limited, the existing rodent data suggest that vasopressin may be a viable candidate for understanding the socioemotional and psychiatric effects of latent toxoplasmosis. Future research should prioritize translational studies that examine vasopressinergic signaling in *Toxoplasma*-infected human populations, ideally through multimodal approaches. These might involve neuroimaging techniques, such as fMRI or PET using vasopressin receptor ligands, to map brain activity and receptor distribution in social-emotional circuits. Concurrently, endocrine profiling could assess vasopressin and testosterone levels in infected versus uninfected individuals, while epigenetic analyses of AVP gene methylation in accessible tissues may offer insight into regulatory mechanisms. Behavioral phenotyping focused on traits such as aggression, anxiety, and social bonding would further elucidate functional outcomes. In addition, stratifying populations by sex, age, and genetic variants in vasopressin receptors could help identify susceptible subgroups. Integrating these elements will clarify the role of vasopressin in shaping the host’s emotional and motivational repertoire in the context of latent toxoplasmosis and may help establish a link to its psychiatric manifestations.

#### 7.2.3. Oxytocin

Oxytocin is a neuropeptide well recognized for its role in facilitating a wide spectrum of social behaviors, ranging from maternal care and pair bonding to trust, empathy, and social memory. It also plays a significant role in the attenuation of fear responses and emotional regulation in both animals and humans [292]. Human studies show that oxytocin, when administered intranasally following Pavlovian fear conditioning, produces a non-specific suppression of amygdala activity in both the early and late phases of conditioning in men [293]. In another research study involving 52 healthy men who underwent fMRI scans while performing a social approach–avoidance task, it was found that oxytocin reduced activation in the amygdala when participants approached threats [294]. Regarding women, some studies show that it can reduce background anxiety showing anxiolytic effects [295] and other studies suggest that it enhances the detection of threatening stimuli in the environment [296]. Nevertheless, based on its profound effects on socioemotional functioning, oxytocin has recently emerged as another potential hormonal target manipulated by *Toxoplasma* to influence host behavior.

Evidence from animal models, particularly in female rodents, indicates that *Toxoplasma* infection modulates oxytocinergic signaling in a sex-specific manner. Infected female rats have been shown to express increased levels of oxytocin mRNA and upregulated oxytocin receptors in specific brain regions implicated in sociosexual behavior and emotional regulation [297]. These molecular alterations may underlie the observed behavioral changes in infected females, such as reduced fear and increased approach behavior toward ambiguous or potentially risky stimuli. This reduction in defensive behavior is consistent with the broader theme of parasite-induced phenotypic shifts that may facilitate host predation by felids, the definitive host of *Toxoplasma*.

Although human research on *Toxoplasma*-oxytocin interactions remains in its early stages, the emerging data point toward meaningful physiological effects. A recent study conducted in Mosul investigated oxytocin levels in 150 women aged 20–35 years who had experienced spontaneous abortion, comparing *Toxoplasma*-infected (n = 90) and uninfected (n = 60) control groups [298]. Results revealed significantly higher circulating levels of oxytocin (644 ± 205 pg/mL) in the infected group compared to controls. Based on the previously mentioned effects of oxytocin on human behavior, alterations in oxytocin levels induced by *Toxoplasma* infection may partially explain the changes in behavioral tendencies observed in infected individuals, including increased social risk-taking or attenuated threat appraisal—patterns that, in our evolutionary ancestors, could have increased contact with cats or environments favorable for transmission.

Oxytocin is also deeply entwined with psychiatric outcomes. Lower levels of peripheral oxytocin have been associated with disorders such as schizophrenia, anorexia, and borderline personality disorder, and elevated levels of this hormone have been found in patients diagnosed with bipolar I disorder and OCD [299]—all of which have also been linked to *Toxoplasma* seropositivity in epidemiological studies [300]. Importantly, our review found that infected women exhibited higher oxytocin levels, which appears inconsistent with the general association between low oxytocin and many psychiatric conditions. This apparent paradox highlights the complexity of oxytocin’s role in neuropsychiatric health. It is possible that the elevated oxytocin levels observed in females reflect a compensatory response or interact with other neurochemical changes induced by the infection, and both of these processes might have been modulated by sex-specific mechanisms. Another explanation, which considers the psychological strategies women use to cope with stress, is that elevated oxytocin levels in infected women may be a compensatory response to the stress associated with the infection, potentially aimed at mitigating anxiety or promoting social bonding, which are mechanisms that might be particularly relevant in the context of a chronic infection. Given that the etiology of these disorders is multifactorial, involving a network of hormonal and neurotransmitter systems, the effects of *Toxoplasma* on psychiatric outcomes are likely modulated by this broader psychobiological context.

Collectively, these findings suggest that oxytocin may serve as a key neuromodulator in the behavioral outcomes of latent toxoplasmosis, particularly in females. By modifying oxytocin levels and receptor expression, *Toxoplasma* may exert subtle but meaningful influence over emotional reactivity, social behavior, and reproductive health. However, the underlying mechanisms remain to be fully elucidated, and much of the current evidence—particularly in humans—is circumstantial. Future research should aim to disentangle the causal pathways linking *Toxoplasma* infection with oxytocin-mediated health and behavior. This could involve longitudinal cohort studies that track oxytocin levels and behavioral changes over time in infected versus non-infected individuals, with stratification by sex and hormonal status. In this respect, experimental approaches using animal models with the genetic or pharmacological manipulation of oxytocin pathways could help establish causality. Additionally, neuroimaging techniques (e.g., fMRI or PET targeting oxytocin receptors), and CRISPR-based gene editing offer promising avenues to investigate how *Toxoplasma* alters neural and hormonal function in sex-specific ways. Targeted studies in pregnant women, adolescents, or individuals with psychiatric conditions could also yield valuable insights, given the known intersections between oxytocin, reproduction, and mental health.

#### 7.2.4. Cortisol

Cortisol is the primary glucocorticoid hormone secreted by the adrenal cortex in response to the activation of the hypothalamic–pituitary–adrenal (HPA) axis. As a central mediator of the body’s stress response, cortisol regulates numerous physiological functions, including glucose metabolism, immune modulation, blood pressure, and inflammatory responses [301]. In the context of *Toxoplasma* infection, cortisol has attracted increasing interest for its role in mediating the parasite’s broader neuroimmune and behavioral effects, particularly through the modulation of stress reactivity and emotional processing.

Several animal studies have demonstrated that *Toxoplasma* infection alters circulating cortisol levels, often in parallel with behavioral changes consistent with reduced anxiety. For example, Fallah et al. [302] investigated this relationship in rats by measuring cortisol and interleukin-17 (IL-17) levels following infection. Forty rats were divided into four groups: uninfected controls, infected, infected with trimethoprim-sulfamethoxazole (TMP-SMX) treatment, and infected with dexamethasone treatment. After 30 days, behavioral tests using the plus maze were conducted, and cortisol and IL-17 levels were measured via ELISA. Infected rats showed increased entries and time spent in the open arms compared to controls (*p* < 0.01), indicating reduced anxiety. Conversely, TMP-SMX and dexamethasone-treated rats exhibited fewer open-arm entries and spent less time in the open arms. While total activity was higher in infected rats, differences were not statistically significant. These behavioral changes coincided with elevated cortisol and IL-17 levels, suggesting that *Toxoplasma* may exert the anxiolytic effects partly via HPA axis activation.

In contrast, other animal models have yielded different results. A study on wild spotted hyenas (Crocuta crocuta, n = 109) explored the relationship between *Toxoplasma* infection and hormone levels using stratified regression analysis, running separate models within sex and age class strata: cub and subadult females (model 1), adult females (model 2), cub and subadult males (model 3), and adult males (model 4). Results revealed a negative association between infection and testosterone in various age-sex classes, but no significant correlation with cortisol in any subgroup [303]. These findings underscore the importance of species-specific HPA axis regulation and environmental context in interpreting hormonal outcomes of infection.

In human studies, associations between *Toxoplasma* and altered cortisol levels are also beginning to emerge. Shirbazou et al. [304] assessed cortisol and testosterone concentrations in 180 individuals (107 males and 73 females), alongside serological testing for *Toxoplasma*-specific IgG and psychological assessments using the Depression Anxiety Stress Scales (DASS-21). Among the seropositive participants (39 men and 24 women), both testosterone and cortisol levels were significantly elevated compared to seronegative controls. Additionally, stress and anxiety scores were higher across sexes, while depressive symptoms increased only in men. These results suggest that *Toxoplasma* infection is associated with the hyperactivation of the HPA axis, potentially contributing to mood disturbances.

Another human study focused on schizophrenic patients examined the prevalence of latent toxoplasmosis and its association with cortisol, testosterone, and glutathione (GSH) levels [305]. While no significant difference in *Toxoplasma* IgG prevalence was found between patients and controls, infected individuals—particularly males—exhibited higher cortisol and testosterone levels, alongside lower GSH concentrations. The authors argue that these changes indicate increased oxidative stress in the infected population, likely exacerbating the pathophysiology of schizophrenia and possibly other neuropsychiatric conditions.

Despite these findings, the relationship between *Toxoplasma* infection and cortisol is not fully understood and appears to be modulated by a variety of factors. Sex-specific hormonal interactions, individual variability in HPA axis responsiveness, and species-level differences all contribute to the observed heterogeneity [306,307]. Moreover, neuroimmune interactions—particularly those affecting glucocorticoid receptor sensitivity—may further complicate this picture [308]. For instance, chronic inflammation may desensitize glucocorticoid feedback mechanisms, leading to the dysregulation of cortisol rhythms and altered health and behavioral outcomes.

Taken together, these findings suggest that *Toxoplasma* may influence cortisol levels in both animals and humans, with implications for stress responsivity, immune function, and mental health. However, the direction and magnitude of this effect depend on complex interactions among host sex, infection duration, genetic background, and environmental context. As such, cortisol represents a critical but context-dependent factor in the neuroendocrine profile of *Toxoplasma*-infected individuals.

### 7.3. The Immune System and Toxoplasmosis

The interplay between *Toxoplasma* infection and the host immune system has been widely studied. Early animal models, such as those infecting SWR/J mice with the Me49 strain, revealed signs of acute immune activation—lymphoid clusters, thymic atrophy, and hepatic inflammation—followed by a resolution phase [309]. Other studies demonstrated that *Toxoplasma* triggers astroglial activation via IL-1, causing localized brain inflammation [310].

Human studies have also revealed significant immunological responses to *Toxoplasma*. During the acute stage of the disease, infected individuals exhibit altered concentrations of various cytokines. For instance, one study found that infected women had approximately double the levels of IL-6 (a pro-inflammatory cytokine) and IL-5 (a Th2 cytokine), and five-fold higher levels of IL-10 (an anti-inflammatory cytokine) compared to uninfected controls [311]. In contrast, key Th1 cytokines such as IL-12 and tumor necrosis factor-alpha (TNF-α) did not differ significantly from controls in that study, suggesting a shift towards a humoral/anti-inflammatory response during the chronic phase of infection.

Recent studies indicate that pronounced changes in cytokine levels and other molecules involved in immune processes, particularly inflammatory responses, are also evident during the latent stage of toxoplasmosis. Markers of persistent immune activation have been observed; for instance, among older adults, higher anti-*Toxoplasma* antibody titers are associated with increased indoleamine 2,3-dioxygenase activity (evidenced by an elevated kynurenine-to-tryptophan ratio) and higher levels of soluble TNF receptor II, suggesting ongoing inflammatory signaling [312]. Biomarkers such as soluble vascular cell adhesion molecule-1 and C-reactive protein were also found at elevated levels in infected individuals, suggesting systemic inflammation even after controlling for demographic factors [33]. In pregnant women, infection also correlated with increased cytokines like IL-1β, IL-1ra, IL-2, FGF-basic, and PDGF-BB [313]. Another recent study performed on pregnant women found increased levels of TNF, IL-6, and IL-10, and decreased levels of IL-8 and MIF; however, these changes were present only in women with depressive disorder [314].

Individuals with *Toxoplasma* infection exhibit altered immune profiles: men showed reduced NK cells, monocytes, and leukocytes, while women had increased counts; both sexes showed a decline in B cells [315]. These alterations suggest that chronic *Toxoplasma* infection can have an immunosuppressive effect in men (lower innate immune cells) while possibly stimulating leukopoiesis in women—a difference that remains unexplained but may relate to hormonal or genetic factors. The same study noted that the differences diminished as time since infection increased (as inferred from declining antibody titers), implying that the immune impact is strongest in the earlier phases of latent infection. Notably, the authors also observed an unusually low prevalence of *Toxoplasma* infection among men with certain immune-mediated illnesses in their clinic population, hinting that *Toxoplasma*’s immunosuppressive effects might protect against some immunopathological conditions in males. This aligns with the hypothesis that chronic *Toxoplasma* infection induces a mild downregulation of immunity (e.g., via IL-10 and other regulators) that could, in theory, reduce risk for disorders driven by overactive immune responses (autoimmunity, allergies).

Overall, the cytokine profile in latent toxoplasmosis often shows simultaneous pro- and anti-inflammatory signals, reflecting the host–parasite balance. The immune system is active enough to restrain the parasite (with IFN-γ, IL-12, etc.) yet modulated by IL-10, IL-4, and other factors to avoid excessive inflammation which inadvertently may aid parasite persistence [316].

#### 7.3.1. Immune-Mediated Physical Health Effects

The immune response to *Toxoplasma* begins with recognition by macrophages and dendritic cells, which triggers pro-inflammatory cytokine production (e.g., TNF-α, IL-12) and activates NK and T cells. IFN-γ plays a central role, inducing antiparasitic mechanisms like nitric oxide production and vacuole destruction via Immunity-Related GTPases (IRGs) and Immunity-Related GTPases (GBPs) [317,318]. While crucial for controlling the parasite, this immune activity can also lead to chronic inflammation and tissue damage.

Persistent infection and the attributed immune reaction have been generally associated with a wide range of health conditions including neurological disorders [319], cardiovascular [320], kidney [321], and liver diseases [322], as well as colitis [323]. There is also emerging evidence for a link between toxoplasmosis and autoimmune conditions (e.g., rheumatoid arthritis, systemic sclerosis) through mechanisms such as molecular mimicry [97,324,325].

Metabolic consequences of infection have also been reported, with cytokine shifts potentially contributing to obesity and type 2 diabetes risk [326,327,328]. Chronic inflammation thus generally emerges likely as a central mechanism through which *Toxoplasma* can impact physical health.

#### 7.3.2. Immune-Mediated Mental Health Effects

Inflammation has been increasingly recognized as a contributing factor in various mental health disorders, including schizophrenia, bipolar disorder, OCD, depression, and anxiety [329,330,331,332,333,334,335]. In addition, elevated levels of inflammatory markers such as interferon-gamma have been associated with reduced attention and poorer cognitive performance in healthy older adults [336]. *Toxoplasma* infection has also been linked to psychiatric disorders (see Section 5), and individuals infected with *Toxoplasma* have shown impairments in memory, processing speed, and executive function [112]. A likely key mechanism underlying these effects appears to be the host’s immune-mediated inflammatory response to the parasite. Consequently, many of the cognitive and psychiatric disturbances associated with toxoplasmosis may be, at least in part, driven by inflammation triggered by the immune response.

#### 7.3.3. Immune-Mediated Reproductive Effects

*Toxoplasma*-induced immune changes may also underlie some reproductive anomalies. According to the immunosuppression hypothesis, reduced immune rejection increases the survival of more immunogenic male or defective embryos, possibly explaining phenomena like altered sex ratios and prolonged pregnancies in infected individuals [207], as already mentioned earlier in this review, as can be seen in Section 6.2. The immunosuppression hypothesis may also account for the earlier findings of a remarkably high prevalence of chronic *Toxoplasma* infection among mothers of children with Down syndrome (84% vs. 32% in controls) [337], as well as for observations of a smaller fetal size at the first ultrasonography and prolonged pregnancy in *Toxoplasma*-infected women [198]. It has been suggested that, due to infection-induced immunosuppression and the resulting reduction in the stringency of quality-control mechanisms during early pregnancy, *Toxoplasma*-infected mothers may be more likely to carry fetuses with developmental abnormalities to term [198].

In experimental mice, latent infection led to lower IL-10 and nitric oxide production, and reduced lymphocyte proliferation, all consistent with a suppressed immune profile [338]. These findings suggest that immune modulation by *Toxoplasma* may contribute to subtle shifts in reproductive physiology that likely align with the broader patterns of host manipulation observed in latent infection (see Section 3 in this review).

For a summary of the findings of this chapter, see Table 7.

## 8. Host and Parasite Factors Modulating the Effects of *Toxoplasma* Infection

The effects of latent *Toxoplasma* infection on hosts are influenced by several critical factors specific to both the host and the parasite. Key factors include biological sex, RhD status, and the strain of the parasite, all of which play a significant role in modulating these effects. These factors warrant special attention in both clinical and behavioral contexts related to physical and mental health. It is important to note that animal and tissue model studies typically examine post-acute *Toxoplasma* infection, rather than the truly latent or chronic form observed in humans, due to the specific design and timing of these experiments.

### 8.1. Modifying Effect of Host Sex on Toxoplasma-Associated Behavioral and Health Changes

Biological sex significantly moderates the behavioral, cognitive, mental health, and physical health effects associated with many environmental and biological factors. Some of the observed sex-dependent differences may partly result from distinct immune and endocrine profiles: testosterone in males tends to suppress immunity, whereas estrogen in females may enhance cell-mediated immune responses [107]. These physiological variations, as well as the differences in the progression of infection that arise from them, could influence not only general health outcomes but also the course of latent infections, including toxoplasmosis, and thereby contribute to differences in how the infection manifests in male and female hosts.

*Toxoplasma* has been shown to modify behavior, neuropsychological traits, and even physical condition in both animal models and humans. Emerging evidence suggests that the biological sex of the host plays a critical role in modulating these effects. Many earlier studies focused exclusively on males (e.g., male rodents) or, in human research, pooled data from men and women, potentially obscuring important sex-specific outcomes. However, studies that have analyzed males and females separately indicate that *Toxoplasma*-induced changes often differ between the sexes—sometimes affecting entirely different traits, and in humans frequently influencing the same traits in opposite directions. These sex-specific effects and their potential underlying mechanisms will be discussed in the following sections.

#### 8.1.1. Animal Studies

Only a minority of animal studies have examined *Toxoplasma*-associated ethological changes separately in males and females. Hay et al. [12] reported a significant toxoplasmosis–sex interaction effect on mouse activity; however, this effect was likely driven by a subgroup of congenitally infected rather than postnatally infected mice. In both groups, *Toxoplasma*-infected males and females were more active in the open field test compared to their uninfected controls.

Another study was conducted on genetically uniform F1 hybrids (offspring of BALB/c females and B10.A males), which are highly resistant to the pathogenic symptoms of toxoplasmosis. Skallová et al. [41] investigated behavioral differences between infected and uninfected males and females in the open field test and the holeboard test, both under normal conditions and after the administration of the dopamine reuptake inhibitor GBR 12909. Their results showed that, in both sexes, *Toxoplasma*-infected hybrids exhibited decreased locomotor activity in the open field. However, infected males entered the most exposed central area of the arena more frequently than controls, whereas infected females entered it less often. Moreover, only infected females displayed increased exploratory behavior in the holeboard test. The administration of GBR 12909 suppressed exploratory activity in infected males but enhanced it in uninfected male controls.

Sex-specific behavioral differences observed in *Toxoplasma*-infected hosts may, at least in part, be explained by differences in the course and severity of infection between males and females. Empirical data clearly demonstrate that the progression of toxoplasmosis and the associated immune processes differ between male and female rodents. For example, Hegazy et al. [339] found that female mice exhibited a higher brain cyst burden and more severe pathological changes compared to males, while male mice showed significantly elevated IL-12 levels, indicating a stronger immune response.

In some cases, male and female hosts exhibit similar behavioral responses to *Toxoplasma* infection, although the underlying mechanisms differ between sexes. The striking “fatal attraction” phenomenon—the loss of innate fear of cat odors and even attraction to these odors [45] —was for years typically investigated only in male animals, or the sex of the animals was not explicitly specified. More recent work confirms that female rodents can likewise exhibit this parasite-induced behavioral change [206]. For example, infected female rats showed a marked reduction in aversion to cat urine, similar to that observed in males. However, the underlying mechanisms appear to differ by sex. In male rats, latent infection leads to endocrine and neurobiological changes—including significantly elevated testosterone levels and altered arginine–vasopressin signaling in the medial amygdala—that are causally linked to the loss of predator fear [278]. In contrast, infected female rats also lose their fear of cat odor without any detectable changes in gonadal steroid levels, and ovariectomy does not prevent the behavioral change. This suggests that females rely on a different, yet unidentified, pathway to achieve the same behavioral outcome, rather than the testosterone-mediated mechanism observed in males. Although both sexes are behaviorally susceptible to latent *Toxoplasma* infection, the proximate physiological processes appear to be sex-specific.

In conclusion, it is highly probable that sex-based immune and hormonal differences in rodents—such as the contrasting immunomodulatory effects of testosterone and estrogen—modulate parasite load or neurophysiological outcomes. Although only a limited number of studies have investigated males and females separately, available evidence suggests that host sex can influence not only the magnitude of *Toxoplasma*’s behavioral manipulation but also its underlying biological mechanisms. These findings underscore the critical importance of including both males and females in experimental designs to better capture the full complexity of host–parasite interactions.

#### 8.1.2. Human Studies

Early studies [19,20] highlighted opposite shifts in personality traits measured with Cattel’s 16PF questionnaire. For instance, they reported decreases in extraversion, cooperativeness, and superego strength (rule-conscious behavior) in infected men but increases in those traits among infected women. Lindová et al. [71] later found similar general trends using a different personality questionnaire (NEO-PI-R), noting increased extraversion and reduced conscientiousness across both sexes, yet specific traits like self-esteem and interpersonal dynamics still differed.

A double-blind ethological study by Lindová et al. found that latent *Toxoplasma* infection affected composite behavioral traits analogously to personality factors, with significant sex interactions [54]. Infected men scored markedly lower on self-control and conscientiousness-related measures than uninfected men, whereas infected women showed a slight increase in those same measures (an opposite trend). Similarly, infected men were rated lower in warmth (friendliness) compared to uninfected men, while no decrease was observed in women.

Behaviorally, Cook et al. [55] reported heightened reactive aggression predominantly in infected women, while infected men aged 20–59 years exhibited increased impulsive sensation-seeking. In economic decision-making tasks, a study found sex-opposite changes consistent with stress-coping differences: infected men behaved more impulsively and selfishly in money-allocation games, whereas infected women were more inclined toward cautious or cooperative choices [75].

Across these personality and behavioral measures, the trend is that *Toxoplasma*-infected males tend toward lower sociality, lower conscientious restraint, and higher risk acceptance, while females tend toward heightened social affiliation and often greater rule-consciousness or harm avoidance—effectively mirror-image responses. Possible explanations for the opposite effects of toxoplasmosis on various personality and behavioral traits—including the currently best-supported stress-coping hypothesis—are discussed in detail in Section 2.2.3.

Regarding cognitive impacts, *Toxoplasma* infection produces context-dependent effects. In general, regardless of sex, some studies report enhanced performance in challenging cognitive control tasks among infected individuals [224], while others indicate cognitive impairments, such as reduced immediate memory [340] and decreased psychomotor performance [110]. However, recent research has emphasized sex-specific outcomes; for example, infected women showed impaired working memory performance and increased response times in N-Back tasks—which evaluate working memory capacity—but not in the Simon task, which examines how spatial cues can interfere with cognitive processes such as memory [341]. Another large study found significant cognitive deficits, including reduced IQ scores, with effects generally stronger in *Toxoplasma*-infected men [109]. However, in this study, the interaction between sex and infection reached statistical significance for human cytomegalovirus but not for toxoplasmosis. Still, the findings underscore the importance of considering sex differences in future cognitive research on the effects of pathogens. Further evidence for the sex-specific effects of latent toxoplasmosis comes from an analysis of UK Biobank data, which found that the relationship between anti-*Toxoplasma* antibody titers and performance on a digit-symbol substitution test—a measure of processing speed and working memory—differed significantly between men and women [122]. In men, higher levels of anti-*Toxoplasma* antibodies were linked to a poorer performance on the symbol-digit substitution test, whereas in women, higher antibody levels were associated with better performance on the same task.

While details varied, a general pattern was that male performance tended to be more adversely affected on tasks requiring rapid cognitive processing or sustained attention. This aligns with the idea that infected men’s slight psychomotor slowing (as seen in reaction time studies) could impact cognitively demanding activities, whereas infected women might be relatively spared or show smaller effects on these particular functions. It must be noted that not all studies find significant cognitive impairment, and those that do, often reveal small effect sizes. Nonetheless, considering sex as a moderating factor is crucial—pooled analyses might miss effects that are present in one sex but diluted by the other.

Perhaps the most consequential domain of sex-specific effects is mental health. A cross-sectional study performed on 3800 internet users showed that *Toxoplasma* infection was associated with higher odds of autism (odds ratio = 7.83 vs. 1.23), schizophrenia (odds ratio = 3.70 vs. 2.82), learning disabilities (odds ratio = 2.29 vs. 1.29), antisocial personality disorder (odds ratio = 2.26 vs. 1.39), and attention deficit hyperactivity disorder (odds ratio = 3.50 vs. 2.00) compared to uninfected individuals [148]. Another large study of approximately 1400 individuals with alcohol consumption habits found that women with anti-*Toxoplasma* antibodies had a significantly higher incidence of self-harm and suicide attempts compared to uninfected women (nearly 1.5–2 times higher odds) [342]. In men, however, toxoplasmosis was not significantly associated with suicide attempts in the same analysis.

Hlaváčová et al. [343] found infected women without fertility issues had higher depression scores, whereas infected men facing infertility had lower depression scores than their uninfected peers. Another recent study examining a broad range of psychopathological symptoms (using the SCL-90 questionnaire) reported that infected men scored higher on Interpersonal Sensitivity and Psychoticism scales (indicating greater social insecurity and mild paranoid or hostile tendencies), whereas infected women did not significantly differ from controls on those dimensions [344]. Similarly, recent evidence, comparing infected men with infected women, indicates that the duration of infection significantly predicts mental health issues such as depression and schizophrenia in infected men but not women [173], emphasizing the critical role sex plays in psychiatric outcomes of toxoplasmosis.

A large population-based study using data from the NHANES (2009–2014) examined the association between *Toxoplasma* infection and cardiovascular disease (CVD) mortality. Analysis of over 10,000 individuals revealed that *Toxoplasma* seropositivity was linked to a 40% higher risk of CVD mortality in men, but no significant association was found in women [345]. Although studies have explored sex-specific differences in latent toxoplasmosis, further research is needed to directly compare physical health outcomes between men and women. In this respect, stratified analyses in large, diverse cohorts, alongside longitudinal studies investigating sex hormones, inflammatory pathways, and endothelial function in the context of infection, are essential to determine whether *Toxoplasma* infection contributes to distinct health risks or disease trajectories in each sex.

### 8.2. Rhesus Factor

The Rh factor—specifically the presence or absence of the D antigen, which is encoded by the RHD gene and expressed on the RhD protein located on the surface of red blood cells [346,347]—is primarily known for its role in determining blood type. However, it is important to note that Rh proteins, likely including RhD, are not exclusive to red blood cells. They are also found on other cells in the body, including some cells in the brain [348]. Although the role of Rh in human physiology remains vastly unknown, studies during the past two decades suggest that it has important roles to play in the individual’s response to various adverse factors, including *Toxoplasma* infection.

For example, an early study, performed on a cohort of 553 male and female blood donors in three different hospitals and 464 male conscripts, investigated the possible protective role of RhD heterozygosity against *Toxoplasma*-induced negative effects on the infected individuals’ reaction times [114,115]. The authors found that RhD-negative subjects were more susceptible to the adverse effects of latent toxoplasmosis on simple reaction times compared to RhD-positive heterozygotes. Due to the low number of female participants, effects were significant only in men. However, another study using the same experimental setup to examine the effects of RhD phenotype and toxoplasmosis on simple reaction times in a sample of 110 male and 226 female students found significant RhD–toxoplasmosis interaction effects in female students [115].

Another study [72] examining the differences in the effects of toxoplasmosis between RhD-positive and RhD-negative individuals found that infected RhD-positive participants scored lower on intelligence measures than uninfected controls, whereas infected RhD-negative individuals showed higher scores. The researchers discovered that the noted variances related to *Toxoplasma* were consistently less pronounced in individuals who were RhD-positive compared to those who were RhD-negative. The existence of a modifying effect of the RhD phenotype on the impact of toxoplasmosis on human performance was also confirmed by the results of a prospective case–control study conducted on a population of 3890 military drivers [113]. *Toxoplasma*-infected drivers (29.7% of all participants) experienced more traffic accidents during their 1–1.5-year compulsory military service (odds ratio = 2.56, 95% CI: 1.14–5.76, t = 2.287, *p* = 0.022); however, separate analyses according to RhD phenotype revealed that this effect was significant only in RhD-negative individuals (odds ratio = 2.53, 95% CI: 1.12–5.70, *p* = 0.026).

The RhD phenotype also modifies other effects of *Toxoplasma* infection. The strongest effect reported to date concerns weight gain during the 16th week of pregnancy [199]. Among 27 RhD-negative, *Toxoplasma*-infected women, the mean weight gain was 4.12 kg, compared to 2.44 kg in 127 RhD-negative, *Toxoplasma*-free women, 2.65 kg in 590 RhD-positive, *Toxoplasma*-free women, and 2.35 kg in 158 RhD-positive, *Toxoplasma*-infected women. This difference (about 1.6 kg) remained approximately constant until the end of pregnancy.

Another study demonstrated that the RhD phenotype modifies the effect of toxoplasmosis on physical endurance performance [349]. RhD-negative, *Toxoplasma*-infected students showed significantly poorer performance in the weight-holding and hand grip tests. In contrast, among RhD-positive students, infected individuals performed better than their uninfected counterparts. The study also discussed that similar paradoxical improvements in performance among *Toxoplasma*-infected, RhD-positive individuals have been observed in several other studies. This pattern may reflect an evolutionary context in which our African ancestors were likely universally or nearly universally infected with *Toxoplasma* and predominantly RhD-positive, potentially shaping human physiology and cognition to function optimally under such conditions.

Subsequent research confirmed these patterns. Most studies to date have analyzed the modifying effects of the RhD phenotype rather than the RhD genotype. However, a few studies have calculated or experimentally determined the RhD genotypes of participants. These studies showed that the effects of toxoplasmosis differ significantly depending on whether an RhD-positive individual is homozygous or heterozygous for the RHD gene. The first evidence suggesting a protective role of RhD heterozygosity was provided by a study published in 2008, which showed that *Toxoplasma*-infected RhD-positive heterozygotes exhibited significantly better (i.e., shorter) simple reaction times compared to *Toxoplasma*-infected, RhD-positive or RhD-negative homozygotes [114]. In this study, conducted on blood donors, complete Rh phenotypes (D, C, and E antigens) were available, allowing researchers to estimate homozygosity or heterozygosity based on the known population frequencies of genotypes corresponding to each phenotype (for example, the D+C+c–E–e+ phenotype is encoded in 95.5% of cases by DD homozygotes and in 4.5% by Dd heterozygotes [350]).

Four additional studies did not differentiate between *Toxoplasma*-infected and uninfected individuals but instead examined the protective effect of RhD heterozygosity in the general population. Given the high prevalence of *Toxoplasma* infection in the general population, it can be assumed that the observed effects are at least partially related to protection against the negative impacts of toxoplasmosis.

The first study, this time conducted on a cohort of 2539 individuals, identified RHD heterozygotes based on the reported RhD phenotypes of the participants and their biological parents [351]. This study found that RhD-negative homozygotes scored significantly worse in both physical and mental health measures compared to individuals with an RhD-positive phenotype. Moreover, RhD-positive heterozygotes reported better health outcomes than both RhD-positive and RhD-negative homozygotes. Interestingly, RhD-positive homozygotes appeared to experience poorer health than RhD-negative homozygotes.

The second study employed molecular genotyping (using the rtPCR method) to distinguish RhD-positive heterozygotes from homozygotes [352]. Conducted on 178 women and 86 men, this study found a significant RhD–sex interaction effect on physical (but not mental) health. RhD-positive homozygous women reported poorer physical health than both RhD-negative and heterozygous women, whereas among men, RhD-negative individuals reported worse physical health than both RhD-positive homozygotes and heterozygotes. This interaction remained significant even after correction for multiple comparisons (*p* = 0.019). No significant differences in physical health were observed between RhD-negative homozygotes and RhD-positive heterozygotes.

The third study investigating the effects of RHD genotypes was an ecological regression analysis testing the hypothesis that Rhesus factor polymorphism is maintained by heterozygote advantage [353]. This study analyzed WHO disease burden data, including mortality and Disability-Adjusted Life Years (DALYs), from 65 countries where data on the frequency of RhD-negative individuals were available. These incidences were used to estimate the frequency of RhD heterozygotes based on the Hardy–Weinberg equilibrium calculations. The results revealed a significant positive correlation between national disease burden and the frequency of RhD-negative individuals, and a significant negative correlation between disease burden and the frequency of RhD-positive heterozygotes. A multivariate GLM model, accounting for several potential confounding factors, showed that the frequency of RhD heterozygotes (μ^2^ = 0.67, *p* = 0.013), frequency of smokers (μ^2^ = 0.75, *p* = 0.001), latitude (μ^2^ = 0.71, *p* = 0.005), and humidity (μ^2^ = 0.64, *p* = 0.027) were significant predictors of national disease burden. In contrast, the frequency of RhD-negative homozygotes (μ^2^ = 0.60, *p* = 0.070), GDP (μ^2^ = 0.42, *p* = 0.635), and medical care expenditure (μ^2^ = 0.23, *p* = 0.991) were not significant. Post hoc analyses revealed that the frequency of RhD-negative individuals correlated significantly with DALYs for 21 out of 121 diseases or disease categories, while the frequency of RhD-positive heterozygotes correlated with DALYs for 25 of the 121 diseases or categories for which DALY data were available. Some of these correlations were particularly strong and concerned major diseases. For instance, the frequency of RhD-negative inhabitants explained 32% of the between-country variance in ischemic heart disease-related mortality, 37% of the variance in malignant neoplasm-related mortality, and 42% of the variance in Parkinson’s disease-related mortality [353].

The last study conducted on 3130 individuals demonstrated that RhD-negative subjects scored significantly worse than RhD-positive individuals in 6 of 22 health-related indices and RhD-negativity was significantly associated, mostly positive, with incidence in 31 of 154 diseases reported by at least 10 participants [354]. For example, RhD-negative participants reported higher frequencies of allergic, digestive, cardiovascular, hematological, immune, mental health, and neurological problems. Another example comes from a large-scale internet study involving 1768 male (24% RhD-negative) and 3759 female participants (23% RhD-negative), which revealed that RhD–sex interaction, rather than the RhD phenotype alone, may influence health and well-being [355]. Specifically, RhD-negative men scored lower in measures of mental health, whereas RhD-negative women scored higher in measures of physical health. Moreover, lower well-being scores were observed only among RhD-negative women, not men. The RhD-negative individuals of both sexes reported engaging in more frequent sexual activity compared to their RhD-positive counterparts. The authors interpreted this finding as indirect evidence for a shift toward a fast life-history strategy. Such a shift is considered an evolutionary adaptation in individuals experiencing poorer health, promoting earlier or increased reproductive effort while their physical condition still permits successful reproduction [77].

As already mentioned, given the high prevalence of latent toxoplasmosis in the general population, it is possible that the observed associations of the RhD phenotype or genotype with physical and mental health are driven by the greater resistance of RhD-positive heterozygotes to the adverse effects of *Toxoplasma* infection. However, there is also strong evidence that RhD positivity—and more specifically, RhD heterozygosity—may confer protection not only against the negative effects of *Toxoplasma* infection but also against other adverse influences. For example, the results of a cross-sectional study involving 302 blood donors indicated that the personality profiles of RhD-positive and RhD-negative individuals responded not only differently to *Toxoplasma* infection but also to the effects of aging [70]. In RhD-positive participants, dominance decreased and shrewdness increased with age, whereas in RhD-negative individuals, these associations were reversed. The age–RhD factor interactions were statistically significant for both dominance (*p* = 0.006) and shrewdness (*p* = 0.014). Similar findings were reported in another cross-sectional study conducted on 3800 military draftees [356]. This study suggested that the RhD phenotype may modulate not only the effects of age on personality traits, but also its influence on health and performance in tests assessing attention, short-term memory, and intelligence. In contrast to the previous study on blood donors (mean age 35.3 years, range 18–64), the effects of age were stronger in RhD-positive than in RhD-negative individuals in this younger cohort of draftees (mean age 19.8 years, range 17–31). The authors proposed that the two studies captured the effects of different age-related processes—namely, senescence in the older sample and maturation in the younger one.

RhD positive individuals were also more resistant to the negative effects of smoking. Already discussed cross-sectional study performed on 3800 draftees showed the adverse effect of smoking on the number of viral and bacterial diseases was about three times stronger for RhD-negative than RhD-positive subjects [356]. In the same time, the association between smoking and 11 of the 23 personality traits measured by Cattell’s 16PF and Cloninger’s TCI tests were much stronger in RhD-positive than RhD-negative participants.

**Figure 9:**
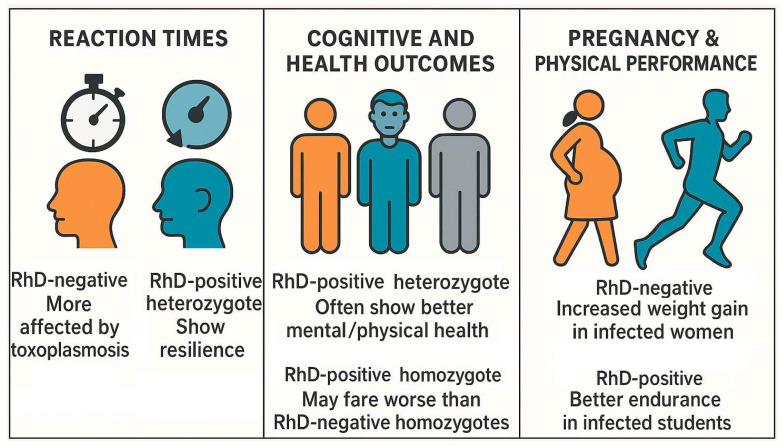
Interaction between RhD status and toxoplasmosis across cognitive and physical domains.

In conclusion, findings on the role of RhD phenotype and genotype suggest that RhD status has a substantial and possibly underestimated impact on human health and resilience to adverse factors. Research on the behavioral and health effects of chronic *Toxoplasma* infection led to the discovery that RhD heterozygosity may confer protection against certain adverse outcomes associated with infection. Subsequent studies suggested that the impact of RhD phenotype and genotype extends beyond toxoplasmosis, influencing various aspects of physical and mental health. The effects of RhD status appear to be both common and relatively strong. They are also likely even stronger than currently estimated, as most existing studies have simply compared RhD-negative and RhD-positive individuals. However, growing evidence indicates that RhD-positive homozygotes and heterozygotes differ substantially in their susceptibility to adverse effects. In particular, RhD-positive homozygotes may not be protected at all. In fact, some studies even suggest that RhD-positive homozygotes may fare worse than RhD-negative homozygotes. This discrepancy suggests that previous analyses may have masked important genotype-specific effects (See Figure 9).

Although the body of evidence reviewed in this section suggests that the RhD genotype could have considerable implications for public health, research in this area remains extremely limited. As far as we know, since 2008—when the first studies on the influence of RhD genotype on psychomotor performance were published— only a single independent study from a different laboratory—not primarily focused on this question—has provided relevant data, and it failed to confirm a protective effect of RhD positivity on patient survival after blood transfusion [357]. Given the potential importance of these findings, systematic independent replication and further investigation are urgently needed. Future research should include both population-based and experimental studies designed to examine the effects of RhD genotype on a range of physiological, cognitive, and health-related outcomes. Large-scale cohort studies could assess associations between RhD status and disease susceptibility, neurocognitive function, and treatment outcomes, while accounting for possible confounders such as age and sex. In addition, experimental approaches may help uncover underlying mechanisms, such as immune modulation, oxidative stress resistance, or neural signaling differences, associated with RhD expression in infected individuals.

### 8.3. Strain of the Parasite

The biological properties of different *Toxoplasma gondii* strains vary dramatically [358]. Extensive research has shown that parasite genotypes differ in virulence, replication rate, tissue tropism, immune modulation, and capacity for cyst formation. These differences have been most thoroughly studied in the context of acute toxoplasmosis and its clinical manifestations e.g., [359]. However, it is highly likely that strain-specific differences also influence the long-term consequences of *Toxoplasma* infection, including effects on host behavior, cognitive performance, mental health, and physical health. Given the profound strain-dependent variation observed in acute infection and in animal models of chronic infection, the parasite’s genotype must be considered a factor potentially shaping the nature and severity of *Toxoplasma* infection outcomes in humans.

#### 8.3.1. Strain-Dependent Effects in Animal Models

Research on rodents has revealed that the behavioral and cognitive impact of *Toxoplasma* infection can vary markedly depending on the parasite’s strain (genotype). In a direct comparison of two closely related Type II strains (ME49 vs. Prugniaud), Kannan et al. [360] found divergent outcomes in mice despite only ~0.5% genomic difference. Both strains caused the classic loss of feline odor aversion in mice at 2 months post-infection, but only Prugniaud-infected mice retained this “fatal attraction” effect by 7 months. Prugniaud infection also led to hyperactivity and increased body weight, whereas ME49 infection impaired spatial memory. Notably, serum antibody titers were similar, indicating that the differing behaviors were not due to gross differences in infection intensity. This experiment demonstrates that even within the same lineage, minor genetic differences can translate into distinct neurobehavioral outcomes.

Other rodent studies reinforce the role of the parasite genotype. Some behavioral changes appear strain-general: for example, the loss of the innate fear of cat odors is observed after chronic infection with the representative strains of all three major clonal lineages (Types I, II, and III). In one study, mice infected with an attenuated Type I strain showed persistent loss of cat-urine aversion long after the parasite was cleared and brain inflammation subsided [361]. This suggests the fearlessness phenotype can be “locked in” during acute infection, regardless of strain, possibly via lasting neurochemical or circuit alterations. However, other outcomes are strain-specific. For instance, one report noted that only ME49 (Type II), and not the genetically similar Pru strain, induced memory deficits in mice [360]. Likewise, the cognitive impacts of chronic *Toxoplasma* infection vary: a recent study using a Chinese Type #1 strain (TgCtwh6) found that infected mice developed significant learning and memory impairments alongside Alzheimer’s-like neuropathology (e.g., elevated amyloid-beta plaques and neuron loss) [362]. Conversely, infection with certain Type II strains has been shown to protect against Alzheimer-like pathology in mouse models. Jung et al. [363] reported that chronic Type II infection prevented neuronal degeneration and memory impairment in transgenic Alzheimer’s mice. Follow-up studies attributed this to an infection-induced anti-inflammatory milieu that promoted amyloid-beta clearance. Intriguingly, this protective effect was strain-dependent. Cabral et al. [364] found that a Type II strain reduced amyloid-β deposition, whereas a Type III strain did not. Together, such findings highlight that rodent outcomes can differ drastically by *Toxoplasma gondii* strain—from exacerbating cognitive decline to paradoxically mitigating it—likely reflecting the differences in parasite–host immune interactions.

It is important to note that host factors also interact with the parasite strain. Highly virulent Type I strains typically cause acute lethal infection in standard inbred mice, precluding chronic behavioral studies. By using outbred mice, however, researchers have managed to establish chronic infections with a virulent Type I strain (GT1), revealing variable outcomes: some mice succumbed or cleared the infection, while survivors with higher brain cyst loads showed pronounced behavior changes [365]. In general, more neurotropic, cyst-forming strains (e.g., Type II) establish long-term brain infections and robust neuroinflammation, which tend to produce measurable behavioral alterations. In contrast, less cystogenic strains or those provoking minimal brain inflammation may lead to subtler effects [366,367]. Even the distribution of cysts in the brain can matter—one study noted that variations in where cysts concentrate in the rodent forebrain correlated with individual differences in anxiety and predator odor response [368]. Overall, animal models make clear that parasite genotype is a key variable determining the nature and magnitude of latent toxoplasmosis effects on the host’s behavior and cognition.

However, it should be again noted that, although often referred to as ‘latent’ toxoplasmosis, *Toxoplasma* infection in rodents, particularly in mice, may not be immunologically quiescent. Instead, this stage may better reflect a persistent, low-grade, post-acute phase, with ongoing neuroinflammation and active cyst presence, and as such may not fully correspond to the human *Toxoplasma* infection.

#### 8.3.2. Strain-Dependent Effects in Humans

In humans, direct evidence for strain-specific effects on behavior and mental health is more limited, largely due to challenges in genotyping infections outside of severe cases. Epidemiological hints nonetheless suggest that parasite strain influences clinical outcomes. Notably, geographical comparisons find more severe manifestations of toxoplasmosis in regions where non-Type II strains predominate. For example, South America harbors highly diverse *Toxoplasma* populations, and infection there is associated with unusually frequent ocular and systemic complications in immunocompetent people [369]. A cross-sectional study in Colombia and France demonstrated that Colombian patients (infected with atypical and Type I strains) had significantly more severe ocular toxoplasmosis than French patients (infected mostly with Type II strains). The South American cases showed larger retinal lesions, greater inflammation, and worse vision loss, linked immunologically to a dampened intraocular IFN-γ response relative to the French cases [370]. These findings support that atypical and recombinant *Toxoplasma* strains are linked to more severe disease outcomes, especially in immunocompromised patients such as those with HIV or organ transplants [371,372,373,374]. While most cases had poor prognoses, milder outcomes have also been reported with type II strain reactivation [375].

When it comes to subtler behavioral or psychiatric effects in humans, the data are more circumstantial but provocative. One notable study applied serological strain-typing in a cohort of U.S. mothers to investigate links between latent toxoplasmosis and adult psychosis in their offspring. Remarkably, only maternal infections consistent with Type I strain exposure were associated with a significantly increased risk of psychoses in the adult children [376]. The offspring of mothers harboring Type I infections had elevated odds of developing psychoses, whereas maternal infections by Type II or III strains showed no such association. Another study of *Toxoplasma*–positive pregnant women reported higher rates of depression and anxiety symptoms in the infected mothers, with the highest mood disturbance scores in those serotyped as Type I infections, though this trend did not reach statistical significance in that sample [165]. These studies are limited in size, but they lend support to a “strain hypothesis” in human toxoplasmosis—i.e., that certain genotypes (possibly more neurovirulent or inflammation-inducing) may carry greater risk for psychiatric outcomes.

Critical gaps remain because most human studies on *Toxoplasma* and neuropsychiatric disease do not determine the infecting strain. *Toxoplasma* infection in people is typically identified by serology alone, and genotyping the parasite would require tissue biopsy or DNA from sites like brain or eye, which is infeasible without an acute complication. This means we largely extrapolate from geographic strain prevalence or animal models to infer strain effects in humans. The lack of direct data is critical because, without strain identification, associations between *Toxoplasma* and disorders (e.g., schizophrenia, suicide, cognitive decline) could overlook important heterogeneity. If, for instance, only certain genotypes cause significant neural dysfunction, pooled analyses may dilute those effects and yield inconsistent results across populations. Improving our understanding here is not just academic—it could inform risk assessment and clinical management. For example, individuals infected by a highly neurotropic strain might merit closer neurological follow-up. In sum, human studies to date hint at strain-dependent differences (with Type I/atypical strains implicated in more severe ocular and possibly psychiatric outcomes) but this remains a largely understudied area demanding further research. This should focus on developing non-invasive methods to identify *Toxoplasma* strains in humans, such as advanced serotyping or circulating DNA assays. In addition, longitudinal studies incorporating strain-level data are needed to clarify associations with neuropsychiatric outcomes. Animal models and biobanked clinical samples could also help elucidate strain-specific effects, ultimately guiding risk assessment and targeted clinical monitoring.

For a summary of the findings in this chapter, see Table 8.

## 9. Conclusions

This review article provides an extensive summary of research on the impacts of *Toxoplasma* infection, particularly focusing on their implications for human health and performance. While the parasite’s manipulation of behavior, including decreased fear, weakened cognitive functions, and altered sexual behavior, has historically been a key area of study, it is now increasingly evident that many of these effects are likely secondary consequences of the parasite’s primary impact on the host’s health. The observed behavioral changes, therefore, may stem from broader alterations in physiological and cognitive functions induced by the infection, rather than being solely a result of direct parasitic manipulation.

Although so-called latent toxoplasmosis was considered harmless or even asymptomatic for decades, research over the past 30 years has revealed that chronic *Toxoplasma* infection is associated with a wide range of health issues. Increasing evidence points to elevated morbidity from cardiovascular to neurological conditions among infected individuals. Cognitive impairments—particularly in reaction time, memory, and executive function—have also been repeatedly documented, raising concerns about subtle but widespread impacts on daily functioning and performance. Given the parasite’s ability to affect neural and neurochemical pathways, it is perhaps unsurprising that latent toxoplasmosis has also been linked to psychiatric conditions such as schizophrenia, bipolar disorder, OCD, and anxiety.

The precise physiological mechanisms driving these effects are still being elucidated, but likely involve a combination of direct CNS damage, hormonal changes, neurotransmitter alterations, and immune responses. Understanding these mechanisms is crucial for developing strategies to mitigate the negative health and performance outcomes associated with *Toxoplasma* infection.

One of the important outcomes of research on “asymptomatic” latent toxoplasmosis conducted over the past 30 years has been the recognition of the need to explore effective treatment options and potential eradication strategies. Promising directions include, for instance, the development of oral vaccines suitable for use across feline populations, including domestic, feral, and wild cats. Addressing these issues is essential for improving health outcomes and mitigating the long-term consequences of this globally widespread parasitic infection.

More broadly, our findings emphasize the potential for pathogen-induced alterations in health, cognition, and behavior, effects that may be more prevalent than currently recognized. *Toxoplasma* serves as a particularly well-studied example of how a seemingly asymptomatic latent infection can exert subtle yet measurable effects on human functioning. While this study focused specifically on *Toxoplasma*, future research may benefit from investigating whether similar patterns exist in other latent or chronic infections, using both evolutionary and mechanistic frameworks.

Expanding our understanding of these effects also has important public health implications. Future efforts should consider implementing targeted screening strategies for at-risk populations, developing public education campaigns to raise awareness about subclinical toxoplasmosis, and investing in intervention approaches that can mitigate the long-term cognitive, psychiatric, psychological, physical health, and physiological impacts. Such measures would help translate academic findings into actionable health policies and preventative care practices.

## Figures and Tables

**Table 1:** Classification of human *Toxoplasma* infection forms.

Type of Toxoplasmosis	Description	Duration	Symptoms	Clinical Relevance
Acute toxoplasmosis	Recently infected individuals showing pronounced but transient symptoms of an active infection.	Days to weeks	Fever, lymphadenopathy, fatigue	Usually self-limiting in immunocompetent individuals
Subclinical (latent) toxoplasmosis	Long-term (potentially lifelong) infection with subtle, often non-specific symptoms or behavioral changes, some of which may worsen over time.	Lifelong	Mild neurological, immunological, or behavioral symptoms; elevated risk of diverse somatic and psychiatric disorders with often more severe course	Often undiagnosed; possible impact on host behavior
Chronic toxoplasmosis	Long-term (potentially lifelong) infection with persistent or recurrent clinical symptoms that may require medical treatment.	Lifelong	Chronic headaches, muscle pain, neuropsychiatric issues	May require long-term management
Congenital toxoplasmosis	Infection acquired in utero via vertical transmission from an infected mother, especially during primary maternal infection in pregnancy.	Lifelong	Visual and hearing impairments, cognitive deficits; in severe cases, hydrocephalus, chorioretinitis, and cerebral calcifications, miscarriage	High clinical importance; requires early intervention
Cerebral toxoplasmosis (toxoplasmic encephalitis)	Reactivation of latent infection in immunocompromised individuals, primarily affecting the brain.	Variable, often progressive without treatment	Headaches, seizures, focal neurological deficits, altered mental status, fever	Severe, life-threatening opportunistic infection; requires urgent medical treatment

**Table 2:** Summary of findings—*Toxoplasma*, behavior, and personality.

Topic	Key Findings	References
*Toxoplasma* and behavior in rodents	Increased activity, reduced fear of cat odors, behavioral manipulation to increase predation by cats.	[10,44]
*Toxoplasma* and behavior in non-human primates	Loss of aversion to predator urine (e.g., leopard urine in chimpanzees); strain-specific behavior changes.	[53]
*Toxoplasma* and behavior in humans	Sex-specific behavior differences, increased aggression/impulsivity, elevated risk of traffic/workplace accidents, altered decision-making.	[19,66,67]
*Toxoplasma* and personality (Cattell’s 16PF)	Sex-dependent personality shifts (e.g., lower conscientiousness in men, higher in women); interaction with RhD phenotype.	[19,70]
Personality (NEO PI-R, TCI)	Lower conscientiousness, higher extraversion, reduced novelty seeking; infection duration effects vary by test used.	[71,72,74]
Explaining opposite effects by sex	Evolutionary, physiological, and psychological explanations (e.g., stress-coping strategies, hormone modulation).	[54]

**Table 3:** Summary of findings—*Toxoplasma* and sexual behavior.

Topic	Key Findings	References
Altered sexual behavior in Infected Individuals	Infected individuals, especially men, show increased sexual promiscuity and activity; linked to elevated testosterone and dopamine levels, possibly due to parasitic manipulation.	[21,76]
Attractiveness to uninfected individuals	Infected males (rats, possibly humans) are found more attractive by uninfected females, potentially due to elevated testosterone and associated traits like dominance and symmetry.	[79]
Behavioral manipulation and predation	Infected rodents lose fear of cat odors, possibly via amygdala and limbic system changes, facilitating parasite transmission to feline hosts.	[21]
Possibility of sexual transmission	Evidence exists for sexual transmission in animals and potential in humans via semen; observed in sex workers and correlated with STD prevalence.	[81,88,89]
Qualitative changes in sexual preferences	Infected individuals report more violent or atypical sexual fantasies but engage less due to poorer health.	[95,97]
Sexual behavior of uninfected individuals toward infected	Despite typical aversion to infected mates, *Toxoplasma*-infected males may be preferred due to heightened masculine traits linked to testosterone.	[104,106]

**Table 4:** Summary of findings—*Toxoplasma* and cognition.

Topic	Key Findings	References
Cognitive outcomes (general)	Cognitive impairments include poorer working memory, reasoning, attention, and verbal fluency.	[112]
Reaction time and processing speed	*Toxoplasma*-infected individuals show slower reaction times, especially as tests progress; effects moderated by RhD phenotype.	[110,114,115]
Executive function and memory	Older adults with infection show impaired memory; middle-aged adults show impaired executive functioning; mixed findings on memory.	[32,122,123]
Youth cognition	Infected children (12–16) have lower reading/memory and effects moderated by vitamin E levels.	[124]
Psychiatric patients	In bipolar and schizophrenia patients, *Toxoplasma* associated with worse cognitive scores; antipsychotics may confound effects.	[130,131]
Alzheimer’s disease (AD)	Animal models suggest mixed effects, some studies show worsened symptoms, others show protection. Human data are inconsistent.	[116,117,119,120]
Intelligence	Mixed findings: some report lower intelligence in men, higher in RhD-negative women; recent studies suggest reduced fluid intelligence.	[20,69,72]

**Table 5:** Summary of findings—*Toxoplasma* and mental health.

Topic	Key Findings	References
Schizophrenia	Strong association, higher symptom severity and continuous illness in infected; dopamine synthesis linked to parasite	[74,86,141]
Autism spectrum disorder	Moderate to strong association, especially in men; dopamine dysfunction and prenatal immune response, infection may influence neurodevelopment via dopamine pathways	[147,148,150]
ADHD	Weak to moderate association, recent large study shows significant association, stronger in men	[31,148]
Bipolar disorder	Mixed, earlier studies suggest association, but a recent large study shows non-significant effects	[29,148,159]
Anxiety	Controversially, some studies have found association with general anxiety, whilst others have not, which is possibly stronger in infected women than infected men	[148,165,166,168]
Depression	Weak association with toxoplasmosis, no significant association with major depression but possible effect on mood	[141,148]
Obsessive-compulsive disorder (OCD)	Strong association, consistent across studies; infection linked to treatment resistance	[175,176]
Antisocial personality disorder (ASPD)	Moderate association, limited direct evidence, but personality traits and aggression linked to infection, *Toxoplasma*-seropositive men show more aggression, less conscientiousness;	[137,148]
Learning disabilities (LDs)	Moderate association, two studies show significant association with higher odds of LD	[148,175]
Substance use disorder	Mixed evidence on the link between *Toxoplasma* infection and addiction; findings vary by study and drug	[182,183,184]
Neurotropic zoonoses (*Bartonella*/*Borrelia* interaction)	Complex, co-infections with *Bartonella* or *Borrelia* may modify *Toxoplasma’s* impact on mental health (to alter risks of depression and PTSD)	[138,191]

**Table 6:** Summary of findings—*Toxoplasma* and physical health.

Topic	Key Findings	References
Ecological findings	88 countries, WHO data on 129 diseases; *Toxoplasma* seroprevalence correlated with disease burden (23/128 diseases) and mortality (12 diseases)	[27]
Cross-sectional and case-control findings	*Toxoplasma*-infected individuals even before knowledge of infection, especially in RhD-negative individuals score worse on health variables	[195]
Health → Anxiety pathway	Health deterioration mediates stress and anxiety	[26]
Pregnancy	Lower weight and longer pregnancy in infected women	[197]
Pregnancy and RhD	Higher weight gain in RhD-negative, infected women	[197]
Gestational diabetes	Higher glucose levels and gestational diabetes mellitus in infected women	[200]
Thyroid function	Mild hormonal differences, potential for autoimmune insight in pregnant women	[201]
Human infant development	Greater weight gain, delayed motor milestones	[202]
Sex ratio in both humans and animal studies	Acute infection associated with higher male ratio; chronic infection associated with lower male births	[204,205]
Men’s fertility	Lower sperm quality, higher subfertility risk	[214,215]
Women’s fertility	Older age, longer to conceive, higher assisted reproductive technology use	[217,218]
Miscarriage risk in pregnant women	Chronic infection nearly doubled miscarriage risk	[97,218]

**Table 7:** Summary of findings—biology of the mechanisms underlying the effects of *Toxoplasma*.

Category	Mechanism	Key Findings	References
Neurotransmitters	Dopamine	Increased dopamine production; linked to behavioral changes, reduced reward sensitivity, altered cognition, and mental disorders like schizophrenia.	[39,224,229,231]
	Glutamate and GABA	Altered glutamate and GABA levels; associated with cognitive impairments, psychiatric disorders (e.g., OCD, schizophrenia), and seizures.	[25,27,97]
	Serotonin	Decreased brain serotonin due to tryptophan depletion; linked to mood disorders, impulsivity, and suicidal behavior.	[148,258,263,272]
	Norepinephrine	Suppressed norepinephrine synthesis; impacts behavior, cognition, and immune regulation.	[265,266,268]
Hormones	Testosterone	Elevated testosterone in males; alters fear and sexual behavior, possibly linked to psychiatric outcomes.	[21,278,281]
	Vasopressin	Increased expression in male rodents; mediates loss of predator fear through interaction with testosterone.	[21,278]
	Oxytocin	Increased in infected females; modulates socioemotional behavior and may influence risk-taking and emotional regulation.	[292,297,298]
	Cortisol	Altered cortisol levels; may modulate anxiety and stress response with variable results across species and individuals.	[302,303,304]
Immune system	General immune effects	Pro- and anti-inflammatory cytokine shifts; impacts immune balance and may support parasite persistence.	[311,316]
	Physical health	Linked to cardiovascular, liver, kidney diseases, and autoimmune disorders via chronic inflammation.	[320,321,322,324]
	Cognitive and mental health	Inflammatory responses associated with cognitive impairments and psychiatric disorders.	[112,148,249]
	Reproductive effects	Possible immune suppression linked to altered sex ratios, Down syndrome prevalence, and prolonged pregnancies.	[198,207,337]

**Table 8:** Summary of findings—host and parasite factors modulating the effects of *Toxoplasma* infection.

Topic	Key Findings	References
Host sex—animal studies	Sex-specific behavioral effects; different immune responses and hormone influences; males and females may show opposite behaviors or mechanisms.	[41,206,278]
Host sex—human studies	Opposite personality and behavioral traits between sexes; infected men show lower sociality, women show higher conscientiousness. Sex-specific cognitive and mental health effects.	[19,71,109,122]
RhD status—reaction times	RhD-negative individuals more affected by toxoplasmosis; RhD-positive heterozygotes show resilience.	[114,115]
RhD status—cognitive/health outcomes	RhD-positive heterozygotes often show better mental/physical health. Some studies suggest RhD-positive homozygotes may fare worse than RhD-negative homozygotes.	[351,352,353]
RhD status—pregnancy and physical performance	Increased weight gain in RhD-negative, *Toxoplasma*-infected women. Better endurance in RhD-positive infected students.	[199,349]
Parasite strain—animal models	Some behavioral changes appear strain-general; for example, the loss of innate fear of cat odors, whilst other outcomes are strain-specific; for instance, Type II not Pru strain, induced memory deficits in mice	[360,361]
Parasite strain—human studies	Type I/atypical strains linked to more severe ocular and psychiatric outcomes; Type II less severe. Strain typing remains a challenge.	[369,370,376]

## Data Availability

No new data were created.

## Supplementary Materials

The following supporting information can be downloaded at https://www.mdpi.com/article/10.3390/biomedicines13071731/s1. Table S1. Summary of meta-analytical studies.
