# Supplementary material for: Beyond Latency: Chronic Toxoplasma Infection and Its Unveiled Behavioral and Clinical Manifestations—A 30-Year Research Perspective
Source: Biomedicines. 2025 Jul 15;13(7):1731. doi: 10.3390/biomedicines13071731 (PMC12292676; doi:10.3390/biomedicines13071731)
Supplement: Supplementary file 1 [file biomedicines-13-01731-s001.zip › biomedicines-3671833-supplementary.pdf]

Supplementary Table

Table S1 Summary of meta-analytical studies

Study / Author (Year)	Condition Studied	Key Metrics	Interpretation
Bisetegn et al. (2023)	Neuropsychiatric Disorders	IgG prevalence 38% (patients) vs. 25% (controls)	Markedly higher seroprevalence in patients; supports screening relevance.
Sutterland et al. (2015)	Multiple Psychiatric Disorders	OCD OR = 3.40, Addiction OR = 1.91, BD OR = 1.52, Schizophrenia OR = 1.43 (adjusted)	Significant links, strongest for OCD and schizophrenia.
Torrey et al. (2007)	Schizophrenia	OR ≈ 2.73 (95% CI 2.10–3.60)	*Toxoplasma* seropositivity significantly associated with schizophrenia; consistent across illness phases.
de Barros et al. (2017)	Bipolar Disorder	OR = 1.26	Modest but significant association.
Cossu et al. (2022)	Bipolar Disorder	ORs: 2.7–3.6 (France), ~3.0 (Ethiopia)	Broad evidence supports link; geographic variability present.
Nayeri Chegeni et al. (2019)	OCD	OR = 1.96 (95% CI: 1.32–2.90)	Latent toxoplasmosis nearly doubles OCD risk; consistent with biological plausibility.
Nayeri et al. (2020)	Autism Spectrum Disorder	Latent OR = 1.93; Acute OR = 0.39	Latent infection associated with increased ASD risk; acute may be protective or artifact.
de Haan et al. (2021)	Cognition	SMDs: ~0.12–0.18 across cognitive domains	Mild but consistent cognitive deficits in seropositive individuals.
Gohardehi et al. (2018)	Traffic Accidents	OR up to 16.0 (high titer); commonly ~2.65	Latent infection significantly raises accident risk, with dose–response pattern.

Sutterland et al. (2019)	Traffic Accidents, Suicide Attempts	TA OR = 1.69; SA OR = 1.39	Associations found, particularly stronger in non-schizophrenia psychiatric samples.
Abdoli et al. (2024)	Testosterone Levels	Mean difference: +0.73 (males), +0.55 (females)	Modest increase in testosterone linked with infection; animal data mixed.
Majidiani et al. (2016)	Diabetes	T2DM OR = 2.39; T1DM OR = 1.10 (NS)	*Toxoplasma* linked with T2DM, not T1DM.
Delhaes et al. (2010)	Congenital Toxoplasmosis	No ORs; case-based	Atypical strains linked with severe cases; genotyping advised.
Kalantari et al. (2021)	Spontaneous Abortion	Latent OR = 1.84; Recent OR = 3.72–4.20	Recent infection strongly associated with abortion; latent also a risk.
Tabares Tejada & Cardona Maya (2025)	Male Reproductive Health	No ORs; qualitative data	Animal evidence for sperm/endocrine impact; human link plausible but unproven.
